# Phase 1b study on the repurposing of meclizine hydrochloride for children with achondroplasia

**DOI:** 10.1371/journal.pone.0283425

**Published:** 2023-07-10

**Authors:** Masaki Matsushita, Hiroshi Kitoh, Kenichi Mishima, Yasunari Kamiya, Daisaku Kato, Genta Takemoto, Kenta Sawamura, Shinji Ueno, Nakai Yasuhiro, Kazuki Nishida, Shiro Imagama

**Affiliations:** 1 Department of Orthopaedic Surgery, Nagoya University Graduate School of Medicine, Nagoya, Japan; 2 Department of Orthopaedic Surgery, Aichi Children’s Health and Medical Center, Obu, Japan; 3 Department of Comprehensive Pediatric Medicine, Nagoya University Graduate School of Medicine, Nagoya, Japan; 4 Department of Ophthalmology, Nagoya University Graduate School of Medicine, Nagoya, Japan; 5 Department of Ophthalmology, Hirosaki University Graduate School of Medicine, Hirosaki, Japan; 6 Department of Advanced Medicine, Nagoya University Hospital, Nagoya, Japan; Springer Nature, UNITED STATES

## Abstract

Achondroplasia (ACH) is a common skeletal dysplasia characterized by a disproportionately short stature. We found that meclizine, which is an over-the-counter drug for motion sickness, inhibited the fibroblast growth factor receptor 3 (FGFR3) gene using a drug repositioning strategy, and meclizine 1 and 2 mg/kg/day promoted bone growth in a mouse model of ACH. A previous phase 1a clinical trial for children with ACH demonstrated that a single dose of meclizine 25 and 50 mg was safe and that the simulated plasma concentration achieved steady state approximately 10 days after the first dose. The current study aimed to evaluate the safety and pharmacokinetics (PK) of meclizine in children with ACH after a 14-day-repeated dose of meclizine. Twelve patients with ACH aged 5–10 years were enrolled. Meclizine 12.5 (cohort 1) and 25 mg/day (cohort 2) were administered after meals for 14 days, and adverse events (AEs) and PK were evaluated. No patient experienced serious AEs in either group. The average (95% confidential interval [CI]) maximum drug concentration (C_max_), peak drug concentration (T_max_), area under the curve (AUC) from 0 to 24 h, and terminal elimination half-life (t_1/2_) after a 14-day-repeated administration of meclizine (12.5 mg) were 167 (83–250) ng/mL, 3.7 (3.1–4.2) h, 1170 (765–1570) ng·h/mL, and 7.4 (6.7–8.0) h, respectively. The AUC_0-6h_ after the final administration was 1.5 times that after the initial dose. C_max_ and AUC were higher in cohort 2 than in cohort 1 in a dose-dependent manner. Regarding the regimen of meclizine 12.5 and 25 mg in patients < 20 kg and ≥ 20 kg, respectively, the average (95% CI) AUC_0-24h_ was 1270 (1100–1440) ng·h/mL. Compartment models demonstrated that the plasma concentration of meclizine achieved at a steady state after the 14th administration. Long-term administration of meclizine 12.5 or 25 mg/day is recommended for phase 2 clinical trials in children with ACH.

## Introduction

Achondroplasia (ACH) is a skeletal dysplasia characterized by shortened limb stature caused by gain-of-function mutations in fibroblast growth factor receptor 3 (FGFR3) [1, 2]. Growth hormone has been administered to children with ACH in some countries, but its effect on bone elongation was limited [3]. Limb-lengthening surgery could increase the height of patients with ACH but reduce the activities of daily living of patients during the treatment period [4]. Vosoritide, which is a C-type natriuretic peptide analog that can inhibit FGFR3 signaling, has been developed for clinical application and has been approved in some countries [5–7]. Vosoritide is a potential burden on children with ACH because it requires subcutaneous administration. There are other FGFR3 inhibitors under development, including soluble FGFR3 [8, 9], tyrosine kinase inhibitors [10], and FGF2 aptamer [11].

By using a drug repositioning strategy, we found that meclizine hydrochloride (meclizine) inhibited FGFR3 signaling in chondrocytes [12]. Pharmacokinetics (PK) after a single dose of meclizine 2 mg/kg in mice revealed that the maximum drug concentration (C_max_) and area under the curve (AUC) were lower than those of the dose used in clinical settings (25 mg/d) [13, 14]. After the administration of meclizine 25 mg/d to healthy adult humans, the time to peak drug concentration (T_max_), C_max_, AUC_0-24h_, and terminal elimination half-life (t_1/2_) were 3.11 h, 68.4 ng/mL, 446.5 ng·h/mL, and 5.11 h, respectively [14]. In children with ACH, the T_max_, C_max_, AUC_0-24h_, and t_1/2_ after the administration of meclizine 25 mg/d were 1.7 h, 130 ng/mL, 761 ng·h/mL, and 8.5 h, respectively, [15]. Meclizine at 1 or 2 mg/kg/day promoted bone growth in a mouse model of ACH in a dose-dependent manner. In addition, both doses of meclizine ameliorated bone quality in mice with ACH. Therefore, clinically attainable concentrations of meclizine could potentially increase the bone length in patients with ACH.

Although meclizine has been used as both a prescription drug and an over-the-counter drug for over 50 years, there is insufficient safety data to facilitate performance of clinical trials. In conformance with the stipulations of the instruction by the Pharmaceuticals and Medical Devices Agency (PMDA), we performed a phase 1a clinical trial in 12 children with ACH after preclinical safety studies on the basis of current regulatory requirements for repeated administration according to International Conference on Harmonization of Technical Requirements for the Registration of Pharmaceuticals for Human Use guideline M3, including 2-week repeated-dose toxicity studies, toxicokinetic and PK studies, genotoxicity studies, and safety pharmacology and pharmacodynamic studies [15]. After juvenile animal toxicity studies were additionally conducted, the PMDA approved the phase 1b clinical trial.

The current phase 1b open-label study (jRCT2041200114) investigated the safety and PK profile of a two-week-repeated dose of meclizine in children with ACH. We can subsequently proceed to phase 2 clinical trials after the completion of the current clinical trial and long-term repeated-dose toxicity studies.

## Materials and methods

### Study design

This study had an open-label design to evaluate the safety and PK of a 14-day administration of meclizine with two ascending dose cohorts. The study design was assessed by the PMDA before study initiation on the basis of the results of preclinical studies and phase 1a clinical trials. The current study was conducted at Nagoya University Hospital and Aichi Children’s Health and Medical Center under the Japanese Pharmaceuticals Affairs Low and Good Clinical Practice defined by the Ministry of Health, Labour and Welfare in Japan.

The study protocol and all amendments were approved by the institutional review boards of Nagoya University Hospital and Aichi Children’s Health and Medical Center. This study was registered in the Japan Registry of Clinical Trials (jRCT: ID: jRCT2041200114) (https://jrct.niph.go.jp/latest-detail/jRCT2041200114). The legal guardian of each participant provided written informed consent, and participants above six years old signed an agreement before the commencement of the study procedures.

Patients eligible for enrollment were children aged 5–10 years who were clinically diagnosed with ACH on the basis of the Japanese diagnostic criteria for ACH or genetically diagnosed (*FGFR3* mutations) at 1 year or more before obtaining consent. The exclusion criteria included previous exposure to meclizine within 28 days preceding this study; surgical treatment for limb lengthening during the study period; weight < 11 kg; serious complications such as neurological impairments, clinically significant dysuria, history of glaucoma, known hypersensitivity, or allergies to meclizine; and regular oral medication containing cold medicine, antipyretic analgesic, sedative, antitussive expectorant, and anti-histamine. In addition, non-steroidal-anti-inflammatory drugs and various medicines containing antihistamines were prohibited from 24 h before treatment to 24 h after the administration of the final dose. Anti-motion-sickness drugs containing meclizine and reported FGFR3 inhibitors were also prohibited from 7 days preceding treatment to the end of the study.

The primary objective of our study was to evaluate the incidence of adverse events (AEs) up to 21 days after the first dose. The secondary objective was to evaluate the PK parameters (T_max_, C_max_, AUC, and t_1/2_).

### Selection of doses

**Fig 1:**
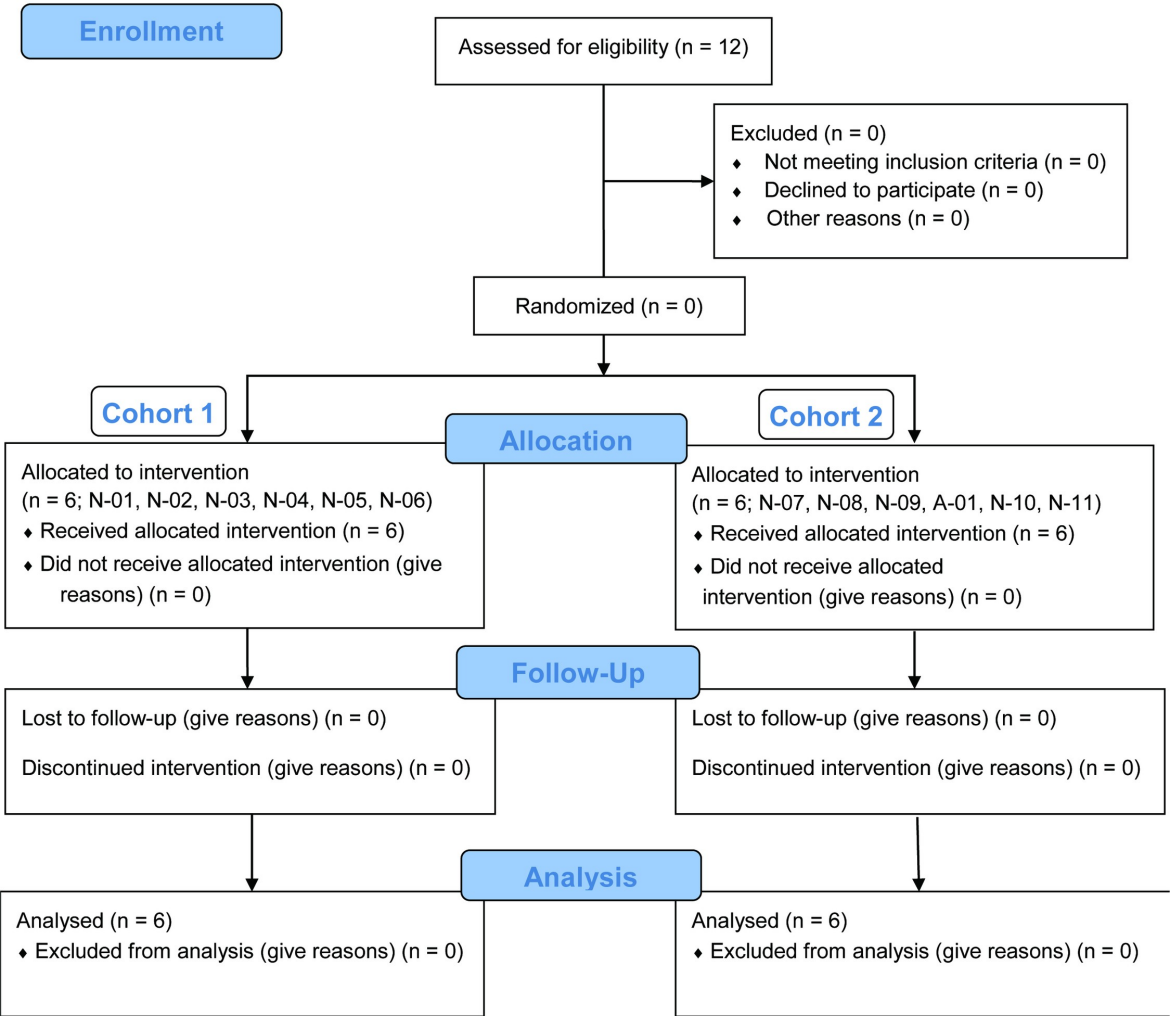
CONSORT flowchart. Patients (n = 12) were screened, and there were no patients with failed treatment. The 12 patients were divided into cohorts 1 and 2, and all patients completed treatment. N: Nagoya University Hospital. A: Aichi Children’s Health and Medical Center.

**Fig 2:**
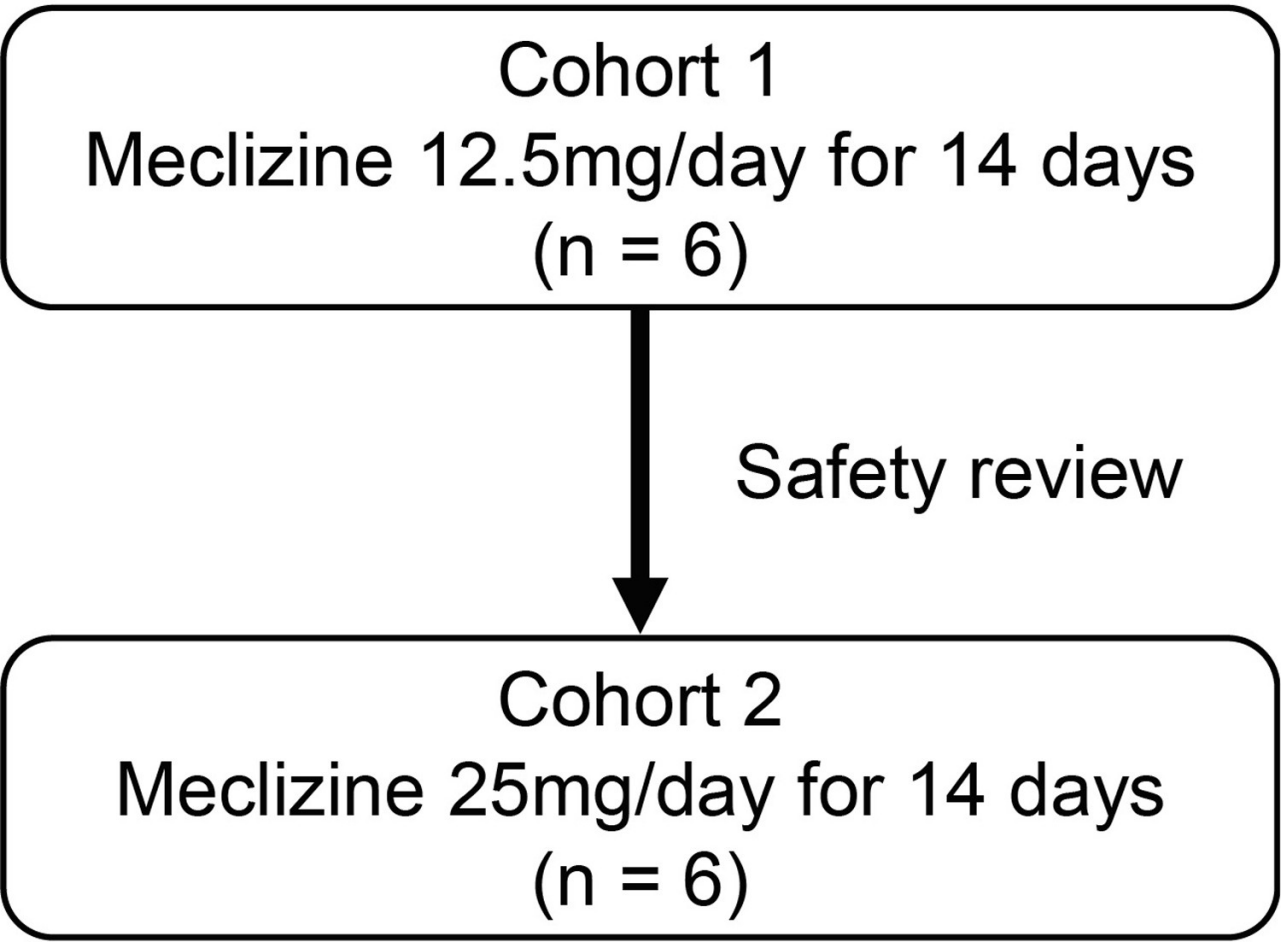
The dose ascending process in the phase 1b clinical trial of the 14-day repeated administration of meclizine. After the administration of meclizine 12.5 mg/day in cohort 1, we performed a safety review before proceeding with the administration of meclizine 25 mg/day in cohort 2.

Dose-finding studies in animal models of ACH indicated that meclizine at 1 and 2 mg/kg/day increased longitudinal bone length in a dose-dependent manner, whereas meclizine 20 mg/kg/day exhibited no effect on bone length [13]. The C_max_ after the administration of meclizine 2 mg/kg in mice was 60.7 ng/mL, which was below the C_max_ of meclizine 25 mg/d in adult humans [14] and children with ACH [15]; however, the C_max_ of meclizine 20 mg/kg in mice was 1020 ng/mL. Therefore, the doses were selected to achieve approximately 60 ng/mL of exposure in children with ACH as oral tablets of meclizine 12.5 mg/day (cohort 1) or 25 mg/day (cohort 2). The schema of the phase 1b study is shown in Fig 1. Patients with ACH participated in only one dose cohort and subsequently received meclizine for 14 days (Fig 2).

### Investigational medicine

Meclizine tablets were provided as one or two hard 12.5 mg tablets in each package manufactured by Meiji Yakuhin Co. Ltd., Japan. The tablets contained D-mannitol, cellulose, carmellose sodium, hardened oil, magnesium stearate, and l-menthol as excipients and were stored at room temperature during the 36 months of validity. Meclizine tablets were verified as test drugs at Nagoya University Hospital according to Good Manufacturing Practices for clinical investigational products.

### Study assessments

The assessments comprised a screening period (days -28 to -1), a treatment period (days 1 to 14), and a follow-up period (days 15 to 21) (Tables 1 and 2). The participants visited Nagoya University Hospital or Aichi Children’s Health and Medical Center during the screening period on days 1, 8, 14, and 21. During the screening period, the participants underwent 12-lead electrocardiography (ECG); chest radiography; mydriatic slit-lamp microscopy; and assessment of laboratory parameters including haematology, blood chemistry, and urinalysis. Meclizine was administered in tablet form with 150 mL of water within 30 min after dinner at their home on days 2–13 because there was a potential risk of somnolence when the patients received it during daytime. On days 1 and 14, meclizine 12.5 or 25 mg was administered after eating breakfast in our hospitals to avoid any tests during the night. Each participant was consistently instructed regarding the time of meal, drug administration, and prohibited drugs.

**Table 1:** Schedule.

	Screening period	Treatment period					Follow-up period	
	Day -28 to -1	Day 1	Day 2 to 7	Day 8	Day 9 to 13	Day 14	Day 15	Day 21
Permissible range (day)	0	0	0	0	0	0	0	±1
12-lead electrocardiography (ECG)	○	●		○		●	●	○
Chest radiology	○							
Mydriatic slit-microscopy	○							○
Hematology	○	○		○		○	○	○
Blood chemistry	○	○		○		○	○	○
Urinalyses	○	○		○		○	○	○
Plasma concentration of meclizine		●		○		●	●	○

○ and ● indicate the timing of analysis. The detail timing of ● is shown in Table 2.

**Table 2:** Detailed timing of the 12-lead electrocardiography and meclizine concentration.

	Day 1						Day 14							Day 15
Time after meclizine administration (h)	-0.5	1	2	3	4	6	-0.5	1	2	3	4	6	10	24
Permissible range (min)	±30	±10	±10	±10	±10	±30	±30	±10	±10	±10	±10	±30	±30	±30
12-lead electrocardiography (ECG)	○			○						○				○
Plasma concentration of meclizine	○	○	○	○	○	○	○	○	○	○	○	○	○	○

○ indicates the timing of analysis.

For safety assessments, any AEs were collected on the basis of physical examinations, vital signs (body temperature, blood pressure, and pulse rate), and laboratory parameters during the screening period and days 1, 8, 14, and 21. ECG was performed during the screening period; 3 h after the administration of meclizine on days 1 and 14; and on days 8, 15, and 21. Mydriatic slit-lamp microscopy was performed during the screening period and on day 21. All AEs reported by the subjects or detected in the assessment were recorded, and the investigators determined their relationship with the treatment. The terminology and severity of AEs were determined based on the Common Terminology Criteria for Adverse Events (CTCAE v5.0/MedDRA/J v23.1). Safety data were summarized descriptively and presented in a tabular format.

### Sample management

For the PK assessments, blood samples (2 mL per sample) were collected immediately before drug administration; at 1, 2, 3, 4, and 6 h after administration on days 1 and 14; and at 10 and 24 h on day 14 (Tables 1 and 2). Catheters were used to prevent repeated needle use. The plasma concentration of meclizine was measured on days 8 and 21. Blood samples were collected in tubes containing heparin sodium as an anticoagulant. Plasma was separated by centrifugation for 10 min at 3,000 rpm at room temperature. Subsequently, the separated plasma samples were transferred into two polypropylene tubes and stored at approximately -40°C until analysis. Plasma concentrations of meclizine were determined using validated liquid chromatography–tandem mass spectrometry assays with a calibration range of 0.5–200 ng/mL at Ina Research Inc., Nagano, Japan, according to a previous study [15].

### Pharmacokinetics analyses

PK parameters were calculated for each subject by using the non-compartmental analysis of plasma concentration–time data by the Phoenix WinNonlin version 6.1 software (Pharsight, Mountain View, CA, USA): C_max_, T_max_, t_1/2_, AUC_0-6_ and AUC_0-24_. PK parameters were evaluated using a non-compartmental model of WinNonlin.

The compartment analysis of the plasma concentration of each patient was performed using MODEL 14 in Phoenix WinNonlin version 6.1 software. MODEL 14 describes a two-compartment model with an oral administration and lag time of absorption. Dose as mg/d, dose number as times, and administration timing as time after the first administration were analyzed as WinNonlin constants without weighting. All plasma concentrations and sampling times after the first administration were entered into the WinNonlin dataset.

According to a phase 1a clinical trial [15], PK parameters were adjusted according to the patients’ averaged weight. Correlation analysis was performed between body weight and AUC by using SPSS version 25 (IBM Corporation, Armonk, NY, USA). AUC according to the proposed regimen was calculated as follows: the AUC was reduced by half if patients < 20 kg received meclizine 25 mg or increased by 2-fold if patients ≥ 20 kg received meclizine 12.5 mg.

### Statistics

Although the formal sample size was not calculated, six participants per cohort without placebo control were used to fulfill the study objectives based on previous PK studies. All patients who received meclizine were included in the safety analysis. AEs and other safety analyses were performed using SAS version 9.4 software (SAS Institute Inc., Cary, NC, USA). The incidence and 95% confidence interval (CI) of each AE were calculated according to the Clopper–Pearson exact method. All subjects who completed all measurements of the plasma concentration of meclizine were included in the PK analysis. The coefficient of variation was calculated as the average divided by the standard deviation (SD) to assess the dispersion.

## Results

### Subjects

Eleven patients were screened and enrolled at Nagoya University Hospital, and one patient was from Aichi Children’s Health and Medical Center (Fig 1). No patients were excluded, and all enrolled patients were included in the current clinical trial. The baseline characteristics of the participants are presented in Table 3. There were 7 male and 5 female patients aged 5–10 years old who were diagnosed with ACH. The ranges for height, sitting height, and weight were 86.4–116.3 cm, 57.4–78.7 cm, and 14.4–48.1 kg, respectively. In cohort 2, the SD of the patients’ weights was large. The compliance with the meclizine dosing schedule was 100.0%.

**Table 3:** Summary of subject baseline characteristics.

Characteristics		Cohort 1	Cohort 2
		(n = 6)	(n = 6)
Male/ Female (n)		3/3	4/2
Age (years)	Mean ± SD	7.0 ± 1.7	7.2 ± 1.8
	Median	7.0	7.5
	Range	5–10	5–9
Height (cm)	Mean ± SD	97.37 ± 8.12	103.72 ± 12.70
	Median	93.9	107.65
	Range	90.1–112.5	86.4–116.3
Sitting height (cm)	Mean ± SD	66.72 ± 3.87	69.77 ± 8.49
	Median	66.15	71.20
	Range	62.2–72.9	57.4–78.7
Weight (kg)	Mean ± SD	18.70 ± 2.30	26.05 ± 12.03
	Median	17.80	24.05
	Range	17.0–23.1	14.4–48.1

### Safety of meclizine

The number of patients (rate, 95% CI) experienced AEs were two (33.3%, 4.3–77.7) in cohort 1 and one (16.7%, 0.4–64.1) in cohort 2, respectively (Table 4). The reported AEs were mild (grade 1), because they were resolved spontaneously. Grade 1 AEs included upper abdominal pain (one event in one patient), vomiting (two events in two patients), and somnolence (one event in one patient). The mouth ulceration (one event in one patient) was classified as moderate CTCAE (grade 2) because ointment was required for treatment.

**Table 4:** Adverse events classified by grades.

		Grade 1	Grade 2	Grade 3	Grade 4	Grade 5
Cohort 1	Gastrointestinal disorders	2 (3 events)	1 (1 event)	0	0	0
(n = 6)	Upper abdominal pain	1 (1 event)	0	0	0	0
	Mouth ulceration	0	1 (1 event)	0	0	0
	Vomiting	2 (2 events)	0	0	0	0
Cohort 2	Nervous system disorders	1 (1 event)	0	0	0	0
(n = 6)	Somnolence	1 (1 event)	0	0	0	0

The repeated administration of meclizine demonstrated no clinically significant effects on vital signs, including body temperature, blood pressure, and pulse rate (S1 Table) in addition to confirming the patients’ general conditions under the interview by hospital’s staffs. There were no significant changes in ECG, mydriatic slit-lamp microscopy, haematology, blood chemistry, and urinalysis. No serious AEs (SAEs) related to the study medication, withdrawal due to AEs, or any other issue were observed.

### PK profile of meclizine

The PK profile of meclizine on days 1 and 14 in pediatric patients with ACH is summarized in Table 5. The average T_max_ values were generally comparable on days 1 and 14 at both dose levels. C_max_ and AUC on both days increased with increasing doses. The average t_1/2_ across both doses averaged approximately 7–8 h on day 14. AUC_0-6h_ exhibited slight accumulation with dosing. The SD of C_max_ and AUC appeared to be larger in cohort 2 than in cohort 1.

**Table 5:** Pharmacokinetics after meclizine administration.

		Cohort 1 (n = 6)		Cohort 2 (n = 6)	
		Mean ± SD	95% CI	Mean ± SD	95% CI
Day 1	T_max_ (hr)	2.7 ± 1.2	1.4–3.9	3.0 ± 0.9	2.1–3.9
	C_max_ (ng/ml)	133 ± 43	88–178	283 ± 212	60–506
	AUC_0-6h_ (ng·h/ml)	392 ± 132	254–530	766 ± 466	297–1230
Day 14	T_max_ (hr)	3.7 ± 0.5	3.1–4.2	2.8 ± 1.0	1.8–3.9
	C_max_ (ng/ml)	167 ± 79	83–250	266 ± 140	118–413
	t_1/2_ (hr)	7.4 ± 0.6	6.7–8.0	7.9 ± 1.2	6.6–9.1
	AUC_0-6h_ (ng·h/ml)	577 ± 182	384–768	897 ± 477	396–1400
	AUC_0-24h_ (ng·h/ml)	1170 ± 380	765–1570	1780 ± 1010	720–2840

**Fig 3:**
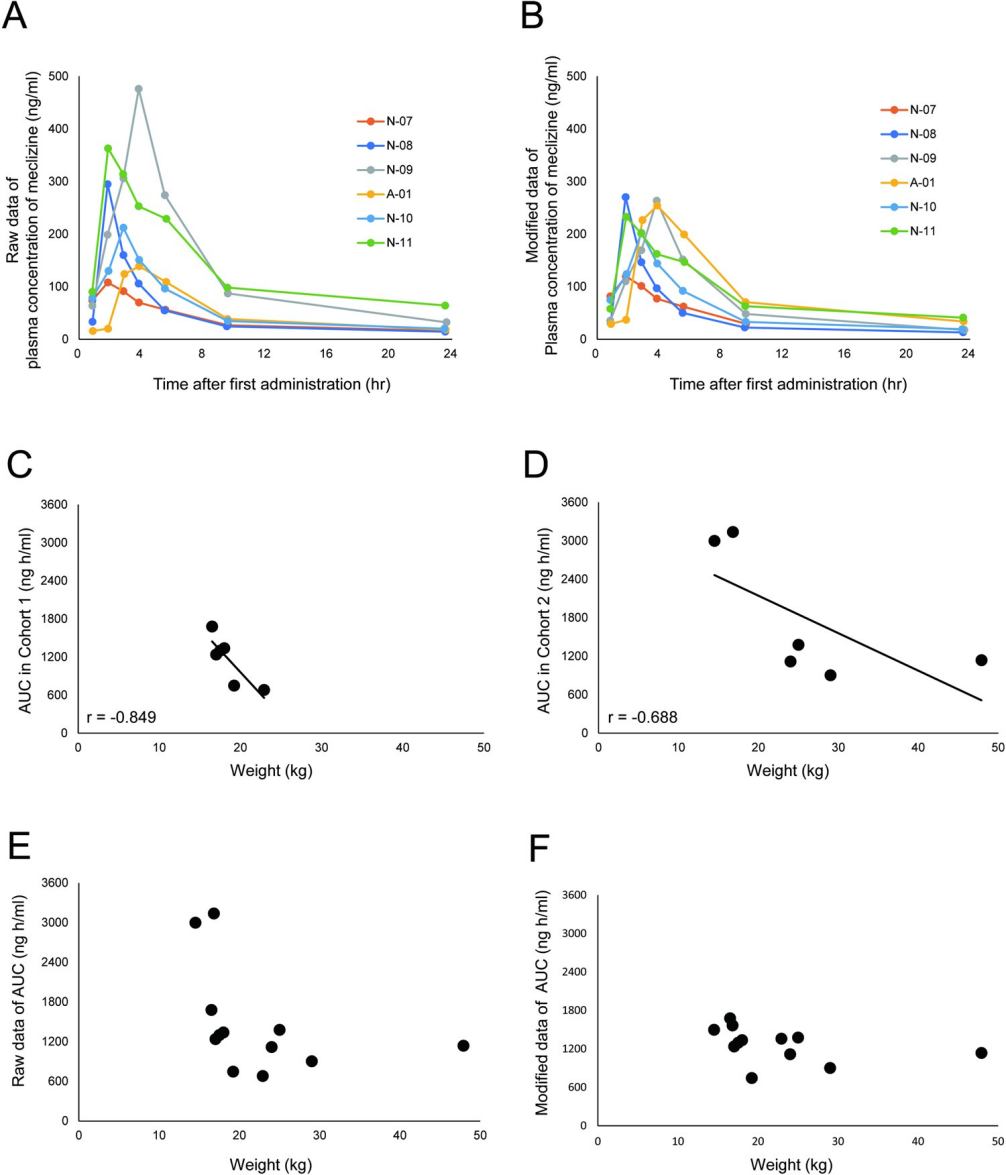
PK profile related to weight. Raw data of individual plasma concentration profiles of meclizine on day 14 in cohort 2 (A), and modified data of plasma concentration profiles adjusted by the averaged body weight of cohort 2 (26.05 kg) (B).　The scatter diagram illustrates the patients’ weight and AUC_0-24h_ on day 14 in cohort 1 (C) and cohort 2 (D). The linear regression line is shown. The scatter diagram demonstrates individual weight and raw data of AUC_0-24h_ on day 14 in all patients (E). Individual weight and modified data of AUC_0-24h_ on day 14 if the following proposed regimen was applied: assuming the administration of meclizine 12.5 and 25 mg in patients < 20 kg and ≥ 20 kg, respectively (F). N: Nagoya University Hospital. A: Aichi Children’s Health and Medical Center. AUC: area under curve.

The plasma concentration of meclizine in each patient was uneven on day 14 in cohort 2 (Fig 3A). After performing adjustment by using the average weight of cohort 2 according to our previous study (phase 1a clinical trial) [15], the average (95% CI) C_max_ and AUC_0-6h_ were 229 (132–327) ng/mL and 654 (419–888) ng·h/mL, respectively (Fig 3B). Given that the scatter deviation became smaller after performing adjustment by using the weight, the relation between the patients’ weight and exposure was evaluated. The AUC_0-24h_ on day 14 was negatively correlated with patient weight (Fig 3C and 3D). The proposed regimen of 25 mg for patients ≥ 20 kg and 12.5 mg for patients < 20 kg would reduce the coefficient of variation value for AUC from 54 (Fig 3E) to 21% (Fig 3F).

**Fig 4:**
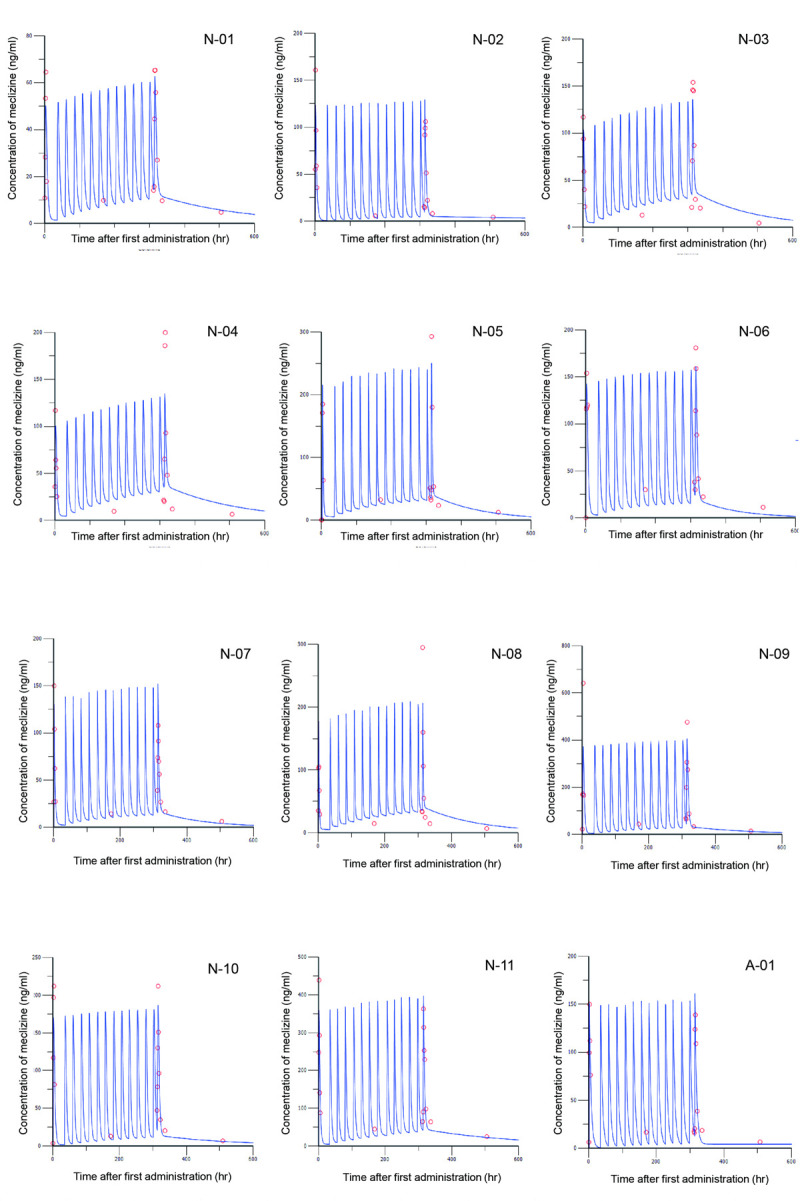
PK compartment model of meclizine in each patient. The plasma concentration of meclizine apparently reached a steady state approximately 14 days after the first dose. Observed (red circle) and model-predicted (blue curves) concentration–time data were indicated. N: Nagoya University Hospital. A: Aichi Children’s Health and Medical Center.

The compartment models demonstrated good fitting (Fig 4). The moment analysis using compartment model parameters indicated that the effective half-life for cohorts 1 and 2 were 49 and 36 h, respectively. Therefore, the plasma concentration of meclizine achieved a steady state on approximately the 14th day of administration.

## Discussion

The current phase 1b clinical trial with a 14-day-repeated dose of meclizine was well tolerated and exhibited acceptable PK profiles in children with ACH aged 5–10 years in both cohorts administrated meclizine 12.5 or 25 mg/day.

Several clinical studies have demonstrated the safety of meclizine administration. Repeated high doses of meclizine have been reported to cause nausea and vomiting during pregnancy (NVP). In a Norwegian study of patients with NVP, 36 and 10 pregnant women continued to receive meclizine for 5 weeks to 3 months and at more than 4 months of gestation, respectively [16]. Another Norwegian study indicated that NVP improved in 26 pregnant women after repeated meclizine doses for 56 days [17]. There were no statistical differences in the rates of early fetal death and congenital abnormalities between women with and without meclizine administration during pregnancy [18]. Another Swedish study of 20,902 pregnant women revealed that meclizine administration did not increase the risk of premature birth, low birth weight, or fetal growth retardation [19]. There was no description regarding severe AEs after repeated doses of meclizine 25 mg/day for 3 months in patients with developmental dyslexia aged 9–14 years [20].

In the present study, no patients experienced SAEs after a 14-day-repeated dose of meclizine, and there was no relationship between the frequency or severity of AEs and increasing doses. The gastrointestinal symptoms were observed only in a lower-dose group (cohort 1). In this group, a patient with a low exposure of meclizine was included. In addition, gastrointestinal symptoms, including upper abdominal pain, mouth ulceration, and vomiting, have not been reported as adverse effects of meclizine, and these symptoms appeared when participants were nervous during the blood tests and immediately disappeared after the blood tests were completed. Therefore, the AEs that developed in this study were not considered related to meclizine according to the judgement committee. Although the patient reported that somnolence did not require a nap, he took a nap after the initial administration of meclizine. Therefore, somnolence was the only AE that could not be ruled out as having a causal relationship with meclizine, which was similar to that observed after a single dose [15]. Except for this case, other patients did not develop somnolence despite the anti-histamine property of meclizine.

According to the results of animal studies using a mouse model of ACH, meclizine needs to be administered daily to maintain the meclizine concentration and promote bone growth by inhibiting FGFR3 signaling [13]. In the same dose regimen of 25 mg/day under a fed condition, the PK parameters of meclizine were similar. The average T_max_ and C_max_ after first administration in phase 1a study were 2.6 h and 223 ng/mL, respectively [15]. In the current study, the T_max_ and C_max_ were 2.7 h and 283 ng/mL, respectively. Furthermore, steady state estimation was also similar. The result of the phase 1a study indicated that plasma concentration reached a steady state on approximately the 10th day after first administration [15], whereas it was on approximately the 14th day in the current study. The proposed regimen, namely, the respective administration of meclizine 12.5 and 25 mg in patients < 20 kg and ≥ 20 kg, indicated that the AUC_0-24h_ was 2.7 times as much as the AUC_0-24h_ after a single dose of meclizine 25 mg/d in adult humans without diet restriction [14]. These results indicate that a once daily administration of meclizine at a dose of 12.5 or 25 mg/day maintains plasma levels above pharmacological levels.

## Conclusions

The current phase 1b clinical trial of repeated doses of meclizine in children with ACH demonstrated favorable safety and PK profiles. The repeated dose of meclizine 12.5 and 25 mg/day according to weight is likely to be a clinically attainable concentration for the promotion of bone growth in patients with ACH.

## Supporting information

S1 Checklist TREND checklist. (PDF)

S1 File (DOCX)

S2 File Original study protocol. (DOCX)

S1 Table Summary of vital signs at each time point. (DOCX)
