# Supplementary material for: Phase 1b study on the repurposing of meclizine hydrochloride for children with achondroplasia
Source: PLoS One. 2023 Jul 10;18(7):e0283425. doi: 10.1371/journal.pone.0283425 (PMC10332602; doi:10.1371/journal.pone.0283425)
Supplement: S1 File — (DOCX) [file pone.0283425.s002.docx]

**Clinical trial protocol**

**Safety and pharmacokinetics of 2-week continuous administration of meclizine hydrochloride in** **pediatric patients with achondroplasia**

**Clinical Trial Coordinating Physician**

Nagoya University Hospital ・ Orthopedics

Hospital Lecturer Masaki Matsushita

**Clinical Trial Coordination Office**

Department of Advanced Medical Development, Nagoya University Hospital

Yasuhiro Nakai

Postal Code 466-8560 65 Tsurumai-cho, Showa-ku, Nagoya City

TEL：052-744-2942 FAX：052-744-1303

Implementation Plan No. CAMCR-015

Edition Number / Creation Date Version 4.0 November 2, 2021

# List of abbreviations and definitions of terms

List of abbreviations

| abbreviation | Meaning |
| --- | --- |
| ACH | Achondroplasia (chondrodysplasia) |
| AUC | Area under the curve |
| Cmax | Maximum concentration |
| CNP | C-type natriuretic peptide |
| CRF | Case report form |
| FAS | Full analysis set (largest analysis set) |
| FGFR3 | Fibroblast growth factor receptor 3 |
| GCP | Good clinical practice (Standards for the Conduct of Clinical Trials of Drugs and Medical Devices) |
| PPS | Per protocol set (study protocol compliance population) |
| Tmax | Time to reach Cmax |
| t 1/2 | Elimination half-life |
|  |  |

List of Abbreviations for Clinical Laboratory Test Items

| mark | Unabbreviated expression terms |
| --- | --- |
| TP | Total protein |
| Alb | Albumin |
| ALT | Alanine aminotransferase |
| AST | Aspartate aminotransferase |
| BUN | Blood urea nitrogen |
| Cl | Chlorine |
| Cre | Creatinine |
| CRP | C-reactive protein |
| K | Potassium |
| Na | Sodium |
| TG | Triglyceride(s) |

For word list

| Terminology | Definition |
| --- | --- |
| Ministerial Ordinance on GCP for Pharmaceuticals | Ministerial Ordinance Concerning Standards for the Conduct of Clinical Trials of Pharmaceuticals (Ordinance of the Ministry of Health, Labour and Welfare No. 28 of March 27, 1997) |
| Declaration of Helsinki | World Medical Association Declaration of Helsinki (revised October 2013) |
|  |  |

[List of abbreviations and definitions of terms 1](#_Toc127747405)

[Summary 5](#_Toc127747406)

[1. Purpose of the clinical trial 11](#_Toc127747407)

[1.1. Test design 11](#_Toc127747408)

[1.2. Primary endpoints 11](#_Toc127747409)

[2. Background and rationale for the clinical trial 11](#_Toc127747410)

[2.1. About achondroplasia 11](#_Toc127747411)

[2.3. About investigational new drugs 12](#_Toc127747412)

[2.4. Efficacy of the investigational drug in achondroplasia 13](#_Toc127747413)

[2.5. Conduct of this clinical trial 13](#_Toc127747414)

[3. Overview of the investigational drug 14](#_Toc127747415)

[3.1. Investigational drugs 14](#_Toc127747416)

[3.2 Formulation composition of the investigational drug 14](#_Toc127747417)

[3.3 Packaging and Labeling 14](#_Toc127747418)

[3.4 Handling, storage, and control of the investigational drug 15](#_Toc127747419)

[4. Research plan and design 15](#_Toc127747420)

[4.1. Type and design of clinical trial 15](#_Toc127747421)

[4.2 Outline of the clinical trial 15](#_Toc127747422)

[5. Eligible patients and eligibility criteria 16](#_Toc127747423)

[5.1. Selection criteria 16](#_Toc127747424)

[5.2. Exclusion criteria 16](#_Toc127747425)

[6 Registration 17](#_Toc127747426)

[6.1. Registration procedure 17](#_Toc127747427)

[6.2. Precautions for registration 17](#_Toc127747428)

[6.3. If you are determined to be ineligible as a subject for this clinical trial 18](#_Toc127747429)

[7. Method of the clinical trial 18](#_Toc127747430)

[7.1. Dosage and administration 18](#_Toc127747431)

[7.2. Study duration for each subject 18](#_Toc127747432)

[7.3. Use of investigational drug after completion of clinical trial 19](#_Toc127747433)

[7.4. Regulations regarding concomitant medications and concomitant therapies 19](#_Toc127747434)

[8. Evaluation items 20](#_Toc127747435)

[8.1. Primary endpoints 20](#_Toc127747436)

[Observation and inspection items and timing 21](#_Toc127747437)

[9.1. Observation and examination items 23](#_Toc127747438)

[9.2. Observation and examination methods 26](#_Toc127747439)

[10. Criteria for discontinuance 27](#_Toc127747440)

[11. Adverse events 27](#_Toc127747441)

[11.1. Response to subjects in the event of adverse events, and investigation and reporting of adverse events 27](#_Toc127747442)

[11.2 Definitions of adverse events, etc. 28](#_Toc127747443)

[11.3. Actions to be taken in case of serious adverse events 30](#_Toc127747444)

[11.4. Adverse events expected in this clinical trial 30](#_Toc127747445)

[11.5 Adverse event recording procedures 31](#_Toc127747446)

[11. 6. Provide new information 32](#_Toc127747447)

[12. Efficacy and Safety Evaluation Committee 33](#_Toc127747448)

[13. Termination, suspension or discontinuation of the clinical trial 33](#_Toc127747449)

[13.1. Termination of the clinical trial 33](#_Toc127747450)

[13.2. Discontinuation or suspension of the entire clinical trial 33](#_Toc127747451)

[14. Target number of patients and trial duration 34](#_Toc127747452)

[14.1. Target number of cases 34](#_Toc127747453)

[14.2. Duration of the clinical trial 34](#_Toc127747454)

[15. Statistical analyses 34](#_Toc127747455)

[15.1. Population to be analyzed 34](#_Toc127747456)

[15.2 Analysis Items and Methods 35](#_Toc127747457)

[15.3. Modification of statistical analysis plan 35](#_Toc127747458)

[15.4. Final analysis 35](#_Toc127747459)

[16. Quality control and quality assurance 35](#_Toc127747460)

[17. Ethical matters 36](#_Toc127747461)

[17.1. Rules and regulations to be observed 36](#_Toc127747462)

[17.2. Compliance with the study protocol 36](#_Toc127747463)

[17.3. Changes to clinical trial protocols, etc. 36](#_Toc127747464)

[17.4. Deviations from the clinical trial protocol 36](#_Toc127747465)

[17.5. Preparation and revision of the explanation and consent documents 37](#_Toc127747466)

[17.6. Informed consent 38](#_Toc127747467)

[17.7. Protection of personal information 39](#_Toc127747468)

[18. Cost sharing for clinical trials 39](#_Toc127747469)

[18.1. Costs, sources of funding and financial relationships related to the clinical trial 39](#_Toc127747470)

[18.2. Measures against health hazards 40](#_Toc127747471)

[18.3. Payment of money to test subjects 40](#_Toc127747472)

[19. Method of preservation and use of samples, etc. and period of preservation 40](#_Toc127747473)

[19.1. Preservation of samples, etc. 40](#_Toc127747474)

[19.2. Record keeping 40](#_Toc127747475)

[20. Enrollment and progress reporting of clinical trials 42](#_Toc127747476)

[21. Attribution of clinical trial results and publication of results 42](#_Toc127747477)

[22. Organization and structure for conducting clinical trials 42](#_Toc127747478)

[23. References 43](#_Toc127747479)

# Summary

| **Clinical Trial Coordinating Physician:**  Masaki Matsushita, Hospital Lecturer, Department of Orthopaedic Surgery, Nagoya University Hospital |
| --- |
| **Issue Title.**  Safety and pharmacokinetics of meclizine hydrochloride after 2 consecutive weeks of administration to pediatric patients with achondroplasia |
| **Investigational drug: (1)**  1. Generic name: Meclizine hydrochloride  2. Name of the investigational drug: MECLIZINE  3. Ingredients and quantity: Each tablet contains 12.5 mg of meclizine hydrochloride, with D-mannitol, cellulose, sodium carmellose, hardened oil, magnesium stearate, and l-menthol as additives.  4. Dosage form: tablet |
| **Implementation Plan No.**  CAMCR-015 |
| **Number of medical institutions**  Two facilities (Nagoya University Hospital and Aichi Children's Health Care Center) |
| **Study design:**  Multicenter, open-label, single-arm trial |
| **Main objectives:**  Meclizine hydrochloride will be administered once daily for 14 days to pediatric patients with achondroplasia, and pharmacokinetics will be studied on the first and last day of administration, as well as safety |
| **Target diseases:**  Patients with achondroplasia (ACH) |
| **Target number of cases:**  6 cases per group, 2 groups, 12 cases in total |
| **Basis for setting the number of cases:**  Given that the main objective of this clinical trial is to confirm the safety and pharmacokinetics of meclizine hydrochloride, a statistically based case-count design will not be employed.  The target number of patients is 6 per group, as this number of patients has been used in similar clinical trials |
| **Clinical trial duration: 1 year**  The overall duration of the clinical trial: March 2021–March 2022  Case enrollment period: April 2021–January 2022  Subjects' study period: approximately 49 days (28 days for obtaining consent and screening + 14 days for administration + 7 days for follow-up) |
| **Selection Criteria:**  Patients who meet all of the following conditions:   1. Patients with a confirmed diagnosis of ACH that meets definite or probable criteria established by the Ministry of Health, Labour and Welfare Intractable Disease Study Group at least one year prior to obtaining consent. 2. Patients aged 5 to 11 years at the time consent is obtained 3. Patients who can be admitted to the hospital on the last day of administration and are expected to complete the study 4. Patients for whom written consent has been obtained from a surrogate 5. Patients ≥7 years with written consent from the subject 6. Patients who can take tablets   [Rationale]   1. The patient population was established as the target patient population for treatment. 2. The pharmacological effects of the test drug are not expected once the epiphyseal line is closed, and the drug must be administered before puberty, at which the epiphyseal line is closed, to achieve its efficacy. Efficacy will be evaluated in a Phase 2 study. The growth rate of patients with ACH between 5 and 11 years of age is 4 cm per year regardless of gender and age (based on a nationwide survey of the height of children with ACH), which is the same patient population for which efficacy can be evaluated in a Phase 2 study by comparing the growth curve. The population was the same as that for which efficacy could be evaluated in the Phase 2 study by comparing growth curves. The target population was selected to accumulate data on drug concentration trends and kinetics in affected children to estimate the optimal clinical dosage and administration. 3. The patients were set as those for whom the clinical trial could be conducted in accordance with the study protocol.   (4), (5) In principle, consent should be obtained from the subject, but considering the subject's age, consent from only a surrogate is also acceptable for clinical trial participation.  (6) The setting was established because the investigational drug is a tablet for oral use. |
| **Exclusion Criteria.**  Patients who meet any of the following criteria should be excluded:   1. Patients who have taken drugs containing meclizine hydrochloride within 28 days prior to treatment initiation 2. Patients who have undergone bone-lengthening surgery within 28 days prior to treatment initiation or are scheduled to undergo bone-lengthening surgery during the study period 3. Patients with serious complications 4. Patients weighing less than 11 kg 5. Patients with symptoms of dysuria 6. Patients diagnosed with glaucoma 7. Patients with an allergy to meclizine hydrochloride 8. Patients who regularly take oral medications containing cold medicine, antipyretic analgesics, sedatives, antitussives, expectorants, and antihistamines 9. Other patients whom the investigator or sub-investigator deems inappropriate for participation in this clinical trial.   [Rationale]   1. Established to properly evaluate the safety and pharmacokinetics of the drug.   (ii) ~ (ix) Set to ensure the safety of subjects. |
| **Dosage and administration**  One or two tablets are administered orally once daily for 14 days; each tablet contains 12.5 mg of meclizine hydrochloride.  [Rationale for setting the dosage].  Pharmacokinetic analysis of meclizine hydrochloride after administration of 25 mg once or twice daily showed that the exposure dose was higher than the optimal dose; hence, half the original dose (12.5 mg) was established. After the safety is confirmed, the 25 mg dose group will be initiated |
| **Assessment Items**  Primary endpoints  Safety: Event name, severity, and frequency of occurrence for all adverse events  Pharmacokinetics: Meclizine plasma drug concentration parameters  Cmax (up to 24 h after administration)  Tmax (up to 24 h after administration)  (③) t_1/2_ (up to 24 h after administration)  AUC (up to 24 h after administration) |
| **Rationale for setting the primary endpoints:**  Given that there is no record of continuous meclizine hydrochloride administration to pediatric patients at the current scientific level and the main purpose of the study was to evaluate its safety and pharmacokinetics, we established safety and pharmacokinetics as primary endpoints of the present study |
| **Statistical analysis method:**  1. Primary endpoints  Safety: Adverse events between the first dose and 21 days  Adverse events will be classified and tabulated according to CTCAE v5.0/ MedDRA/J v23.1.  The incidence and 95% confidence interval of all adverse events observed from the first dose to the end of the evaluation will be calculated. The 95% confidence intervals will be calculated using the Clopper-Pearson method. The incidence of adverse events will be calculated by organ category, basic term, severity, and relationship to the study drug. Serious adverse events are calculated similarly.  Pharmacokinetics: Based on plasma meclizine concentrations up to 6 h after the first dose and up to 24 h after the 14th dose, the following values will be calculated:  Cmax  Tmax  Based on plasma meclizine levels up to 24 h after 14 doses  (③) t_1/2_ (up to 24 h after administration)  Based on plasma meclizine levels up to 6 h after the first dose and up to 24 h after the 14th dose  4) AUC up to 6 h (after the first and 14th doses) and up to 24 h (after the 14th dose) |
| **Concomitant medications/anti-concurrent therapies in clinical trials:**   1. Drug use prohibited in combination with other drugs   The following concomitant medications are prohibited for a period of 28 days prior to treatment initiation until the end of the evaluation period.  (i) Drugs shown to suppress fibroblast growth factor receptor 3 (FGFR3) signaling (e.g., C-type natriuretic peptide (CNP) analogs not approved in Japan and statins)  (ii) Drugs such as antiasthmatic drugs containing meclizine hydrochloride  The following concomitant medications are prohibited for 24 h prior to initiating the study drug administration and for 24 h after the end of the study drug administration.  Cold remedies, antipyretic analgesics, sedatives, antitussive expectorants, oral drugs containing antihistamines (e.g., rhinitis remedies and anti-allergy medications)   1. Concomitant therapy   The following treatments are prohibited from the period after consent is obtained until the end of the evaluation period  (i) Any growth hormone therapy administered at the time of patient consent cannot be discontinued or altered until the end of the evaluation period.  Treatment with bone-lengthening medications is prohibited. |
| **Assessment Schedule:**  **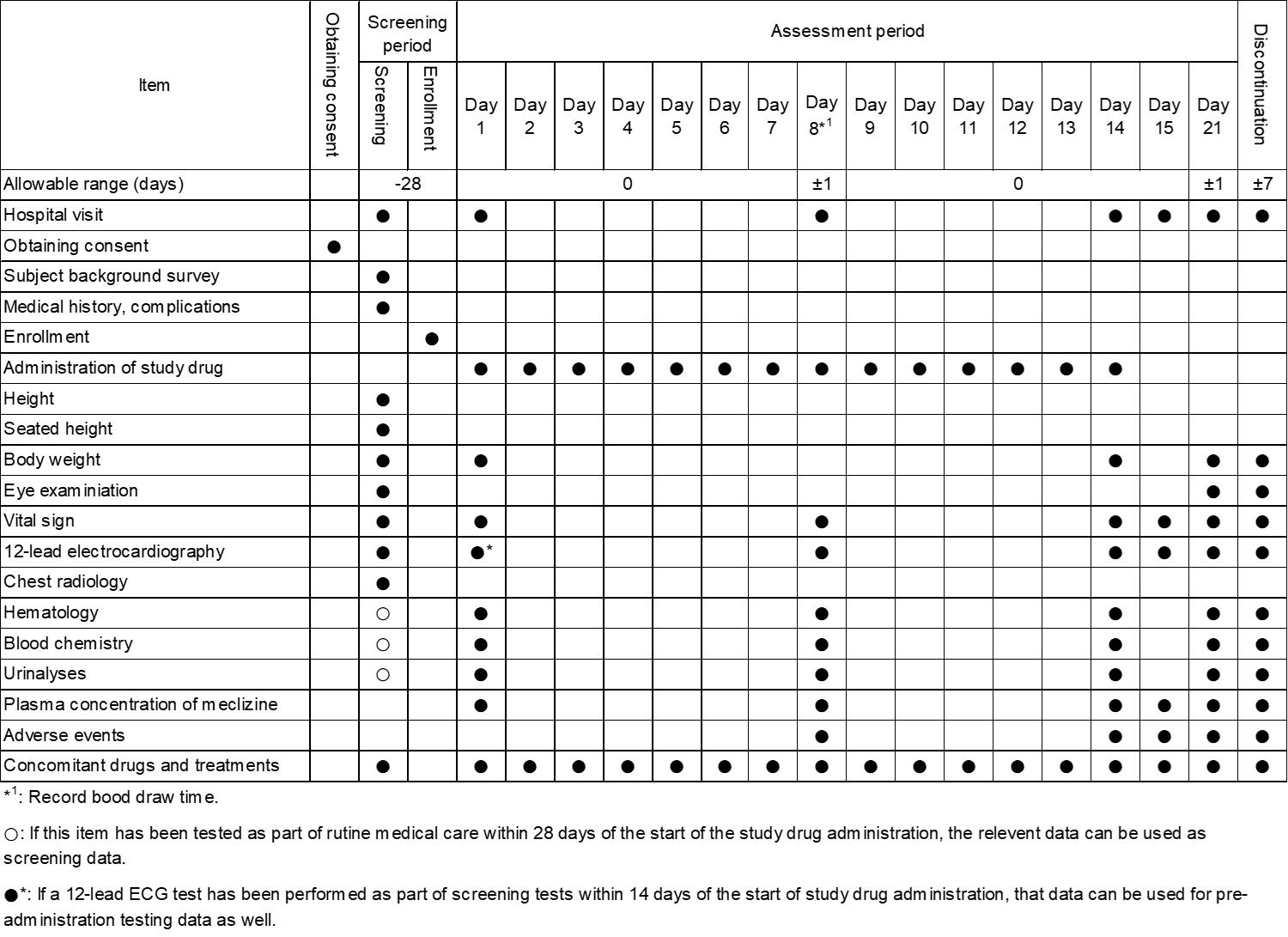**  Pharmacokinetic blood collection chart    **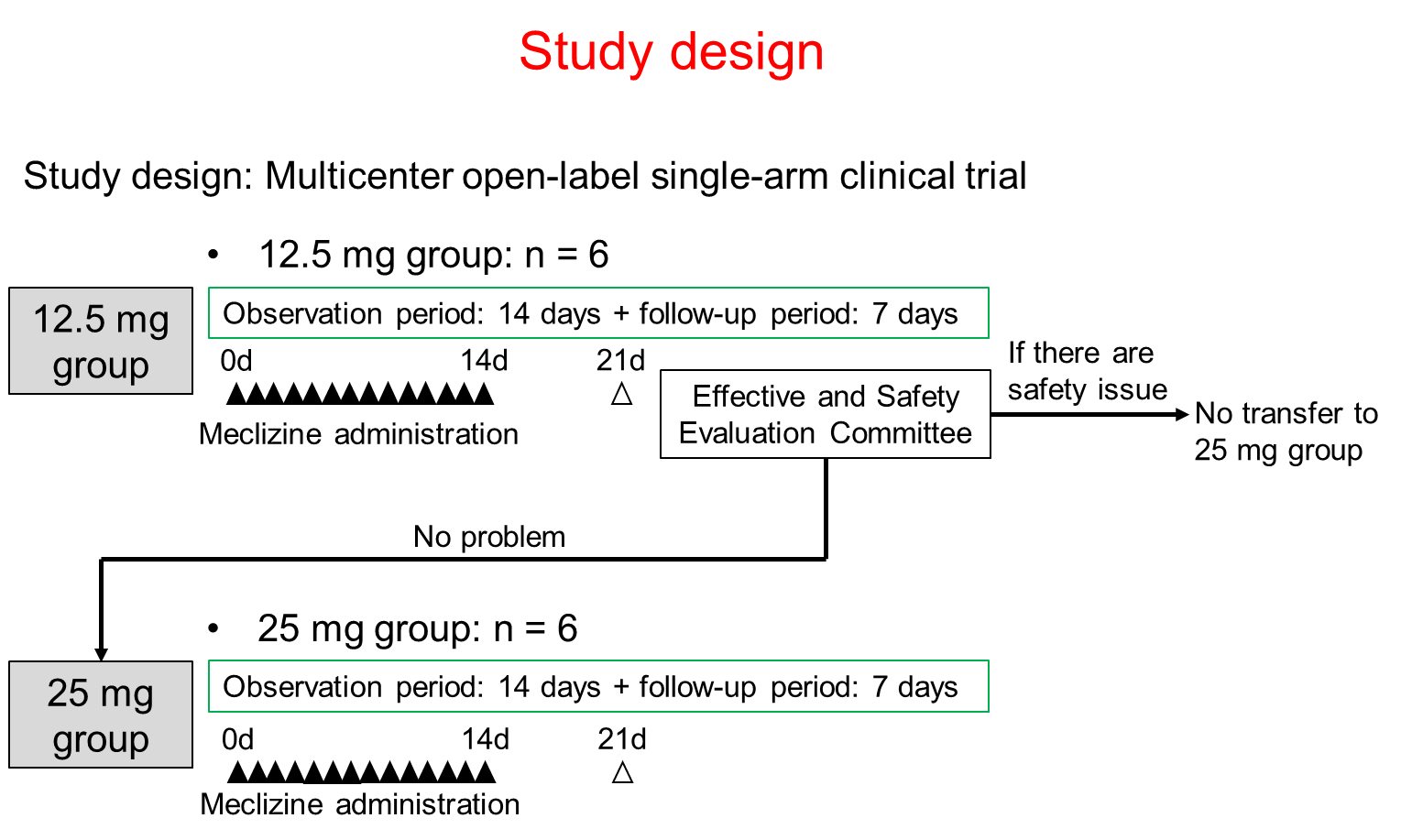**  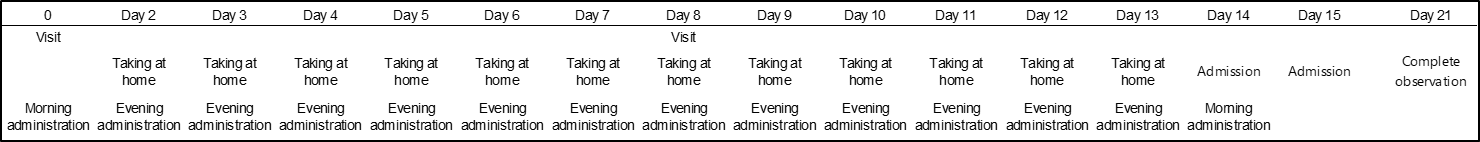 |

# 1. Purpose of the clinical trial

To evaluate the pharmacokinetics and safety of meclizine hydrochloride after repeated oral administration for 14 days in pediatric patients with achondroplasia.

## 1.1. Test design

Multicenter, open-label, uncontrolled trial

## 1.2. Primary endpoints

Safety: Adverse event name, severity, and frequency of occurrence during the first 21 days after the first dose.

Pharmacokinetics: Meclizine plasma drug concentration parameters

Cmax (after the first and 14th doses)

Tmax (after the first and 14th doses)

(③) t_1/2_ (after 14 doses)

AUC (after the first and 14th doses)

# 2. Background and rationale for the clinical trial

## 2.1. About achondroplasia

Achondroplasia (ACH) is the most common bone system disorder characterized by short stature with shortened limbs and is caused by excessive fibroblast growth factor receptor 3 (FGFR3) activation owing to gain-of-function mutations in FGFR3, a factor known to inhibit bone growth. In addition to short stature, severe complications such as spinal canal stenosis and foramen magnum occipitalis stenosis may also occur. In particular, the impaired long-axis growth of limb bones results in a final adult height of 120–130 cm, which is a substantial obstacle to daily life. Currently, no treatment is available to suppress the excessive activation of FGFR3 signaling in ACH. The symptomatic treatment for short stature involves medical growth hormone therapy and surgical bone lengthening, although the former is ineffective and the latter is highly invasive. Recently, several small molecule compounds that inhibit FGFR3 signaling have been identified, but the safety of these compounds has not been established in non-clinical studies1）2）. C-type natriuretic peptide (CNP), a bioactive compound, was shown to be effective in an animal model of ACH; however, its half-life is markedly short, only a few minutes 3） . Additionally, a CNP analog (BMN111) has been developed in the U.S. and is currently in clinical trials; however, this is an injectable drug, and its long-term administration in children can be burdensome4）. A screening system using induced pluripotent stem (iPS) cells has demonstrated the inhibitory effect of statins on FGFR3 signaling, but the optimal dosage and duration of use, as well as side effects, remain unexplored5）. The effects of statins on the daily lives of patients remain unknown. Treatment of ACH, which is well-known to considerably impact daily life, must be initiated during childhood; hence, developing a less invasive and more precise treatment strategy for ACH is necessary.

On June 25, 2020, the Pharmaceuticals and Medical Devices Agency (PMDA) provided in-person advice, and the following advice was received.

Based on the available non-clinical study results, it is feasible to initiate Phase I (2-week continuous dosing) and Phase I (continuation) studies, as proposed by the consultant, considering the sufficient data from non-clinical studies. Regarding the dosage and administration of meclizine in a Phase I (2-week continuous) study, considering safety, we believe it is feasible to initiate the study at 12.5 mg once daily, followed by 25 mg once daily based on safety information derived from six patients treated with 12.5 mg once daily.

Based on the above advice from PMDA, we planned a study to investigate the pharmacokinetics and safety of meclizine hydrochloride 12.5 mg tablets once or twice daily for 14 days in pediatric patients with ACH.

In addition, we decided not to conduct the Phase I (continuation) study presented to PMDA.

## 2.3. About investigational new drugs

Meclizine hydrochloride is a prescription drug approved in the United States on February 14, 1957, to relieve motion sickness symptoms. In Japan, it has been marketed as an over-the-counter (OTC) drug (Class II drug) since 1981 and is indicated for patients aged ≥3 years. Meclizine hydrochloride has been used in developed countries for more than 60 years, and during this period, no regulatory authority in Japan or the United States has indicated the need for regulation in terms of safety. In addition, no adverse drug reactions were documented on administering 50 mg/day (two 25 mg doses) repeatedly for three months to six children with learning disabilities, aged between 9 years 9 months and 14 years 8 months6）.

Meclizine hydrochloride has long been used as a prescription or OTC drug in Japan and overseas; however, there is a paucity of non-clinical safety study information for undertaking clinical trials. Therefore, with the advice of the PMDA Pharmaceutical Affairs Strategy Consultation, single-dose toxicokinetic studies and preliminary 1-week and 2-week repeated-dose toxicity studies were conducted in rats and dogs. Considering two-week repeated-dose studies, deaths were observed in the 150 mg/kg rat group, whereas hepatocyte vacuoles (fat in the 150 mg/kg group), vacuolation of the spleen, vacuolation of the proximal tubular epithelium of the kidney, and increased fatty droplets in adrenocortical cells were noted in the 50 and 150 mg/kg groups, suggesting the involvement of the lipid system. Other effects include atrophy of the mammary gland in males and hyperplasia of the mammary gland in females (150 mg/kg), swelling of ocular lens fibers in males (150 mg/kg) and females (50 and 150 mg/kg), the appearance of atypical remnant bodies and sperm cell remains in the seminiferous tubules of the testis, and increased cell remnants in the epididymal tubules of the testis (150 mg/kg). Accordingly, 15 mg/kg was established as the non-toxic dose for both males and females. In dogs, liver swelling was observed in all males in the 300 mg/kg dose group and all females in the 100 mg/kg dose group, and changes in blood biochemical parameters revealed that the non-toxic dose was 300 mg/kg in males and 100 mg/kg in females, indicating species-related differences. The results of protein binding rate, hepatic metabolism-specific clearance, and metabolite profiling studies indicated the presence of metabolites (dihydroxides) in rats, which was not observed in humans and dogs. Furthermore, genotoxicity and safety assessments were conducted to confirm safety, and by March 2017, “Guidance on Conducting Non-clinical Safety Studies for Clinical Trials and Marketing Authorization Applications for Pharmaceuticals” (Pharmaceutical and Food Safety Agency Issue No. 0219-4 dated February 19, 2010, ICH M3 [R2]) was established. Non-clinical safety studies, which are required to submit a notification of clinical trial for Phase I clinical trials of a new active ingredient based on the "Guidance on Conducting Non-clinical Safety Studies for Clinical Trials and Marketing Authorization Applications for Drugs" (ICH M3 (R2) Guidance, Pharmaceutical Affairs Agency, dated February 19, 2010), were completed.

Furthermore, in a 13-week repeated oral toxicity study in beagle dogs, increased liver weight, granular changes in hepatocytes, and high alkaline phosphatase (ALP) levels were observed in the low-dose group (100 mg/kg), and elevated liver weight, hepatocyte vacuolation, granular changes and hepatocyte hypertrophy, and high levels of alanine transaminase (ALT) and ALP were observed in the high-dose group (300 mg/kg). In addition, low glucose levels and decreased body weight were documented. The non-toxic dose (NOAEL) was estimated to be ˂100 mg/kg/day for both males and females.

Both male and female juvenile rats exhibited hepatocyte vacuolation, bulging of the lens of the eye, and foam cell infiltration in the alveoli. Male juvenile rats presented with atypical residues in the testis, degeneration of giant cells and spermatocytes, and cellular residues in the epididymis, while females exhibited the absence of the corpus luteum and persistence of estrus, but a recovery trend was observed after the withdrawal period.

## 2.4. Efficacy of the investigational drug in ACH

In 2013, Kito, Matsushita, Ohno, and colleagues at Nagoya University Graduate School of Medicine performed an exhaustive drug screening of 1,186 FDA-approved drugs in ACH model cells, revealing that meclizine HCl can inhibit Erk phosphorylation in the mitogen-activated protein kinase (MAPK) pathway downstream of FGFR3 in various cartilage cells in various cartilage cells. In addition, meclizine hydrochloride could induce bone elongation in the tibial organ culture system of embryonic mice. Orally administered meclizine hydrochloride by mixed feeding at a dose equivalent to the plasma concentration range to relieve motion sickness could rescue bone elongation in the ACH model mice. In a single-dose pharmacokinetic (PK) study, we confirmed that a 2 mg/kg dose in mice induced a blood concentration at the same level as the clinically used dose (25 mg) of meclizine hydrochloride used to relieve motion sickness. Therefore, when the drug was orally administered quantitatively, administration of 1 and 2 mg/kg/day meclizine hydrochloride twice daily could promote bone length in model mice in a concentration-dependent manner. Histologically, the width of the hypertrophic chondrocyte layer in the growing chondrocyte zone was also increased. In addition, treatment with meclizine hydrochloride could increase bone mass in the long bones and vertebrae and improve bone quality at the metaphyseal end, confirming the *in vivo* inhibitory effect of FGFR3.

## 2.5. Conduct of this clinical trial

From July 2018 to November 2018, a single-dose Phase 1 study was completed in 12 children with ACH 7）. Administration of 25 mg meclizine hydrochloride per dose to children with ACH induced no safety concerns, and PK results revealed no accumulation-related concerns. Considering that non-clinical studies have established the potential efficacy of the investigational drug in patients and considering the mechanism of action, we believe that meclizine hydrochloride could be highly effective, warranting clinical trials to confirm the safety and PK of the drug after repeated administration in patients.

# 3. Overview of the investigational drug

## 3.1. Investigational drug

1) Identification symbol of the investigational drug: MECLIZINE

2) Ingredients: [Generic name] Meclizine hydrochloride

Meclizine hydrochloride

International name: Meclizine (Meclozine) HCl

Molecular formula: C25H27Cl N2,2HCl・H_2_O

Molecular weight: 481.89

Structural formula:
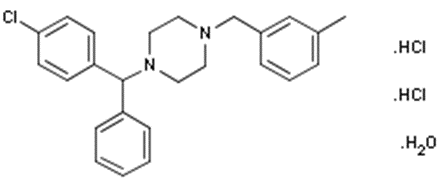


3) Dosage form and content: A new formulation containing 12.5 mg of meclizine hydrochloride manufactured by Meiji Pharmaceutical will be used based on the formulation and manufacturing method of Solasidone, approved as an OTC drug (Class II drug).

(4) Investigational drug provider: Nagoya University Hospital 65 Tsurumai-cho, Showa-ku, Nagoya, Aichi, Japan

## 3.2 Formulation composition of the investigational drug

Solasidone clinical trials (Meiji Pharmaceuticals):

One tablet contains 12.5 mg of meclizine hydrochloride

(Additives) D-mannitol, cellulose, sodium carmellose, hardened oil, magnesium stearate, and l-menthol

Expiration date: 36 months after manufacture at room temperature (tentative)

## 3.3 Packaging and labeling

The investigational drug label shall indicate that it is for a clinical trial, the identification code, generic name, and the name, affiliation, title, and address of the coordinating physician.

Packaging form: Tablets are packaged in PTP (Press Through Package) sheets

The PTP sheet is sealed in a paper box (casing).

Labeling: The label to be affixed to the packet shall include the following

(1) The name of the product indicating that the product is for a clinical trial.

(ii) Identification Code

(3) Serial No. (Lot.) Serial number (Lot.)

(4) Generic name

5) Storage and handling precautions

⑥ Terms of validity

(vii) Affiliation, name, title, and address of the coordinating investigator

Note: The position title was altered to Hospital Lecturer effective July 1, 2021, but will be read as such.

| **For Clinical Trials** | **Safety and pharmacokinetics of meclizine hydrochloride after 2 consecutive weeks of administration to pediatric patients with achondroplasia** |
| --- | --- |
|  | **Not to be used for any purpose other than this clinical trial** |
| Identification Symbols | MECLIZINE |
| Serial No. | *** |
| Generic name | meclizine hydrochloride (HCl) |
| Precautions for storage and handling: 1 | Store away from direct sunlight and moisture |
| Period of validity: 1 year | July 2023 (tentative) |
| Clinical Trial Coordinating Physician: | Nagoya University Hospital, Department of Orthopaedic Surgery  Masaki Matsushita, Project Assistant Professor  65, Tsurumai-cho, Showa-ku, Nagoya, Aichi, Japan |

## 3.4 Handling, storage, and control of investigational drug

The investigation coordinator shall purchase Solasidone Clinical Trial, manufactured for the clinical trial, and change the labeling to establish it as an investigational drug. The investigational drug manager shall store and manage the investigational drug in a cool, dry place, away from direct sunlight, in accordance with the "Protocol for Management of Investigational Drugs" prepared by the investigational coordinator.

　Storage temperature: room temperature

# 4. Research plan and design

## 4.1. Type and design of the clinical trial

Phase 1, multicenter, open-label, uncontrolled study

## 4.2 Outline of the clinical Trial

The present clinical trial will examine the safety and collect PK data in pediatric patients with ACH. Accordingly, the objective of the present study is to obtain data from plasma drug concentrations of meclizine administered once daily for 14 days to provide a basis for initiating a Phase 2 long-term repeated-dose study, as well as to provide sufficient safety data. Given that the drug concentration was the index, blinding was deemed unnecessary.

# 5. Eligible patients and eligibility criteria

After obtaining consent, patients who meet the selection criteria (1) to (6) in 5.1. and who do not conflict with exclusion criteria (1) to (8) in 5.2. at the time of enrollment will be included in the study.

## 5.1. Inclusion criteria

1. Patients with a confirmed diagnosis of ACH that meets definite or probable criteria established by the Ministry of Health, Labour and Welfare (MHLW) Intractable Disease Study Group at least one year prior to obtaining consent.
2. Patients aged 5 to 11 years at the time consent
3. Patients who can be admitted to the hospital on the last day of administration and are expected to complete the study
4. Patients with written consent from a surrogate
5. Patients ≥7 years of age with written consent from the subject
6. Patients who can take tablets

[Rationale]

(i) Established as the patient population to be treated.

ii) The pharmacological effects of the test drug are not expected after the closure of the epiphyseal line, and the drug must be administered before puberty, when the epiphyseal line is closed, to achieve a pharmacological effect. Efficacy will be evaluated in a Phase 2 study. The growth rate of patients with ACH, ranging between 5 and 11 years of age, is 4 cm annually regardless of gender and age (based on a nationwide survey of the height of children with ACH), which is the same patient population for which efficacy can be evaluated in a Phase 2 study by comparing growth curves. The target population was selected because it is necessary to obtain data on drug concentration trends and kinetics in affected children to estimate the optimal clinical dosage and administration.

(iii) Patients were set as those in whom the clinical trial could be conducted in accordance with the study protocol.

(iv) (v) In principle, consent should be obtained from the subject, but considering the subject's age, consent from only a surrogate is also acceptable for clinical trial participation.

(vi) The investigational drug is a tablet for oral use.

## 5.2. Exclusion criteria

(i) Patients who have taken drugs containing meclizine hydrochloride within 28 days prior to treatment initiation

(ii) Patients who have undergone bone-lengthening surgery within 28 days prior to treatment initiation

or are scheduled to undergo bone-lengthening surgery during the study period

(iii) Patients with serious complications

(iv) Patients weighing less than 11 kg

(v) Patients with symptoms of dysuria

(vi) Patients diagnosed with glaucoma

(vii) Patients with an allergy to meclizine hydrochloride

(viii) Patients who regularly take oral medications, including cold remedies, antipyretic analgesics, sedatives, antitussives, expectorants, and antihistamines

(ix) Other patients whom the investigator or sub-investigator deems inappropriate for clinical trial participation.

[Rationale]

(i) 　Established to precisely evaluate drug safety and PK.

(ii) ~ (ix) Set to ensure the safety of subjects.

# 6 Registration

## 6.1. Registration procedure

Enrollment will be conducted according to the following procedures: once the target number of patients in both the 12.5 mg and 25 mg dose groups has been reached, no further patients will be enrolled in the cohort that has been reached.

After obtaining written consent from subjects (and respective surrogates) deemed potentially eligible for this study and conducting screening tests, the investigator or sub-investigator will enter all necessary items for registration and eligibility verification if the subject meets established registration criteria. At the registration center, eligibility is determined based on the information entered by the investigator or sub-investigator. If there is any doubt regarding the input information, the registration center will immediately confirm it with the investigator or sub-investigator.

The registration center will contact the investigator or sub-investigator if the registration criteria are met.

If the registration criteria are unmet, the investigator or sub-investigator shall record the reason in the source document.

## 6.2. Precautions for registration

The present study is a Phase I study, and if the subject and his/her surrogate do not understand that meclizine hydrochloride is not expected to be effective and that adverse effects and other disadvantages may occur, enrollment of the subject is unacceptable.

If any of the fields required for registration are incomplete, the registration will not be accepted until all are satisfied.

Once a subject has been enrolled, his/her enrollment will not be canceled.

If an incorrect or duplicate registration is detected, the registration center shall be notified immediately.

In the event of duplicate registrations, the earlier registration shall take precedence.

## 6.3. If the subject is determined ineligible for clinical trial participation

If the investigator or sub-investigator determines that a subject is ineligible, the investigator or sub-investigator will provide appropriate treatment as necessary. A case report form will not be prepared for subjects who are not enrolled after obtaining consent.

# 7. Method of the clinical trial

## 7.1. Dosage and administration

**7.1.1. Administration of the investigational drug**

The dosage and administration of the investigational drug are as follows

... One or two tablets of the investigational drug should be taken once daily, 1 h after dinner intake. The 1st and 14th doses should be administered at the clinic and taken 1 h after breakfast.

Meal intake should be completed 1 h before dose administration. The study drug should be taken without chewing with a fixed amount of water (~150 mL). The tolerable dose range is +/- 30 min.

However, water intake is not restricted throughout the day.

Record the time of completing meal intake and administration of the study drug in the source document.

[Rationale]

In a single-dose assessment of meclizine hydrochloride, pediatric patients with ACH exhibited higher plasma meclizine levels at the 25 mg dose than adults, with higher exposure observed on administering after feeding than when administered with fasting, along with a large variability in exposure. Therefore, the basic dose was set at 12.5 mg per dose. After confirming the safety information in the 12.5 mg group, the administration will be initiated in the 25 mg group. In addition, given that hospitalization was not required during the entire study period, it was necessary to consider the effects of meclizine hydrochloride on sleep induction, one of the known pharmacological effects, and it was considered appropriate to administer the drug at night before falling asleep.

Based on the above evidence, we deemed it appropriate to administer meclizine hydrochloride 12.5 mg to pediatric patients with ACH (chondrodysplasia) as one or two tablets once daily after dinner for 14 days. After the 1st and 14th doses, blood samples will be collected multiple times for PK measurements; hence, the drug should be taken at a medical institution and administered in the morning. The 14th dose should be administered after confirming that at least 8.5 h, corresponding to the plasma half-life of the investigational drug, have passed since the previous nightly administration.

## 7.2. Study duration for each subject

The investigational period for an individual subject is defined as the period from the time of consent to the time of discontinuation or last observation (7 days after the last dose).

## Use of investigational drug after completion of clinical trial

The investigational drug will not be provided to subjects who have completed the clinical trial or who have discontinued clinical trial participation.

## Regulations regarding concomitant medications and concomitant therapies

**7.4.1. Concomitant use of prohibited drugs and therapies**

The following concomitant medications are prohibited from 28 days prior to initiating study drug administration until the end of the evaluation period.

(1) Drugs that are known to inhibit FGFR3 signaling (e.g., CNP analogs and statins not approved in Japan)

(2) Drugs containing meclizine hydrochloride

The following concomitant medications are prohibited 24 h prior to initiating study drug administration and for 24 h after the end of the study drug administration.

Cold remedies, antipyretic analgesics, sedatives, antitussives, expectorants, oral antihistamines

(e.g., rhinitis and allergy medications)

The following treatments are prohibited during the period from the time consent is obtained until the end of the evaluation period

4) Subjects receiving growth hormone therapy at the time of consent must not discontinue or alter therapy before the end of the evaluation period.

(5) Bone-lengthening therapy is prohibited.

The clinical trial will be terminated if these drugs or therapies are administered to treat a subject.

[Rationale]

(1) (2) Established to prevent excessive suppression of FGFR3 signaling.

(2) Established for appropriate PK evaluation.

(3) In accordance with the Solasidone package insert instructions.

(4) The use of growth hormone agents is not prohibited because no evaluation of efficacy will be conducted. To accurately observe and evaluate adverse events, discontinuation or modification of treatment during the study period is prohibited.

(5) Prohibited during the clinical trial period to ensure accurate observation and evaluation of adverse events.

**7.4.2. Record of concomitant medications/adjunctive therapy**

The investigator, sub-investigator, or collaborator shall record the following information regarding concomitantly used drugs during the period from obtaining consent to the end of the evaluation period. However, drugs used to dissolve injectable drugs, rehydration solutions, secure the blood collection route, and disinfectants do not need to be recorded.

Concomitant medications: drug name (trade name), duration of administration, dosage, and purpose of use

For concomitant therapy, the name, duration, and purpose of therapy should be recorded for any concomitant therapy used from the start of therapy to the end of the evaluation period, from the time of consent to the end of the evaluation period.

# 8. Evaluation items

## 8.1. Primary endpoints

Safety for 21 days after the first dose

The following information is obtained by interview, visual inspection, palpation, and clinical examination. The name, severity, and incidence of all adverse events from the first dose to the end of the evaluation

- PK: Meclizine plasma drug concentration parameters to be calculated are as follows (Plasma drug concentrations before and after administration of meclizine hydrochloride will be measured in all patients)

(1) Cmax (after the 1st and 14th doses)

(2) Tmax (after the 1st and 14th doses)

(3) t_1/2_ (after 14 doses)

AUC (after the 1st and 14th doses)

[Rationale]

Note that there is no prior record of continuous meclizine hydrochloride administration among pediatric patients at the current scientific level, and the main objective of the present study was to evaluate the safety and PK of meclizine hydrochloride; hence, safety and pharmacokinetics were established as the primary endpoints of the study. The t_1/2_ of meclizine in pediatric patients with ACH is 8.5 h, with ˃99.999% of the drug eliminated from the body after 7 days of the last dose, corresponding to a half-life of 17 days; hence, the evaluation period was considered reasonable.

# Observation and inspection items and timing

**<Table 1: Evaluation Schedule>**


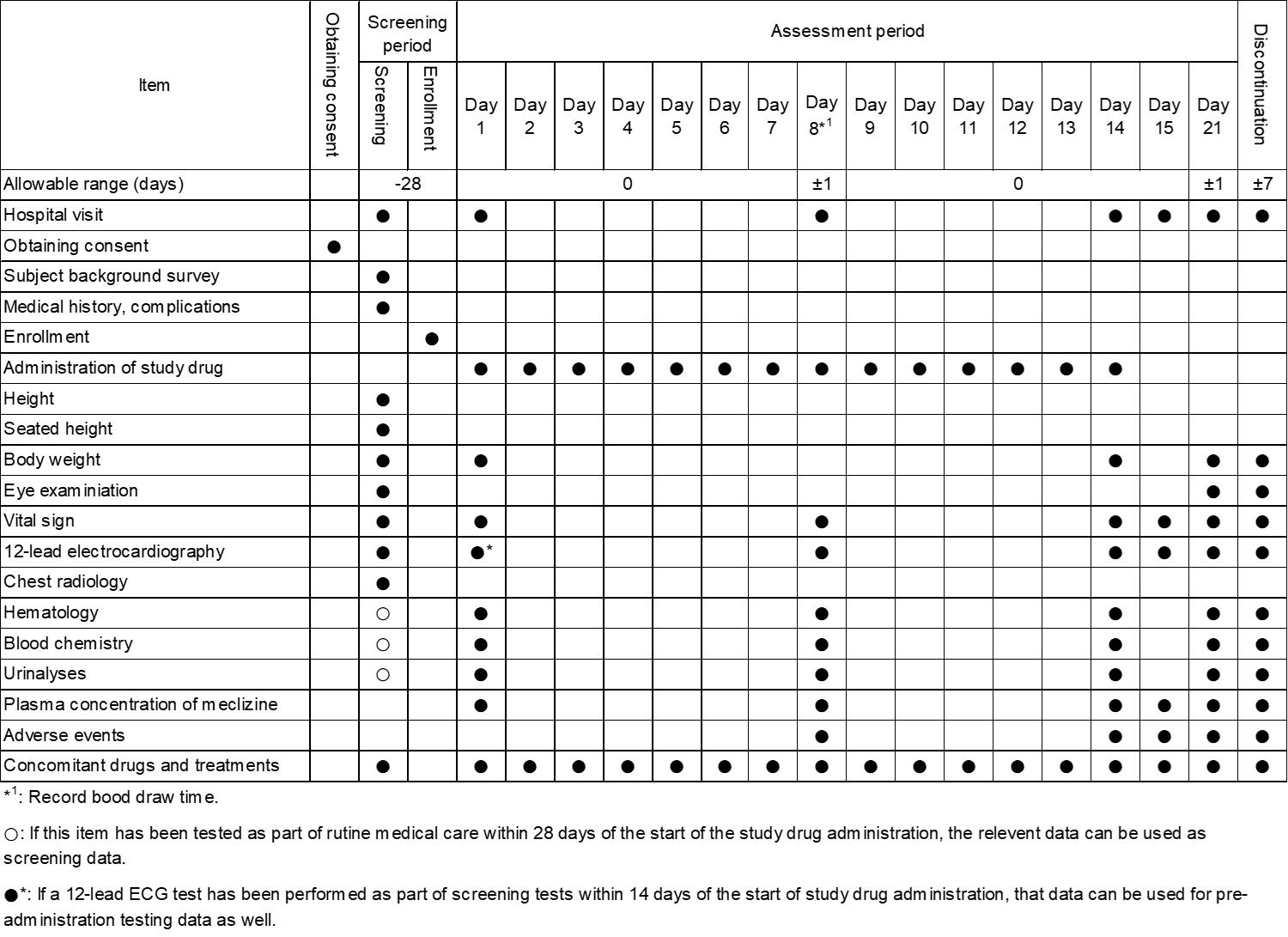


<Table 2 Blood collection schedule for pharmacokinetic measurements>


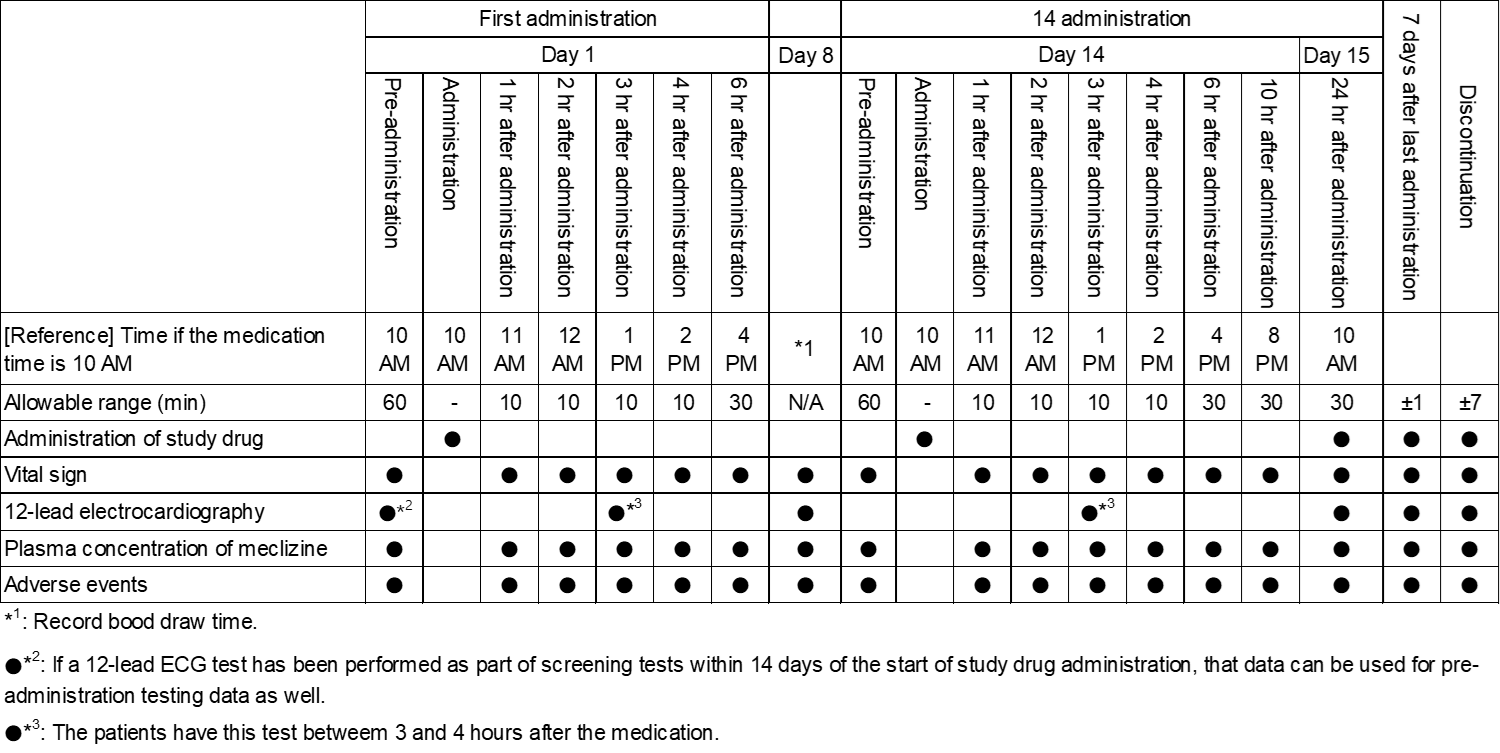


## 9.1. Observation and examination items

**9.1.1. Screening (after obtaining consent - registration)**

The screening period will last no longer than 28 days after consent is obtained, and the following tests and observations will be performed during the screening period.

1. Subject background: gender and date of birth
2. Height
3. Sitting height
4. Body weight
5. Ophthalmologic examination: a slit-lamp examination will be performed
6. Patient history and complications
7. Vital signs (blood pressure, pulse rate, temperature): Measured in the supine position
8. 12-Lead electrocardiogram (ECG)
9. Chest radiography (frontal view)
10. Hematologic examination: red blood cell count, white blood cell count, platelet count, hemoglobin level, and hematocrit level
11. Blood biochemical tests: total protein (TP), albumin (Alb), urea nitrogen (BUN), creatinine (Cre), C-reactive protein (CRP), sodium (Na), potassium (K), chloride (Cl), ALT, aspartate aminotransferase (AST), and triglycerides (TG)
12. Urinalysis: pH, protein, glucose, urobilinogen, and occult blood
13. Confirmation of concomitant medications and therapies

For "(10) Hematologic tests," "(11) Blood biochemical tests," and "(12) Urinalysis," if the subject consents to the use of such data as existing data, if tested as routine examination within 28 days prior to the start of treatment, these data will be used as data at screening.

Regarding "(6) History of illness and complications," "History of illness" refers to illnesses that have been cured from one year prior to obtaining consent until the start of treatment, and "Complications" refers to illnesses that are complicated on the date of initiating treatment.

**9.1.2.**  **Date of study drug administration - Day 1 (Pre-administration items)**

1. Body weight
2. Vital signs (blood pressure, pulse rate, and temperature): measured in the supine position
3. 12-Lead ECG
4. Hematologic examination: red blood cell count, white blood cell count, platelet count, hemoglobin level, and hematocrit level
5. Blood biochemical tests: TP, Alb, BUN, Cre, CRP, Na, K, Cl, ALT, AST, and TG
6. Urinalysis: pH, protein, glucose, urobilinogen, and occult blood
7. PK measurements
8. Confirmation of concomitant medications and therapies

If the screening test for "(3) 12-lead ECG" is performed within 14 days prior to the date of administration of the investigational drug, the data from the screening test will be used as the pre-dose data.

For the procedures for collecting, handling, and transporting samples for PK evaluation, a separate procedure manual will be used. In addition, the time of blood sampling for PK measurement will be recorded in the source document from "9.1.3. Start date of study drug administration" to "9.1.8 7 days after the end of study drug administration - Day 21 (at the end of the evaluation period)." Herein, “9.1.10. Allowable time range” will be used for the allowable range of blood sampling and ECG measurement time.

**9.1.3. Date of start of study drug administration (administration and 1, 2, 3, 4, and 6 h later)**

Dosage start

1. Vital signs (blood pressure, pulse rate, and temperature): measured in the supine position
2. PK measurements
3. Adverse events
4. Confirmation of concomitant medications and therapies
5. 12-lead ECG: 3 h after administration only

**9.1.4. Day 8 of study drug administration - Day 8 (visit between 10:00 and 15:00)**

1. Vital signs (blood pressure, pulse rate, and temperature): measured in the supine position
2. 12-lead ECG
3. Hematologic examination: red blood cell count, white blood cell count, platelet count, hemoglobin level, hematocrit level
4. Blood biochemical tests: TP, Alb, BUN, Cre, CRP, Na, K, Cl, ALT, AST, and TG
5. Urinalysis: pH, protein, glucose, urobilinogen, and occult blood
6. PK measurements
7. Adverse events
8. Confirmation of concomitant medications and therapies

**9.1.5. Two weeks later - Day 14 (pre-dose)**

1. Body weight
2. Vital signs (blood pressure, pulse rate, and temperature): measured in the supine position
3. Hematologic examination: red blood cell count, white blood cell count, platelet count, hemoglobin level, hematocrit level
4. Blood biochemical tests: TP, Alb, BUN, Cre, CRP, Na, K, Cl, ALT, AST, and TG
5. Urinalysis: pH, protein, glucose, urobilinogen, and occult blood
6. PK measurements
7. Adverse events
8. Confirmation of concomitant medications and therapies

**9.1.6. Day 14 (administration and 1, 2, 3, 4, 6, and 10 h later)**

Dosage

1. Vital signs (blood pressure, pulse rate, and temperature): measured in the supine position
2. PK measurements
3. Adverse events
4. Confirmation of concomitant medications and therapies
5. 12-lead ECG: 3 h after administration only

**9.1.7. Day 15 (24 h after administration)**

1. Vital signs (blood pressure, pulse rate, and temperature): measured in the supine position
2. 12-lead ECG
3. PK measurements
4. Adverse events
5. Confirmation of concomitant medications and therapies

**9.1.8. Seven days after completion of the investigational drug administration - Day 21 (at the end of the evaluation period)**

1. Body weight
2. Ophthalmologic examination: a slit-lamp examination will be performed
3. Vital signs (blood pressure, pulse rate, temperature): measured in the supine position
4. 12-lead ECG
5. Hematologic examination: red blood cell count, white blood cell count, platelet count, hemoglobin level, and hematocrit level
6. Blood biochemical tests: TP, Alb, BUN, Cre, CRP, Na, K, Cl, ALT, AST, and TG
7. Urinalysis: pH, protein, glucose, urobilinogen, and occult blood
8. PK measurements
9. Adverse events
10. Confirmation of concomitant medications and therapies

**9.1.9. In the event of cancelation**

Treatment at the time of discontinuation: The clinical trial will be discontinued under the circumstances described in "10. Criteria for discontinuation".

When discontinuing the investigational drug after administration, the following discontinuation tests should be performed within 7 days of the decision to discontinue.

1. Body weight
2. Ophthalmologic examination: a slit-lamp examination will be performed
3. Vital signs (blood pressure, pulse rate, and temperature): measured in the supine position
4. 12-lead ECG
5. Hematological examination: red blood cell count, white blood cell count, platelet count, hemoglobin level, and hematocrit level
6. Blood biochemical tests: TP, Alb, BUN, Cre, CRP, Na, K, Cl, ALT, AST, and TG
7. Urinalysis: pH, protein, glucose, urobilinogen, and occult blood
8. PK measurements
9. Adverse events
10. Confirmation of concomitant medications and therapies

**9.1.10. Time tolerance**

Blood collection for Pharmacokinetic measurements

Date of the first dose (before administration) : a 60-min interval should be maintained before the first dose.

Date of the first dose (1 h after administration): a 10-min interval should be maintained before and after the first dose.

Date of the first dose (2 h after administration): a 10-min interval should be maintained before and after the first dose.

Date of the first dose (3 h after administration): a 10-min interval should be maintained before and after the first dose.

Date of the first dose (4 h after administration): a 10-min interval should be maintained before and after the first dose.

Date of the first dose (6 h after administration): a 10-min interval should be before and after the first dose.

Day 8 of dosing: the day the patient will visit the hospital and the time of blood collection should be recorded.

Date of 14 doses (before dosing): a 60-min interval should be maintained before administration of the 14 doses.

Day of 14 doses (1 h after dosing): a 10-min interval should be maintained before and after the dose.

Day of 14 doses (2 h after dosing): a 10-min interval should be maintained before and after the dose.

Day of 14 doses (3 h after dosing): a 10-min interval should be maintained before and after the dose.

Day of 14 doses (4 h after dosing): a 10-min interval should be maintained before and after the dose.

Day of 14 doses (6 h after dosing): a 10-min interval should be maintained before and after the dose.

Day of 14 doses (10 h after dosing): a 30-min interval should be maintained before and after the dose.

Day 2 of 14 doses (24 h after dosing): a 30-min should be maintained interval before and after the dose.

One week after the completion of 14 doses, a 1-day interval should be maintained before and after the end of the 14 doses.

12-Lead ECG

The measurement should be performed between the blood collection 3 h after administration and blood collection 4 h after administration.

**9.1.11. Sample collection volume**

1) Clinical laboratories: To be stipulated by each investigational site as appropriate

2) PK measurements: Approximately 2 mL of whole blood

## 9.2. Observation and examination methods

**9.2.1. Confirmation of adverse events**

Confirm the occurrence of adverse events by interview, visual examination, palpation, ultrasonography, X-rays, and other clinical examinations.

**9.2.2. Checking vital signs and laboratory values**

Vital signs and laboratory values should be established at the time points specified in the evaluation schedule (and other times as needed).

# 10. Criteria for discontinuance

The investigator or sub-investigator may decide to administer the investigational drug to the subject or discontinue the subject's participation in the study throughout the duration of the study. Similarly, a subject may, at any time and for any reason, voluntarily request discontinuation of investigational drug administration or discontinuation of participation in the clinical trial.

The clinical trial will be discontinued if any of the following discontinuation criteria are met.

1. When the subject or surrogate freely withdraws consent
2. When the investigator or sub-investigator determines that it is difficult to continue the study owing to worsening of the underlying disease or complications or due to the occurrence of serious adverse events
3. When a serious deviation from the study protocol is found, such as GCP violations, selection criteria violations, or exclusion criteria violations
4. When it becomes impossible to comply with the clinical trial protocol
5. If the subject is found to be inappropriate for PK/accumulative or safety evaluation during the clinical trial period
6. Other instances in which the investigator or sub-investigator deems that clinical trial continuation is difficult

[Rationale].

(1)-(6) Established to ensure ethical conduct of the clinical trial.

# 11. Adverse events

## 11.1. Response to subjects in the event of adverse events and investigation and reporting of adverse events

When an adverse event is observed in a subject, the investigator or sub-investigator shall immediately take appropriate measures and inform the subject if medical treatment for the observed adverse event becomes necessary. Furthermore, the symptoms or diseases associated with the adverse events observed after initiating investigational drug administration, the details of other findings, the date of occurrence, the severity, if judged as a serious event, the reason for the judgment, the presence or absence of treatment and its details, the outcome and its judgment date, and if related to the administered investigational drug and causal relationship, the reason for such a relationship, if any, shall be recorded. However, in the case of a serious adverse event, the reason for the judgment should be recorded in the presence or absence of a causal relationship.

In principle, except in cases where the investigation is no longer possible due to the death of the subject or transfer of the subject to a different hospital, etc., follow-up should be conducted until normalization or recovery to a level that is not considered an adverse event. In the case of irreversible adverse events due to organic disorders (e.g., cerebral infarction or myocardial infarction), a follow-up investigation shall be conducted until the symptoms stabilize. However, for serious adverse events, if the investigator or sub-investigator judges that the patient has recovered to a level where it is not considered an adverse event and the organic disorder has stabilized as a symptom, the basis for that judgment shall be recorded.

If a serious adverse event occurs after initiating the investigational drug administration, regardless of whether or not there is a causal relationship with the administered investigational drug, the investigator or sub-investigator shall immediately take appropriate measures and actions. In addition, in accordance with the "Procedures for Handling Safety Information," the investigator shall promptly report the event to the head of the institution, the investigational coordinator, and the drug supplier. The investigator shall also report to the Minister of Health, Labour and Welfare, as necessary.

The investigator shall report the progress of the clinical trial to the head of the institution annually.

## 11.2 Definitions of adverse events, etc.

1) Adverse events

An adverse event is any disease or disorder or any sign of such disease or disorder (including abnormal changes in laboratory values) that occurs in a subject treated with the investigational drug, regardless of whether or not it is causally related to the investigational drug. In this study, any unfavorable medical event that occurs from the initiation of investigational drug administration to the end of the evaluation period/discontinuation of the study will be treated as an adverse event.

2) Classification of the degree of adverse events

Adverse events that occur in subjects will be classified and tabulated according to CTCAE v5.0/ MedDRA/J v23.1.

Grade refers to the severity of the adverse event; Grades 1–5 are defined in the CTCAE according to the principles in the table below, with a separate explanation of severity for each adverse event. In the present study, complications will be judged as adverse events if the grade worsens by one or more levels, according to CTCAE ver. 5.0. Abnormal laboratory values will be judged in the same manner.

**＜Table 2 Grade according to CTCAE ver. 5.0>**

| Grade | Explanation^a)、b)^ |
| --- | --- |
| Grade 1 | Mild disease; no symptoms or mild symptoms; clinical or laboratory findings only; no treatment required |
| Grade 2 | Moderate; requires minimal/local/noninvasive treatment; age-appropriate limitation in activities of daily living other than personal care^c)^ |
| Grade 3 | Severe or medically significant but not immediately life-threatening; requiring hospitalization or prolonged hospitalization; limitation of activities of daily living^d)^ |
| Grade 4 | Life-threatening; requires emergency treatment |
| Grade 5^e)^ | Death due to adverse events |

a) Semicolon (;) in the description means "or"

(b) Not all adverse events include all Grades, so some adverse events have less than 5 Grade options

c) Activities of daily living other than personal care include meal preparation, shopping for daily necessities and clothing, using the telephone, managing money, etc.

d) Activities of daily living implied the ability to bathe, dress/undress, eat, use the toilet, take medications, and not be bedridden

e) Not included as an option because Grade 5 (death) is not applicable for some adverse events

3) Classification of adverse event outcomes

Adverse event outcomes will be classified according to the following criteria

(1) Recovery

(2) Improvement

(3) Recovered but with sequelae

(4) Unrecovered

(5) Death

(6) Unknown

4) Causal evaluation of adverse events

The investigator or sub-investigator evaluates whether the investigational drug has a "reasonable possibility" of causing the adverse event. The evaluation shall be classified as follows, taking into account causes other than the investigational drug, such as the underlying disease, the natural history of the underlying disease, such as complications, concomitant therapy, and other risk factors, as well as the temporal relationship between the use of the investigational drug and the occurrence of the event.

1. Related
2. No relation

5) Predictability of adverse events

In this clinical trial, adverse events related to the investigational drug are defined as "unknown" if the occurrence of the event or the trend of occurrence, such as the number of occurrences, frequency, conditions of occurrence, etc., cannot be predicted from this Investigational New Drug Summary or the following, and "known" if they can be predicted. However, safety information reported to the Minister of Health, Labour and Welfare shall be handled as a separate volume of the Investigational New Drug Summary, and such events shall be treated as "known" as on the date of preparation of such report document.

6) Serious adverse events

A serious adverse event is defined as any of the following adverse events

(i) Death

(ii) Items that may lead to death

(iii) Requires hospitalization or prolonged hospitalization for treatment (however, hospitalization for the following reasons is not considered a serious adverse event)

- Planned hospitalization as specified in the clinical trial protocol

- Admission to the hospital: Hospitalization that was planned prior to initiating the clinical trial or scheduled to take place during the clinical trial

- Hospitalization for tests, etc. (without adverse events)

(iv) Permanent or serious disability or dysfunction

(v) Items that may lead to disability

(vi) Cases that are as serious, as listed in (i) through (v) above

(vii) Congenital diseases or anomalies, etc., in subsequent generations

## 11.3. Actions to be taken in case of serious adverse events

If a serious adverse event occurs in a subject after initiating investigational drug administration, the investigator coordinator, principal investigator, and the head of the institution shall perform the following in accordance with the "Procedures for Handling Safety Information".

(i) The investigators and sub-investigators shall immediately take necessary measures to ensure the safety of the subject, regardless of the existence of a causal relationship, and the investigators shall report the event to the head of the institution, the investigational coordinator and the study drug supplier. The investigator must report to the head of the institution and the study coordinating physician the circumstances of the occurrence of the event and the symptoms of the subject using the Serious Adverse Event Report within about 24 h of being made aware of the occurrence of the event, in principle. Information regarding the circumstances of the event occurrence and the respective symptoms of the subject should also be provided to the investigator.

ii. When additional information on such adverse events is obtained, the investigator shall provide an additional report to the head of the institution and the study coordinator as soon as possible by means of a report on serious adverse events and provide the information to the study sub-investigator.

(iii) The coordinating physician for the clinical trial shall report to the Minister of Health, Labour and Welfare when he/she judges that the case falls under the reporting subject stipulated in Article 273-2, paragraph 1 of the Enforcement Regulations of the Act on Quality, Efficacy and Safety Assurance of Pharmaceuticals and Medical Devices, etc., and that a report to the Minister of Health, Labour and Welfare is necessary.

## 11.4. Adverse events expected in this clinical trial

Predicted adverse events, etc.

Adverse event symptoms listed in the Solasidone package insert are as follows

Skin: Rash/redness and itching

Urology: dysuria

Other: dry mouth and drowsiness

In addition, the adverse event symptoms of the OTC drug Senpaa S (Taisho Pharmaceutical: currently discontinued) with the same active ingredient described in the study protocol of the single-dose Phase 1 trial “Safety and pharmacokinetics of 2-week continuous administration of meclizine hydrochloride in pediatric patients with achondroplasia” (CAMCR-009) are as follows.

Skin: rash/redness, itching

Psychoneurotic system: headache

Urinary system: dysuria

Other: facial burning, unusual glare, dry mouth, constipation, drowsiness, blurred vision

The appearance of these symptoms and the diseases associated with these symptoms shall be deemed "known".

## 11.5 Adverse event recording procedures

When recording adverse events, the investigator or sub-investigator should endeavor to use appropriate medical terminology and concepts and avoid the use of colloquialisms and abbreviations. Only one adverse event name should be entered in the event field of the record.

**11.5.1. Diagnosis and signs and symptoms**

For adverse events, the diagnosis (if identified) rather than individual signs and symptoms (e.g., record liver failure or hepatitis rather than jaundice or elevated transaminases) should be recorded. However, if a series of signs and symptoms cannot be medically identified as a single diagnosis or syndrome at the time of reporting, record each individual event separately. If the diagnosis is later confirmed, invalidate all previously reported adverse events based on signs and symptoms and replace them with a single adverse event report based on a single diagnosis, with the onset date of the first symptom of the final diagnosis as the onset date.

**11.5.2. Adverse events secondary to other events**

As a general rule, for adverse events that are secondary to other events (e.g., adverse events that occur consecutively or symptoms that follow in the clinical course), only the adverse event that was the primary cause should be listed, unless the secondary event is a severe or serious adverse event. However, medically significant adverse events that follow the causative event in a time interval should be recorded as independent events. Examples are listed below. If it is unclear whether the adverse events are related, record them separately.

. If the patient develops vomiting and mild dehydration that does not require treatment, only vomiting should be recorded.

. If vomiting results in severe dehydration, record both events separately.

. If renal failure is caused by severe gastrointestinal bleeding, record both events separately.

. If a mild non-serious infection develops along with neutropenia, record only neutropenia.

. If severe or serious infection occurs with neutropenia, record both events separately.

**11.5.3. Persistent or recurrent adverse events**

Persistent adverse events are adverse events that are observed continuously without recovery across multiple evaluation time points. These events should be recorded only once, and the severity of the event should be recorded as of the onset of the event. If the event worsens, the severity should always be updated to reflect the highest severity. If the event worsens, update the record to reflect this observation.

Recurrent adverse events are adverse events that disappear between evaluation time points and then recur. If recurrent, each of the events should be documented.

**11.5.4. Abnormal laboratory values**

Not all laboratory abnormalities are considered adverse events, and laboratory results that meet one of the following criteria should be recorded as adverse events.

- Accompanied by clinical symptoms
- Requires changes to be made to investigational drug administration (e.g., discontinuation of administration)
- Requires medical intervention (e.g., potassium supplementation for hypokalemia)
- Findings judged to be clinically significant by the investigator or sub-investigator

The investigator or sub-investigator is responsible for reviewing all laboratory findings. The determination of whether an isolated laboratory abnormality should be classified as an adverse event should be based on medical and scientific rationale.

If the clinically significant laboratory abnormality is an indication of a disease or syndrome (e.g., ALP and bilirubin levels exceeding 5 times the upper limit of normal with cholecystitis), only the diagnosis (e.g., cholecystitis) should be recorded. If a clinically significant laboratory abnormality is not a sign of a disease or syndrome, the abnormality itself should be documented. When entering the value, clearly indicate whether the laboratory result is above or below the normal value (e.g., enter "increased ALP" rather than "abnormal ALP"). If the laboratory abnormality can be described by a precise clinical term that conforms to a common definition, the clinical term must be recorded as an adverse event (e.g., enter "hyperkalemia" for an elevated serum potassium level [7.0 mEq/L]).

The same clinically significant laboratory abnormality over multiple visit dates need not be recorded repeatedly unless the etiology of the event has changed. The initial severity of the event should be entered, updating the severity when the event worsens.

**11.5.5. Abnormal vital signs**

Not all vital sign abnormalities are considered adverse events, but vital sign measurements that meet one of the following criteria should be recorded as an adverse event.

- Accompanied by clinical symptoms
- Requires changes to be made to investigational drug administration (e.g., discontinuation of administration)
- Requires medical intervention (e.g., potassium supplementation for hypokalemia)
- Findings judged to be clinically significant by the investigator or sub-investigator

The investigator or sub-investigator is responsible for reviewing all vital sign findings. The determination of whether a single vital sign abnormality should be classified as an adverse event should be based on medical and scientific rationale.

If a clinically significant vital sign abnormality is a sign of a disease or syndrome (e.g., elevated blood pressure), only the name of the diagnosis (e.g., hypertension) should be documented.

If the same clinically significant vital sign abnormality is observed at the previous visit, do not record it repeatedly unless the underlying cause is altered. The initial severity of the event should be entered, updating the severity when the event worsens.

## 11. 6. Provide new information

When the investigator obtains new information on safety, such as activity and research reports related to the present clinical trial from the investigational coordinator, etc., he/she shall promptly report it in writing to the head of his/her institution and inform all parties involved in the conducting the clinical trial. If the obtained information is a serious matter that could impact the subject's intention to continue clinical trial participation, an additional explanation should be promptly provided to the subject, and re-consent should be obtained after revising the explanatory and consent documents as necessary (refer to the procedure described in "17.6.2. Obtaining re-consent").

In addition, the investigator shall endeavor to collect information on risks directly related to the safety of the life and health of the subject considering treatment with the investigational drug and shall report to the Minister of Health, Labour and Welfare according to the contents of such information when he/she is made aware of matters stipulated in Article 275-3 of the Enforcement Regulations of the Act on Quality, Effectiveness and Safety of Pharmaceuticals, Medical Devices and Other Products.

# 12. Efficacy and Safety Evaluation Committee

An Efficacy and Safety Evaluation Committee comprising experts will be organized for this clinical trial. The Efficacy and Safety Evaluation Committee members will not include persons involved in the present clinical trial. The evaluation procedures of the Efficacy and Safety Evaluation Committee will be specified in a procedure document to be prepared separately.

# 13. Completion, suspension, or discontinuation of the clinical trial

## 13.1. Completion of the clinical trial

The clinical trial will be completed when the investigator has enrolled the target number of subjects, completed the evaluation of all subjects, and verified the contents of all records. The investigator also confirms the contents of all case records at the investigational site and submits a clinical trial completion report to the head of the site upon signing.

## 13.2. Discontinuation or suspension of the entire clinical trial

The coordinating investigator will consider whether or not to continue the clinical trial if any of the following apply. If he/she decides to discontinue the present clinical trial, he/she will promptly inform the investigator and the regulatory authority of the discontinuation in writing, together with the reasons for discontinuation. If the investigator decides to discontinue the present clinical trial, he/she will inform the registration center regarding the discontinuation. The investigator shall promptly notify the head of his/her institution in writing regarding the discontinuation and the reasons for the decision.

1) When substantial information regarding the quality of the clinical trial is obtained

2) If, as a result of evaluating relevant information obtained from sources other than this clinical trial, such as papers and conference presentations, it is determined that there is a problem with the safety of this clinical trial or that the continuation of the clinical trial is no longer meaningful

3) When it is judged difficult to incorporate the target number of cases owing to difficulties in recruiting subjects

4) If the incidence or severity of adverse events in this clinical trial indicates that there is a problem with subject safety

5) When the coordinating investigator decides to discontinue the study

6) If, as a result of evaluating this clinical trial by the Efficacy and Safety Evaluation Committee, it is determined that there is a problem with the safety of this clinical trial

7) When it is recognized that the site has hindered proper clinical trials by violating the drug GCP ordinance or the clinical trial protocol

8) When changes to the clinical trial protocol are necessary, and the site is unable to accommodate such changes

9) When there is an instruction from the head of the site to modify the protocol based on the opinion of the Clinical Trial Review Committee, and the coordinating physician is unable to agree to such modification

10) When the head of the investigational institution recommends discontinuation of the clinical trial based on the decision of the Clinical Trial Review Committee

# 14. Target number of patients and trial duration

## 14.1. Target number of cases

Six cases per group, two groups (12 cases in total)

[Rationale]

Given that the main objective of the present clinical trial is to confirm the safety and PK of meclizine hydrochloride, a statistically based case-count design will not be employed.

The target number of patients is six per group, given that this number has been used in similar clinical trials.

## 14.2. Duration of the clinical trial

Overall duration of the clinical trial: March 2021–March 2022

Case enrollment period: April 2021–January 2022

study period: about 7 weeks (about 4 weeks screening period + 2 weeks dosing period + 1 week follow-up period)

[Rationale]

The t_1/2_ of meclizine in pediatric patients with ACH was 8.5 h, and a 7-day follow-up was used as a guide to confirm safety.

# 15. Statistical analyses

## 15.1. Population to be analyzed

Among the primary endpoints, the safety analysis will be performed on the safety analysis population, and the PK analysis will be performed on the largest analysis population (full analysis set: FAS) as the primary analysis population. In addition, the analysis population that conforms to the protocol (per protocol set: PPS) will also be analyzed to confirm the robustness of the analytical results.

1) Population for safety analysis: Subjects who were enrolled in the study and received at least one dose of the study drug.

2) Maximum analysis population (FAS): Subjects with serious violations of the study protocol (failure to obtain consent, serious violations of study procedures) and subjects with no data after drug administration were excluded from the safety analysis population.

3) Subject population compliant with the study protocol (PPS): Subjects in the FAS, excluding subjects who presented the following violations against the provisions of the study protocol.

(i) Violation regarding inclusion and exclusion criteria (see section "5. Eligible patients and eligibility Criteria")

(ii) Violation of concomitant use of prohibited drugs/adjunctive therapy (see section 7.4.1. "Concurrent use of prohibited drugs/adjunctive therapy")

## 15.2 Analysis items and methods

Details regarding the primary endpoint analysis will be specified in the statistical analysis plan.

**15.2.1. Primary endpoints**

1) Adverse events during the initial 21 days after administering the first dose

Adverse events will be classified and tabulated according to CTCAE v5.0/ MedDRA/J v23.1.

The incidence and 95% confidence interval of all adverse events observed from the first dose to the end of the evaluation will be calculated. The 95% confidence intervals will be calculated using the Clopper-Pearson method. The incidence of adverse events will be calculated by organ category, basic term, severity, and relationship to the study drug. Serious adverse events are calculated in the same way. Serious adverse events are listed by subject.

2) PK

Meclizine plasma drug concentration parameters (Plasma drug concentrations before and after administration of meclizine hydrochloride will be measured for all patients)

(1) Cmax (after the first and 14th doses)

(2) Tmax (after the first and 14th doses)

(3) t_1/2_ (after 14 doses)

(4) AUC (after the first and 14th doses)

## 15.3. Modification of statistical analysis plan

When the statistical analysis plan is changed before data fixation, the reason for the change should be described in the statistical analysis plan and summary report. When the analysis method is changed or additional analysis is performed after data fixation, the reason for the change or additional analysis should be described in the analysis report, as well as in the summary report, and the results of the planned analysis should be distinguished.

## 15.4. Final analysis

After the observation period, all endpoints are analyzed according to the statistical analysis plan after the finalization of data. The results of the final analysis will be summarized in a "Final Analysis Report", and the study coordinating physician and the study coordinating secretariat will summarize the contents of the Final Analysis Report and prepare a "Summary Report" summarizing the conclusions, challenges, interpretation, and discussion of the results of the entire study.

# 16. Quality control and quality assurance

The coordinating investigator must conduct "quality control of clinical trials" and "quality assurance of clinical trials" based on a procedure to be separately prepared to maintain the quality and reliability of the present clinical trial. In addition, the site must cooperate with the investigator's quality control and quality assurance of the clinical trial.

Considering the quality control of clinical trials, the person in charge of monitoring shall confirm that this clinical trial is being conducted in accordance with the "Procedures for Conducting Monitoring" and in compliance with the procedures for operations related to the clinical trial at the site, the latest protocol, and GCP.

In addition, it shall be verified that the contents of the CRF, as reported by the investigator or sub-investigator, are accurate and complete considering the source documents and other clinical trial-related records.

To ensure that the clinical trial is conducted in compliance with the protocol and GCP, the person in charge of auditing shall conduct audits in accordance with the procedure manual to confirm that quality control is properly implemented.

# 17. Ethical matters

## 17.1. Rules and regulations to be observed

This clinical trial will be conducted in compliance with the Declaration of Helsinki, the Pharmaceuticals and Medical Devices Law, its enforcement regulations, GCP, and the clinical trial protocol.

## 17.2. Compliance with the study protocol

The investigators and sub-investigators participating in this clinical trial shall comply with this protocol, provided that it does not compromise the safety and human rights of the subjects.

## 17.3. Changes to clinical trial protocols, etc.

Regardless of the seriousness of revisions, the investigator reports all revisions and the reasons for them to the Institutional Review Board (IRB), and the revisions and reasons for changes are re-reviewed for approval. In addition, the IRB will revise the explanatory and consent documents according to the revised contents.

## 17.4. Deviations from the clinical trial protocol

The investigator or sub-investigator shall not deviate from the contents of the protocol or alter the trial plan without obtaining written approval from the head of the affiliated institution based on prior review by the IRB. However, this excludes cases where the change is unavoidable from a medical perspective, such as to avoid an immediate risk to the subject, or where the change is related only to administrative matters of the clinical trial (e.g., correction of typographical errors, changes in the organizational structure for conducting the clinical trial, change of affiliation, or job title of a person involved in conducting the clinical trial).

When medically unavoidable, for example, to avoid immediate danger to subjects, the investigator or sub-investigator must promptly submit, in writing to the head of the institution, the details and reasons for the deviation or changes and the proposed revision to the protocol, if necessary, and obtain approval from the IRB and the head of the institution.

The investigator or sub-investigator shall record all deviations from the study protocol and the reasons for the deviations. In addition, the investigator must retain these records.

## 17.5. Preparation and revision of the explanation and consent documents

The explanatory document, assent document, and consent document (hereinafter referred to as "Consent Explanation Document") used to obtain consent for participation in the clinical trial from the subject shall be prepared by the investigator and approved by the IRB and the head of the institution to which the subject belongs. If the investigator determines that the consent documents need to be revised, such as when new information is obtained that may affect the subject's consent, the investigator shall promptly revise the consent documents and obtain approval from the IRB and the head of the institution to which the subject belongs.

When information regarding efficacy or safety that may affect the subject's consent is obtained, or when changes are made to the protocol that may affect the subject's consent, the subject will be informed promptly, the subject's consent to participate in the research will be confirmed in advance, and approval from the IRB and the head of the institution to which the subject belongs will be obtained in advance for revising the consent document and obtaining the subject's re-consent. The consent document will be revised with the prior approval of the IRB and the head of the institution to which the subject belongs, and the subject's re-consent will be obtained.

In addition, the explanatory document shall include the following information.

1. The clinical trial must be to test an investigational drug
2. Purpose of the clinical trial
3. Name, title, and contact information of the principal investigator
4. Methods of the study (trial aspects of the study, subject selection criteria, and expected number of participants)
5. Anticipated benefits of the investigational drug on the subject's physical and mental health (if no such benefits are anticipated, a statement to that effect) and anticipated disadvantages to the subject
6. Matters related to other treatment modalities (existence of other treatment modalities and the expected significant benefits and risks associated with such treatment modalities)
7. Duration of clinical trial participation
8. The ability to withdraw from a clinical trial at any time
9. Subjects shall not be treated unfavorably by not participating in or withdrawing from the clinical trial
10. Matters related to the handling of the investigational drug in case of withdrawal from clinical trial participation
11. Access to source documents by monitors, auditors, IRBs, and regulatory authorities, provided that the confidentiality of the subject is maintained
12. Subjects' confidentiality shall be maintained (even when the trial results are made public)
13. Contact information for the implementing medical institution in the event of a health hazard
14. Necessary treatment is provided in the event of health problems
15. Matters related to compensation for health damage
16. Type of IRB that conducts investigation and deliberation on the appropriateness, etc., of the clinical trial, matters to be examined by the IRB, and other matters related to the IRB for the respective clinical trial
17. The subject is immediately informed regarding any information obtained that is deemed to influence the subject's decision to continue clinical trial participation
18. Conditions or reasons for discontinuation of clinical trial participation
19. If the subject is required to bear the cost, a description of the cost
20. If money or other payments are to be made to the subject, the details of such payments (e.g., arrangements for calculating the amount to be paid)
21. Matters related to the handling of the investigational drug in case of clinical trial withdrawal after participation in the clinical trial

## 17.6. Informed consent

**17.6.1. Explanation and obtaining consent**

1. Prior to conducting the clinical trial, the investigator or sub-investigator should hand the latest consent document approved by the Clinical Trial Review Committee to the subject and his/her surrogate and afford a comprehensive explanation. The investigator's collaborator may also provide supplementary explanations. The explanations should be provided using uncomplicated language such that the subject and his/her surrogate can understand the information provided, and any questions of the subject and his/her surrogate should be adequately answered based on the explanatory document for this clinical trial. After confirming that the subject and his/her surrogate have fully understood the information, the subject's free and voluntary consent to participate in this clinical trial will be obtained in writing from the surrogate. If the subject is ≥7 years of age, written consent will also be obtained from the subject.
2. The consent document should be signed by the investigator or sub-investigator who provided the explanation and the subject's surrogate, and the date and time should be indicated for each. If the subject is ≥7 years of age, the assent document should be signed by the investigator or sub-investigator who provided the explanation and the subject, and the date and time should be noted for each. In either case, if a collaborator provides supplementary explanations, the collaborator should also sign and state the date and time of the explanation.
3. If the subject's surrogate cannot read the explanatory document, the investigator or sub-investigator should explain it thoroughly in the presence of an impartial witness and obtain the subject's free and voluntary consent. The witness should also sign the consent document and document the date and time.
4. The investigator or sub-investigator shall provide a copy of the consent document with signature, date, and time, along with the explanatory document, to the subject's surrogate before the subject participates in the clinical trial. In addition, if the subject is ≥7 years of age, a copy of the assent document with the signature, date, and time of the assent document, along with the explanatory document, shall be provided to the subject's surrogate. The original consent document should be stored appropriately in accordance with the regulations of the relevant medical institution.

**17.6.2. Re-obtaining consent**

When information that may influence the subject's intention to continue clinical trial participation is obtained, the investigator or sub-investigator shall promptly convey such information, confirm the subject's intention to continue the trial, and record the results in the medical record. If the explanatory and consent documents are revised, the subject's consent shall be re-obtained again in writing after obtaining IRB approval.

**17.6.3. Notification if the subject is under treatment by another physician**

The investigator or sub-investigator will determine whether the subject for whom consent was obtained is under treatment by another physician (another department or other medical institution at the investigational site). If the subject is receiving treatment from another physician, the investigator or sub-investigator will obtain the subject's consent and notify the respective physician of the subject's intention to participate in this clinical trial until the time of trial discontinuation or the last observation. The fact that this notification has been made shall be recorded in the medical record.

## 17.7. Protection of personal information

Subject registration and subject identification in the CRF will be performed using subject identification codes, etc., to protect the subject's personal information. In the publication of clinical trial results, sufficient consideration will be given to protecting the privacy of subjects' names, diseases, etc. Parties involved in this clinical trial will take the utmost measures to protect personal information, considering the risk of exposing personal information.

The results of this clinical trial may be used in the future to obtain approval as a new treatment or reported in medical journals. In such cases, the results will not contain information that could identify the names or identities of the subjects.

1) Anonymization

In this clinical trial, subject identification codes will be used to ensure the privacy and confidentiality of subjects. The subjects will be anonymized in a linkable manner, and their personal information will be managed appropriately in accordance with the regulations of investigational sites.

2) Disclosure

Although the results of this clinical trial and those of the safety evaluation may be published by the collaborating institutions, various academic societies, academic journals, and databases, appropriate care will be taken to ensure that the data to be disclosed is not personally identifiable. The consent document will explain that there is no concern that the privacy of the subject or his/her family will be leaked to outside parties, although monitoring personnel, audit personnel, IRB, or personnel from the MHLW or the PMDA may request disclosure of the subject's medical record as a result of clinical trial participation. In addition, although mass media (e.g., newspapers and television) may be interested in the subject's case, investigators will ensure that the subject's privacy and confidentiality will be preserved unless the subject gives his/her permission.

# 18. Cost sharing for clinical trials

## 18.1. Costs, sources of funding, and financial relationships related to the clinical trial

The coordinating physician does not receive any funding or labor from the manufacturer of the investigational drug or the pharmacokinetic measurements organization for conducting this clinical trial. In principle, costs associated with each test item in this clinical trial and those associated with hospitalization (excluding basic hospitalization charges) will be borne by the subjects (insurance reimbursement).

The research fund will cover all other costs related to basic inpatient charges and the operation of this clinical trial.

Possible conflicts of interest in this clinical trial shall be handled in accordance with the regulations of the investigational site. The conductance of this clinical trial will not impair the rights and interests of the subjects.

## 18.2. Measures against health hazards

The following items and other necessary measures shall be taken to compensate for any damage to the health of the subject in relation to the clinical trial. The compensation shall provide medical treatment for health damage attributed to the clinical trial despite the proper use of the investigational drug, and payment of compensation for disability and compensation for surviving family members through the clinical trial insurance. Appropriate measures shall be taken in accordance with the "Procedures for Compensation for Injuries to Subjects' Health," which will be separately stipulated.

1) Provision of medical care

Provide the best possible treatment for such health hazards.

2) Payment for disability and survivor's compensation under clinical trial insurance

If an event eligible for disability or survivor's compensation payment occurs, it will be paid by the clinical trial insurance. In this case, the insurance company should be promptly contacted, and necessary measures will be taken. The policy also covers medical expenses and benefits, but the rider should be thoroughly assessed for details. If a subject suffers a health hazard due to negligence in the manufacture of the investigational new drug, this will be paid by the product liability insurance for the purchased investigational new drug.

## 18.3. Payment of money to test subjects

The amount of the burden reduction payment to be paid to subjects in this clinical trial will be determined with reference to the burden reduction payment amount stipulated by each investigational site, and the IRB will approve the date of payment. The expected cost-sharing reduction payment will be provided by the Nagoya University research fund.

# 19. Method of preservation and use of samples, etc. and period of preservation

## 19.1. Preservation of samples, etc.

Typically, blood and urine test specimens are properly discarded after completing the intended test and administration.

The remainder samples for PK measurements will be used for clinical research for biomarker discovery. Before undertaking any research, the relevant clinical research will be approved by the Ethical Review Committee.

## 19.2. Record keeping

All data (e.g., clinical laboratory values, and images) obtained from this clinical trial will be anonymized, and documents and records related to the implementation of the clinical trial (e.g., copies of applications, notification documents from the head of the institution, copies of various applications and reports, correspondence sheets, and other documents or records necessary to ensure the reliability of the data) will be stored in a storage locker in accordance with the regulations of the investigational site. The consent documents should be stored in a storage locker, in accordance with the rules of the investigational site, and disposed of subsequently, taking care to protect personal information. The consent document should be stored in accordance with the regulations of the investigational site and subsequently disposed of, taking care to protect personal information.

1) Clinical trial site

The person in charge of record keeping, as designated by the head of the implementing medical institution, shall preserve documents or records related to clinical trials that need to be preserved at the implementing medical institution until the following 1) or 2), which occurs later. However, if the investigator requires a longer retention period, the site shall discuss the retention period and method with the investigator.

1. The date of approval for the manufacture and sale of the drug under investigation (the date on which three years have elapsed since the date on which the company was made aware that materials of clinical trial results collected through the clinical trial were not to be attached to the application for approval)
2. The date at which 3 years have elapsed since the discontinuation or termination of the clinical trial

2) Principal investigator

The investigator shall retain documents or records pertaining to the clinical trial that must be preserved until the following 1) or 2), which occurs later.

1. The date of approval for the manufacture and sale of the drug under investigation (the date on which three years have elapsed since the date on which the company was made aware that materials of clinical trial results collected through the clinical trial were not to be attached to the application for approval)
2. The date at which 3 years have elapsed since the discontinuation or termination of the clinical trial

3) Clinical Trial Coordinating Physician

The coordinating physician shall retain the documents or records pertaining to the clinical trial until the following 1) or 2), which occurs later.

1. The date of approval for the manufacture and sale of the drug under investigation (the date on which three years have elapsed since the date on which the company was made aware that materials of clinical trial results collected through the clinical trial were not to be attached to the application for approval)
2. The date at which 3 years have elapsed since the discontinuation or termination of the clinical trial

If the study coordinator, investigator, or investigational site permits a company that desires to obtain manufacturing and marketing approval for a meclizine hydrochloride product and attach the results of the present study to its application for manufacturing and marketing approval, the storage of the results after the above storage period has expired will be discussed with the respective company. In addition, if the concerned company obtains manufacturing and marketing approval using the results of the present clinical trial or decides to discontinue development without obtaining approval, the concerned company shall report this to the coordinating investigator in writing.

# 20. Enrollment and progress reporting of clinical trials

Before implementing this clinical trial, the main design of this clinical trial plan will be registered on jRCT (Japanese Research Reporting System for Clinical Trials) and made publicly available.

. jRCT clinical research protocol number: jRCT2041200114

The investigator shall report the progress of the clinical trial to the IRB annually. If the clinical trial is terminated, the investigator shall report to that effect, along with a summary of the results, in writing to the head of his/her institution.

# 21. Attribution of clinical trial results and publication of results

The results of this clinical trial shall belong to Nagoya University. Data obtained from this clinical trial must be submitted to Nagoya University for confirmation prior to publication.

When publishing a paper involving the results of the clinical trial, the paper shall be submitted to an English-language journal after the completion of the final analysis (or, in some cases, after the completion of the interim analysis), in principle. In addition, considering presentations at academic conferences and publication of papers, in principle, the authors of the main publication of the clinical trial results will be determined after consultation with the coordinating physician for the clinical trial and the Department of Advanced Medical Development, Nagoya University Hospital, considering their contribution to this clinical trial. Confidentiality of the subjects will be maintained during publication.

# 22. Organization and structure for conducting clinical trials

The organizations involved in implementing this clinical trial and the affiliation, name, and contact information of the person in charge of each organization shall be indicated on a separate sheet. Any changes shall not be considered changes to the clinical trial protocol, but a revised sheet shall be reported to the clinical trial site.

# 23. References

1. Krejci P, Murakami S, Prochazkova J, et al. NF449 is a novel inhibitor of fibroblast growth factor receptor 3 (FGFR3) signaling active in chondrocytes and J Biol Chem. 2010;285:20644-20653.
2. Jonquoy A, Mugniery E, Benoist-Lasselin C, et al. A novel tyrosine kinase inhibitor restores chondrocyte differentiation and promotes bone growth in a gain-of-function Fgfr3 mouse model. Hum Mol Genet. 2012;21:841-851.
3. Yasoda A, Komatsu Y, Chusho H, et al. Overexpression of CNP in chondrocytes rescues achondroplasia through a MAPK-dependent pathway. Nat Med. 2004;10:80-86.
4. Lorget F, Kaci N, Peng J, et al. Evaluation of the therapeutic potential of a CNP analog in a Fgfr3 mouse model recapitulating achondroplasia. Am J Hum Genet. 2012;91:1108-1114.
5. Yamashita A, Morioka M, Kishi H, et al. Statin treatment rescues FGFR3 skeletal dysplasia phenotypes. Nature. 2014;513:507-511.
6. Fagan JE, Kaplan BJ, Raymond JE, Edgington ES. The failure of antimotion sickness medication to improve reading in developmental dyslexia: results of a randomized trial. J Dev Behav Pediatr. 1988;9(6):359-366.
7. Kitoh H, Matsushita M et al. Pharmacokinetics and safety after once and twice a day doses of meclizine hydrochloride administered to children with achondroplasia. PLoS One. 2020;15(4):e0229639.
