# Supplementary material for: Phase 1b study on the repurposing of meclizine hydrochloride for children with achondroplasia
Source: PLoS One. 2023 Jul 10;18(7):e0283425. doi: 10.1371/journal.pone.0283425 (PMC10332602; doi:10.1371/journal.pone.0283425)
Supplement: S2 File — (DOCX) [file pone.0283425.s003.docx]

**治験実施計画書**

**軟骨無形成症患者（小児）に対する塩酸メクリジン製剤の**

**2週間連続投与後の安全性及び薬物動態の検討**

**＜治験調整医師＞**

名古屋大学医学部附属病院・整形外科

病院講師　松下　雅樹

**＜治験調整事務局＞**

名古屋大学医学部附属病院 先端医療開発部

中井康博

〒466-8560 名古屋市昭和区鶴舞町 65

TEL：052-744-2942　 FAX：052-744-1303

実施計画書番号 ：　CAMCR-015

版数・作成日 ：　第 4.0 版　2021年 11月 2 日

# Summary of the protocol

| **Principal investigator：**  Masaki Matsushita |
| --- |
| **Title：**  Pharmacokinetics and safety of meclizine hydrochloride administered to achondroplasia children for 14 days |
| **Investigational drugs：**  1. Non-proprietary name： Meclizine hydrochloride  2. Name of investigational drug：MECLIZIN  3. Component and dose： Meclizine 12.5 mg was contained in one tablet. The tablets contained D-mannitol, cellulose, carmellose sodium, hardened oil, magnesium stearate, and l-menthol as excipients  4.Dosage form：Tablet |
| **Trial number：**  CAMCR-015 |
| **Number of conduct medical institution：**  2 institutions (Nagoya University Hospital and Aichi Children’s Health and Medical Center) |
| **Trial design：**  Multicenter unblinded and uncontrolled trial |
| **Purpose of the study：**  We administer meclizine hydrochloride to achondroplasia children for 14 days to examine pharmacokinetics at the first and last days of administration and safety evaluation. |
| **Subjects：**  achondroplasia：ACH |
| **Target number：**  A total of 12 cases. 6 cases per one group |
| **Reason of target sample size：**  Target sample size is not statistically determined because purpose of the study is to evaluate safety and pharmacokinetics, but determined based on the sample size of similar trials. |
| **Clinical trial period：**  Total trail period: From Mar., 2021 to Mar., 2022  Patient registration period: From Apr., 2021 to Jan., 2022  Trial period for patients: About 49 days (Obtaining consent and screening period 28 days + administration period 14 days + follow-up period 7 days) |
| **Inclusion criteria：**  Patients who meet all the following conditions  1) Patients who were diagnosed as achondroplasia one year or more before consent was obtained based on the diagnostic criteria established by the study group of Ministry of Health, Labour, and Welfare  2) Patients aged from 5 to younger than 11 years at the time of obtaining consent  3) Patients who can be hospitalized at the last day of the drug administration and are expected to complete this trial during the evaluation period  4) Patients whose consent was obtained in writing by a representative  5) Patients who obtained an ascent in writing when the subject is 7 years of age or older  6) Patients who can take tablets |
| **Exclusion criteria：**  Exclude patients who comply with any of the followings  1) Patients who took a drug containing meclizine hydrochloride within 28 days before administration of meclizine  2) Patients who underwent limb lengthening within 28 days prior to administration of meclizine or who are scheduled to undergo limb lengthening during the study period  3) Patients with serious complications  4) Patients whose weight of less than 11 kg  5) Patients with symptoms of dysuria  6) Patients who have been diagnosed as glaucoma  7) Patients who are allergic to meclizine hydrochloride  8) Patients who regularly took a drug containing common cold medicine, antipyretic analgesic, sedative, antitussive expectorant, antihistamine  9) In addition, patients who were decided inappropriate by the investigator or subinvestigator in conducting the trial |
| **Medication methods：**  Oral administration of one or two tablets a day for 14 days. One tablet contains meclizine 12.5 mg. |
| **Evaluation items：**  [Primary endpoints]  Safety: Name, extent, and rate of adverse events or side effects  Pharmacokinetics: Plasma concentration of meclizine  1) Cmax (during 24 hours)  2) Tmax (during 24 hours)  3) t 1/2 (during 24 hours)  4) AUC (during 24 hours) |
| **Reason of primary endpoint：**  There has been no data of evaluation for safety and pharmacokinetics after 14-day-repeated administration of meclizine for children. Since the purpose of the study is to evaluate the safety and pharmacokinetics, these items are included as primary endpoints. |
| **Statistics methods：**  [Primary endpoints]  Safety: Adverse events (AEs) for 21 days after the first administration  AEs were classified based on the Common Terminology Criteria for Adverse Events (CTCAE v5.0/MedDRA/J v23.1). Incidence and 95% confidence interval of each AE were calculated according to the Clopper-Pearson exact method.  Pharmacokinetics: 1) C_max_ and 2) Tmax are calculated based on the meclizine concentration for 6 and 24 hours after the initial and last doses, respectively. 3) t1/2 is calculated based on the meclizine concentration for 24 hours after the last dose. 4) AUC_0-6_ after the initial and last doses and AUC_0-24_ after the last dose are calculated. |
| **Prohibited drugs and therapies：**   1. Concomitant drugs   Following drugs is prohibited from 28 days before administration of meclizine until the end of the evaluation period.  1) Drugs that have been reported to have the ability to suppress FGFR3 signaling (CNP analogues not approved in Japan, statins etc.)  2) Drugs such as anti-motion sickness drugs containing meclizine hydrochiloride  3) Common cold medicine, antipyretic analgesic, sedative, antitussive expectorant, internal medicine containing antihistamine (internal medicine for rhinitis, medicine for allergy etc.) are prohibited within 24 hours before administration of meclizine and within 24 hours after completion of administration.   1. Concomitant treatments   Following treatments are prohibited during the period from obtaining consent to the end of the evaluation period.  1) Patients who were treated with growth hormone at the time of obtaining consent do not discontinue or change treatment until the end of the evaluation period.  2) Limb lengthening surgery |

| **秘密保全に関する記述**  本文書中の情報は、本治験の直接関係者等に限定して提供しています。したがって、治験に参加する被験者から同意を取得する場合を除き、治験責任医師の事前の許可を得ることなく、本治験実施計画書に含まれる情報の使用、漏洩、公表、本治験と関係のない第三者への開示を禁じます。 |
| --- |

# 略語及び用語の定義の一覧

略語一覧

| 略語 | 省略していない表現又は定義 |
| --- | --- |
| ACH | Achondroplasia（軟骨無形成症） |
| AUC | Area under the curve (血中濃度曲線下面積) |
| Cmax | maximum concentration (最高血中濃度) |
| CNP | C-type Natriuretic Peptide (C型ナトリウム利尿ペプチド） |
| CRF | Case Report Form （症例報告書） |
| FAS | full analysis set (最大の解析対象集団) |
| FGFR3 | Fibroblast growth factor receptor 3（線維芽細胞増殖因子受容体3） |
| GCP | Good Clinical Practice（医薬品・医療機器等の臨床試験の実施に関する基準） |
| PPS | per protocol set (試験計画書遵守集団) |
| Tmax | Time to reach Cmax (最高血中濃度到達時間) |
| t 1/2 | Elimination half-life (血中濃度半減期) |
|  |  |

臨床検査項目の略号一覧

| 略号 | 省略していない表現用語 |
| --- | --- |
| TP | Total protein（総蛋白） |
| Alb | Albumin（アルブミン） |
| ALT | Alanine Aminotransferase(アラニンアミノ基転移酵素) |
| AST | Aspartate Aminotransferase（アスパラギン酸アミノ基転移酵素） |
| BUN | Blood urea nitrogen（尿素窒素） |
| Cl | Chlorine（クロール） |
| Cre | Creatinine（クレアチニン） |
| CRP | C-Reactive Protein（C反応性タンパク） |
| K | Potassium（カリウム） |
| Na | Sodium（ナトリウム） |
| TG | Triglyceride（トリグリセライド）中性脂肪 |

用語一覧

| 用語 | 定義 |
| --- | --- |
| 医薬品GCP省令 | 医薬品の臨床試験の実施の基準に関する省令（平成9年3月27日厚生労働省令第28号） |
| ヘルシンキ宣言 | 世界医師会ヘルシンキ宣言（2013年10月改訂版） |
|  |  |

[略語及び用語の定義の一覧 1](#_Toc62648634)

[概要 4](#_Toc62648635)

[1. 治験の目的 9](#_Toc62648636)

[1.1. 試験デザイン 9](#_Toc62648637)

[1.2. 主要評価項目 9](#_Toc62648638)

[2. 治験の背景と根拠 9](#_Toc62648639)

[2.1. 軟骨無形成症について 9](#_Toc62648640)

[2.3. 治験薬について 10](#_Toc62648641)

[2.4. 軟骨無形成症に対する本治験薬の有効性について 11](#_Toc62648642)

[2.5. 本治験実施について 11](#_Toc62648643)

[3. 治験薬の概要 11](#_Toc62648644)

[3.1. 治験薬 11](#_Toc62648645)

[3.2. 治験薬の製剤組成 12](#_Toc62648646)

[3.3. 包装及び表示 12](#_Toc62648647)

[3.4. 治験薬の取扱い、保管及び管理 13](#_Toc62648648)

[4. 研究計画・研究デザイン 13](#_Toc62648649)

[4.1. 治験の種類・デザイン 13](#_Toc62648650)

[4.2. 治験のアウトライン 13](#_Toc62648651)

[5. 対象患者及び適格性の基準 13](#_Toc62648652)

[5.1. 選択基準 13](#_Toc62648653)

[5.2. 除外基準 14](#_Toc62648654)

[6 登録 14](#_Toc62648655)

[6.1. 登録の手順 14](#_Toc62648656)

[6.2. 登録に際しての注意事項 14](#_Toc62648657)

[6.3. 本治験の被験者として不適格と判断された場合 15](#_Toc62648658)

[7. 治験の方法 15](#_Toc62648659)

[7.1. 用法・用量 15](#_Toc62648660)

[7.2. 各被験者の治験期間 15](#_Toc62648661)

[7.3. 治験終了後の治験薬の使用 15](#_Toc62648662)

[7.4. 併用薬・併用療法に関する規定 16](#_Toc62648663)

[8. 評価項目 16](#_Toc62648664)

[8.1. 主要評価項目 16](#_Toc62648665)

[9. 観察及び検査項目及び実施時期 18](#_Toc62648666)

[9.1. 観察及び検査項目 20](#_Toc62648667)

[9.2. 観察・検査方法 23](#_Toc62648668)

[10. 中止基準 23](#_Toc62648669)

[11. 有害事象 24](#_Toc62648670)

[11.1. 有害事象発現時の被験者への対応、並びに有害事象の調査と報告 24](#_Toc62648671)

[11.2. 有害事象等の定義 24](#_Toc62648672)

[11.3. 重篤な有害事象が発現した場合の対応 26](#_Toc62648673)

[11.4. 本治験において予測される有害事象等 26](#_Toc62648674)

[11.5. 有害事象記録手順 27](#_Toc62648675)

[11.6. 新たな情報の提供 28](#_Toc62648676)

[12. 効果安全性評価委員会 29](#_Toc62648677)

[13. 治験の終了、中止･中断 29](#_Toc62648678)

[13.1. 治験の終了 29](#_Toc62648679)

[13.2. 治験全体の中止･中断 29](#_Toc62648680)

[14. 目標症例数と治験期間 29](#_Toc62648681)

[14.1. 目標症例数 29](#_Toc62648682)

[14.2. 治験期間 30](#_Toc62648683)

[15. 統計的事項 30](#_Toc62648684)

[15.1. 解析対象集団 30](#_Toc62648685)

[15.2. 解析項目･方法 30](#_Toc62648686)

[15.3. 統計解析計画書の変更 31](#_Toc62648687)

[15.4. 最終解析 31](#_Toc62648688)

[16. 品質管理及び品質保証 31](#_Toc62648689)

[17. 倫理的事項 31](#_Toc62648690)

[17.1. 遵守すべき諸規則 31](#_Toc62648691)

[17.2. 治験実施計画書の遵守 31](#_Toc62648692)

[17.3. 治験実施計画書等の変更 32](#_Toc62648693)

[17.4. 治験実施計画書からの逸脱について 32](#_Toc62648694)

[17.5. 説明文書及び同意文書の作成と改訂 32](#_Toc62648695)

[17.6. インフォームド・コンセント 33](#_Toc62648696)

[17.7. 個人情報の保護 34](#_Toc62648697)

[18. 治験の費用負担 34](#_Toc62648698)

[18.1. 治験に関する費用、資金源及び財政上の関係 34](#_Toc62648699)

[18.2. 健康被害に対する措置 35](#_Toc62648700)

[18.3. 被験者に対する金銭の支払い 35](#_Toc62648701)

[19. 試料等の保存及び使用方法並びに保存期間 35](#_Toc62648702)

[19.1. 試料等の保存等について 35](#_Toc62648703)

[19.2. 記録の保存 35](#_Toc62648704)

[20. 治験の登録と進捗状況の報告 36](#_Toc62648705)

[21. 治験成果の帰属と結果の公表 36](#_Toc62648706)

[22. 治験実施組織・体制 37](#_Toc62648707)

[23. 参考資料･文献リスト 38](#_Toc62648708)

# 概要

| **治験調整医師：**  名古屋大学医学部附属病院・整形外科　病院講師　松下雅樹 |
| --- |
| **課題名：**  軟骨無形成症患者（小児）に対する塩酸メクリジン製剤の２週間連続投与後の安全性及び薬物動態の検討 |
| **治験薬：**  1. 一般名：メクリジン塩酸塩  2. 治験薬の名称：MECLIZIN  3. 成分及び分量：1錠中にメクリジン塩酸塩を12.5mg含有、添加物としてD-マンニトール、セルロース、カルメロースNa、硬化油、ステアリン酸Mg、*l*-メントールを含む  4. 剤形：錠剤 |
| **実施計画書番号：**  CAMCR-015 |
| **医療機関数：**  2施設（名古屋大学医学部附属病院、あいち小児保健医療総合センター） |
| **試験デザイン：**  多施設非盲検非対照試験 |
| **主な目的：**  軟骨無形成症の小児患者を対象に塩酸メクリジン製剤を1日1回14日間投与し、投与初日及び最終投与日の薬物動態の検討を行うとともに、併せて安全性を検討する。 |
| **対象疾患：**  軟骨無形成症（achondroplasia：ACH）患者 |
| **目標症例数：**  1群6例　2群構成　計12 例 |
| **症例数設定根拠：**  本治験の主目的は、塩酸メクリジンの安全性及び薬物動態を確認することであるため、統計学的根拠に基づく症例数設計は行わない。  類似の治験にて多く用いられる症例数として、目標症例数は、1群6例とする。 |
| **治験期間：**  治験全体の実施期間：2021年3月～2022年3月  症例登録期間：2021年4月～2022年1月  被験者の治験期間：49日間程度（同意取得、スクリーニング28日間＋投与期間14日間＋フォローアップ期間7日間） |
| **選択基準：**  以下の条件をすべて満たす患者   1. 同意取得時1年以上前に厚労省難病研究班により作成された軟骨無形成症診断基準のうち、DefiniteあるいはProbableに該当するものと確定診断された患者 2. 同意取得時の年齢が 5 歳以上 11 歳未満の患者 3. 最終投与日に入院が可能で、本治験の完遂が見込める患者 4. 代諾者により、文書にて同意が得られた患者 5. 被験者が7歳以上においては本人より、文書にてアセントが得られた患者 6. 錠剤を服用することができる患者   ［設定根拠］   1. 治療対象となる患者集団として設定した。 2. 被験薬の薬理作用は骨端線が閉鎖した場合、期待できず、薬効を得るには骨端線の閉鎖する思春期以前の投与が必要である。有効性は第2相試験で評価する予定であるが、5歳以上11歳未満の軟骨無形成症患者の成長速度は性別、年齢によらず年間4cm（全国調査に基づいた軟骨無形成症患児の身長の検討）であり、成長曲線との比較により第2相試験で有効性評価が可能な患者集団と同一の集団として設定した。臨床至適用法・用量の推定を行うためには、患児を対象とした薬物濃度推移、動態のデータを取得することが必要である為、対象集団として設定した。 3. 治験計画書に従い治験が実施できる患者として設定した。   ④,⑤被験者からの同意取得を原則とするが、年齢を勘案し、代諾者のみからの取得でも治験参加を可とするため、設定した。  ⑥治験薬が内服用錠剤であるため設定した。 |
| **除外基準：**  下記のいずれかに該当する患者は除外する。   1. 投与開始前 28日以内に、塩酸メクリジンを含有する薬剤を服用した患者 2. 投与開始前 28日以内に、骨延長術を実施した患者または治験期間中に骨延長術の実施を予定している患者 3. 重篤な合併症を有する患者 4. 体重11kg未満の患者 5. 排尿困難の症状がある患者 6. 緑内障の診断を受けた患者 7. 塩酸メクリジンに対するアレルギーを有する患者 8. かぜ薬, 解熱鎮痛薬, 鎮静薬, 鎮咳去痰薬, 抗ヒスタミン剤を含有する内服薬を常用している患者 9. その他、治験責任医師又は治験分担医師が本治験を実施するにあたり、不適当と判断した患者   ［設定根拠］   1. 薬剤の安全性、薬物動態を適切に評価するため、設定した。   ②~⑨被験者の安全性を確保するために設定した。 |
| **用法・用量：**  1回1錠または2錠を1日1回14日間経口投与する。1錠に塩酸メクリジンとして12.5mgを含む。  ［投与量の設定根拠］  塩酸メクリジン25mgを1日1回または2回投与後の薬物動態を解析した所、至適と考える暴露量より高値であった為、半量の12.5mgを設定した、また体内動態の変動を考慮し25mgを投与する群を設定する、12.5mg投与群のフォローアップ終了後、安全性が確認された後25mg投与群を開始する。 |
| **評価項目：**  【主要評価項目】  安全性 ：すべての有害事象の事象名、程度、発現頻度  薬物動態：メクリジンの血漿中薬物濃度パラメータ  ① Cmax (投与24時間まで)  ② Tmax (投与24時間まで)  ③ t 1/2 (投与24時間まで)  ④ AUC (投与24時間まで) |
| **主要評価項目の設定根拠：**  塩酸メクリジンを現在の科学水準にて小児に連続投与した記録は無く、その安全性及び薬物動態を評価することが主な目的であるため、安全性、薬物動態を主要評価項目とした。 |
| **統計解析方法：**  1. 主要評価項目  ・安全性： 初回投与から21日間の有害事象  有害事象は、CTCAE v5.0/ MedDRA/J v23.1で分類、集計する。  初回投与から評価終了までに認められた総ての有害事象の発現率及び95%信頼区間を算出する。95%信頼区間はClopper-Pearson 法を用いて算出する。また、有害事象を器官大分類別、基本語別、重症度別及び試験薬との関連性別に発現率を算出する。重篤な有害事象も同様に算出する。  ・薬物動態：初回投与後6時間及び14回投与後24時間までの血漿中メクリジン濃度を基に  ① Cmax  ② Tmax  を算出する。  また、  14回投与後24時間までの血漿中メクリジン濃度を基に  ③ t 1/2 (投与24時間まで)  初回投与後6時間までおよび14回投与後24時間までの血漿中メクリジン濃度を基に  ④ AUC投与6時間まで(初回及び14回投与後)及び24時間まで（14回投与後）  を算出する。 |
| **臨床試験の併用禁止薬・併用禁止療法：**   1. 併用禁止薬   投与開始28日前から評価期間終了までの期間、次の薬剤の併用を禁止とする。  ① FGFR 3シグナルを抑制する成績が発表されている薬剤 （国内未承認の CNP アナログ製剤、スタチン製剤等）  ② 塩酸メクリジンを含有する酔い止め薬等の薬剤  治験薬投与開始24時間前から投与終了後24時間以内は、次の薬剤の併用を禁止とする。  ③ かぜ薬, 解熱鎮痛薬, 鎮静薬, 鎮咳去痰薬, 抗ヒスタミン剤を含有する内服薬（鼻炎用内服薬, アレルギー用薬等）   1. 併用禁止療法   同意取得後から評価期間終了までの期間、以下の治療を禁止する。  ① 同意取得時に成長ホルモン製剤による治療を受けている患者は、評価期間終了まで治療の中止、変更を禁止する。  ② 骨延長術による治療は禁止する。 |
| **評価スケジュール：**  ****  PK採血表    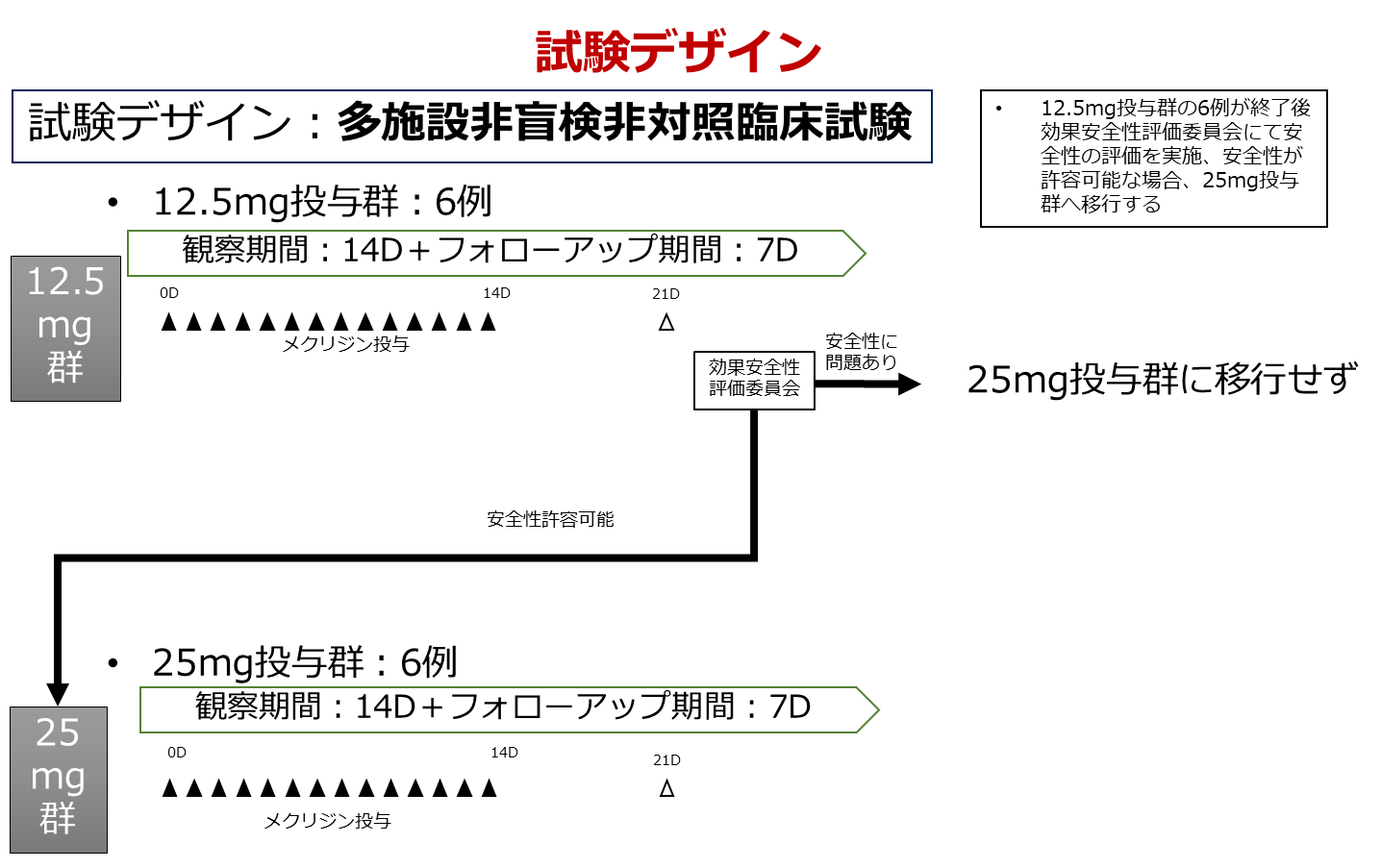   |

# 1. 治験の目的

軟骨無形成症の小児患者を対象に塩酸メクリジン製剤を14日間反復経口投与し、薬物動態、並びに安全性を検討する。

## 1.1. 試験デザイン

多施設非盲検非対照試験

## 1.2. 主要評価項目

安全性 ：初回投与から21日間の有害事象の事象名、程度、発現頻度

薬物動態：メクリジンの血漿中薬物濃度パラメータ

① Cmax (初回及び14回投与後各々)

② Tmax (初回及び14回投与後各々)

③ t 1/2 (14回投与後)

④ AUC (初回及び14回投与後各々)

# 2. 治験の背景と根拠

## 2.1. 軟骨無形成症について

軟骨無形成症（achondroplasia: ACH）は、最も頻度の高い四肢短縮型低身長を呈する骨系統疾患で、骨伸長の抑制因子である線維芽細胞増殖因子受容体 3 (fibroblast growth factor receptor 3：FGFR3)の機能獲得型変異による過剰な FGFR3 活性化が原因であり、低身長に加えて脊柱管狭窄症・大後頭孔狭窄などの重篤な合併症も生じる。特に四肢骨の長軸方向への成長が障害されることにより成人における最終身長は 120〜130cm であり日常生活に多大な支障をきたす。現在、軟骨無形成症における過剰に活性化したFGFR3シグナルを抑制する根本的治療はなく、低身長に対する対症的な治療として内科的には成長ホルモン治療、外科的には骨延長術が行われているが、前者は有効性に乏しく、後者は侵襲性が高いのが難点である。 近年、FGFR3シグナルを抑制する複数の低分子化合物が同定されたが、これら化合物は非臨床試験での安全性が担保されていない1）2）。生理活性物質である C 型ナトリウム利尿ペプチド（C-type natriuretic peptide : CNP）は ACH モデル動物において有効性が示されたが、半減期が数分と著しく短い3）。また、米国で開発された CNP アナログ（BMN111）は臨床試験を実施中であるが、注射剤であり小児への長期投与は負荷が大きい4）。iPS 細胞を使用したスクリーニング系によりスタチンのFGFR3シグナル抑制効果が報告されたが、至適使用量や期間、副作用に関する検討はなされていない5）。 日常生活に多大な支障を及ぼすACH に対する治療は、小児期から治療開始が必要となるため、侵襲性が低い根本的治療薬の開発が必要であると考える。

2020年6月25日にPMDAの対面助言を実施し、次のような助言を受けた。

実施済みの非臨床試験成績等を踏まえ、非臨床試験の充足性の観点からは、相談者の提示する第 I 相（2 週間連投）試験及び第 I 相（継続）試験を開始することは可能と考える。第 I 相（2 週間連投）試験の本剤の用法・用量について、安全性の観点からは、12.5 mg 1 日 1 回投与で本試験を開始し、12.5 mg 1 日 1 回投与 6 例の安全性情報を踏まえて 25 mg 1 日 1 回投与を開始することは可能と考える。

以上、PMDAの助言を踏まえ、小児 ACH 患者を対象とした塩酸メクリジン 12.5 mg錠 の1 日 1 回1錠または2錠を14日間投与時における薬物動態及び安全性を検討する試験を計画した。

また、PMDAに提示した第 I 相（継続）試験は実施しないこととした。

## 2.3. 治験薬について

塩酸メクリジンは米国において1957年2月14日に乗り物酔い症状の緩和の効能で承認された処方箋医薬品である。また、本邦においては一般用医薬品（第二類医薬品）として1981年から発売されており、3才以上が適用対象となっている。このように、塩酸メクリジンは先進国において60年以上の使用実績があり、この間、日米の薬事規制当局から安全面で規制を加える必要があるとの見解が出されたことはない。また、9歳9か月～14歳8か月の学習障害児6名に、50mg/日（25mgを2回）を3カ月間反復投与した報告では副作用発現は報告されていない6）。

塩酸メクリジンは国内外で古くから医療用あるいは一般用医薬品として使用されているが、治験実施に利用可能な非臨床安全性試験データは揃っていない。そこで、PMDA薬事戦略相談の助言を受けて、ラットおよびイヌの単回投与TK試験、1週間および2週間反復投与予備毒性試験を実施した。2 週間反復投与試験では、ラットにおいては 150 mg/kg 群で死亡例が認められ、50 及び 150 mg/kg 群において 肝細胞の空胞（脂肪滴）、肺胞内及び子宮内膜における泡沫細胞浸潤が、150 mg/kg 群において脾臓の空胞化、腎臓の近位尿細管上皮の空胞化、副腎皮質細胞における脂肪滴の増加が認められ、脂質系への影響が示唆された。その他、雄の乳腺の萎縮および雌の乳腺の過形成が150 mg/kg群，眼球水晶体線維の膨化が150 mg/kg群の雄，50 及び150 mg/kg群の雌，精巣の精細管内における異型残渣体の出現及び精子細胞遺残，及び精巣上体管内における細胞残渣の増加が150 mg/kg群の雄で認められ、無毒性量は，雌雄ともに15 mg/kg と結論した。イヌにおいては、肝臓の腫大が雄では300 mg/kg以上の用量群の全例，雌では100 mg/kg以上の用量群の全例で認められ、血液生化学的パラメータの変化より無毒性量は，雄では 300 mg/kg，雌では100 mg/kgであり、種差を認めた。追加実施した蛋白結合率・肝代謝固有クリアランス・代謝物プロファイリング試験の結果から、ラットではヒト、イヌで認められない代謝物（二水酸化体）の存在を認めた。さらに、遺伝毒性試験および安全性薬理試験を実施して安全性を確認し、2017年3月までに「医薬品の臨床試験及び製造販売承認申請のための非臨床安全性試験の実施についてのガイダンスについて」（平成 22 年 2 月 19 日付薬食審査発 0219 第 4 号、ICH M3（R2）ガイダンス）に基づいた新規有効成分の臨床第1相試験の治験届を提出するのに必要な非臨床安全性試験を完了した。

更にビーグル犬を用いた13週間反復経口投与毒性試験では低投与量群（100mg/kg）において肝重量の増加、肝細胞の顆粒状変化、ALPの高値、高投与量群（300mg/kg）において肝重量の増加、肝細胞の空胞化、顆粒状変化及び肝細胞肥大、ALT、ALPの高値、グルコースの低値、体重減少、が認められた。無毒性量（NOAEL）は雌雄ともに100 mg/kg/day未満と推定された。

また、幼若ラットにおける4週間経口投与毒性試験及び7週間回復性試験において高投与量群投与後にラット2週間反復経口投与毒性試験にて観察された、雌雄における肝細胞空胞、眼球水晶体の膨化、肺胞に泡沫細胞浸潤が、雄において精巣に異型残渣体の出現，巨細胞及び精子細胞の変性，精巣上体に細胞残渣が観察され、雌において黄体未観察、発情期の持続が観察されたが、休薬期間終了後には回復傾向が認められた、無毒性量（NOAEL）は雌雄ともに50 mg/kg/dayと判断した。

## 2.4. 軟骨無形成症に対する本治験薬の有効性について

2013年、名古屋大学大学院医学研究科の鬼頭、松下、大野らはACHモデル細胞に1,186種類のFDA既承認薬を添加した網羅的薬効スクリーニング法により、塩酸メクリジンが各種軟骨系細胞において、FGFR3下流のMAPK経路においてErkのリン酸化を抑制することを見出した。また、胎生期マウス脛骨器官培養系において、塩酸メクリジンは骨伸長作用を示した。さらに、成長期のACHモデルマウスに乗り物酔い防止薬で得られる血漿中濃度の範囲内に相当する塩酸メクリジンを混餌経口投与したところ、モデルマウスの骨伸長をレスキューした。単回投与PK試験において、マウスでは2mg/kg投与で乗り物酔い薬としての塩酸メクリジンの臨床使用量(25mg)と同レベルでの血中濃度が得られることを確認した。そこで定量的に薬剤を経口投与したところ、塩酸メクリジン1mg/kg/dayおよび2mg/kg/dayの1日2回経口投与により濃度依存性にモデルマウスの骨長が促進した。組織学的にも成長軟骨帯における肥大軟骨細胞層の幅の拡大を認めた。また、塩酸メクリジン投与により長管骨や脊椎の骨量が増大するとともに、骨幹端部の骨質の改善も認め、in vivoにおけるFGFR3抑制作用を確証した。

## 2.5. 本治験実施について

2018年7月から2018年11月に12例のACH患児に対する単回投与第1相試験を終了した7）。ACH患児に1回あたり25mgの塩酸メクリジンを投与した後に安全性の懸念は認められず、薬物動態の結果からは蓄積性の懸念は認められなかった。患者に対する本治験薬の有効性が期待できる非臨床試験もあり、また作用メカニズムから勘案すると、患者に高い有効性を示す可能性は高いと考えており、患者を対象にした本治験薬の反復投与後の安全性、薬物動態を確認する臨床試験の実施が必要であると考えた。

# 3. 治験薬の概要

## 3.1. 治験薬

1） 治験薬の識別記号：MECLIZIN

2） 成分：［一般名］メクリジン塩酸塩

［和名］塩酸メクリジン　（局外規）

［国際名称］Meclizine (Meclozine) HCl

分子式：C25H27Cl N2,２HCl・H2O

分子量：481.89

構造式：
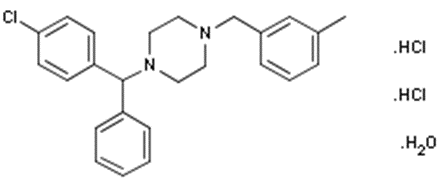


3） 剤型・含量：一般用医薬品（第二類医薬品）として承認されているソラシドンの処方、製造法を参考とし、新たに明治薬品にて製造した12.5mgの塩酸メクリジンを含む製剤を使用する。

4）治験薬提供者：名古屋大学医学部附属病院　愛知県名古屋市昭和区鶴舞町65

## 3.2. 治験薬の製剤組成

ソラシドン臨床試用（明治薬品）：

1錠中に塩酸メクリジン 12.5mg を含有

（添加物）D-マンニトール、セルロース、カルメロースNa、硬化油、ステアリン酸Mg、 *l*-メントール

有効期限：室温で製造後36ヵ月（暫定）

## 3.3. 包装及び表示

治験薬ラベルには治験用であること、識別記号、一般的名称、治験調整医師の氏名、所属、職名及び住所を表示する。

包装形態：錠剤をPTPシートに包装

PTPシートは紙製の箱（被包）に封入されている。

表示：被包に貼付されるラベルには、以下の内容を含むものとする。

① 治験用である旨

② 識別記号

③ 製造番号（Lot.）

④一般的名称

⑤ 保管及び取り扱いの注意

⑥ 有効期間

⑦治験調整医師の所属、氏名及び職名ならびに住所

注：職名は2021年7月1日より病院講師に変更となっているが、読み替えにて対応する。

| **治験用** | **軟骨無形成症患者（小児）に対する塩酸メクリジン製剤の２週間連続投与後の安全性及び薬物動態の検討** |
| --- | --- |
|  | **本治験以外の目的で使用しないこと** |
| 識別記号： | MECLIZIN |
| 製造番号： | *** |
| 一　般　的　名　称　： | メクリジン塩酸塩 |
| 保管及び取扱いの注意： | 直射日光・湿気を避けて保管 |
| 有効期間　　　　　　： | 2023年7月（暫定） |
| 治験調整医師： | 名古屋大学医学部附属病院・整形外科  特任助教　松下雅樹  愛知県名古屋市昭和区鶴舞町65 |

## 3.4. 治験薬の取扱い、保管及び管理

治験調整医師は、臨床試用として製造されたソラシドン臨床試用を購入し、ラベルの表示変更をすることによって治験薬とする。治験薬管理者は、治験調整医師が作成した「治験薬の管理に関する手順書」に従い、直射日光の当たらない湿気の少ない涼しい場所で治験薬を保管、管理する。

　保管温度：室温

# 4. 研究計画・研究デザイン

## 4.1. 治験の種類・デザイン

第1相試験・多施設非盲検非対照試験

## 4.2. 治験のアウトライン

本治験では、安全性の検討を行うとともに、軟骨無形成症の小児患者の薬物動態データを収集する。すなわち、1日1回14日間投与のメクリジンの血漿中薬物濃度から、第2相長期反復投与試験開始のための、根拠となるデータを取得するとともに、安全性データの充足につなげることが目的である。薬物濃度が指標であり盲検化は不要と判断した。

# 5. 対象患者及び適格性の基準

同意取得後、登録時に5.1. 選択基準①～⑥を満たし、かつ5.2. 除外基準①～⑧のいずれにも抵触しない患者を対象とする。

## 5.1. 選択基準

1. 同意取得時1年以上前に厚労省難病研究班により作成された軟骨無形成症診断基準のうち、DefiniteあるいはProbableに該当するものと確定診断された患者
2. 同意取得時の年齢が 5 歳以上 11 歳未満の患者
3. 最終投与日に入院が可能で、本治験の完遂が見込める患者
4. 代諾者により、文書にて同意が得られた患者
5. 被験者が7歳以上においては本人より、文書にてアセントが得られた患者
6. 錠剤を服用することができる患者

［設定根拠］

① 　治療対象となる患者集団として設定した。

②　　 被験薬の薬理作用は骨端線が閉鎖した場合、期待できず、薬効を得るには骨端線の閉鎖する思春期以前の投与が必要である。有効性は第2相試験で評価する予定であるが、5歳以上11歳未満の軟骨無形成症患者の成長速度は性別、年齢によらず年間4cm（全国調査に基づいた軟骨無形成症患児の身長の検討）であり、成長曲線との比較により第2相試験で有効性評価が可能な患者集団と同一の集団として設定した。臨床至適用法・用量の推定を行うためには、患児を対象とした薬物濃度推移、動態のデータを取得することが必要である為、対象集団として設定した。

③　　治験計画書に従い治験が実施できる患者として設定した。

④⑤　被験者からの同意取得を原則とするが、年齢を勘案し、代諾者のみからの取得でも治験参加を可とするため、設定した。

⑥　　治験薬が内服用錠剤であるため設定した。

## 5.2. 除外基準

① 投与開始前 28日以内に、塩酸メクリジンを含有する薬剤を服用した患者

② 投与開始前 28日以内に、骨延長術を実施した患者または治験期間中に骨延長術の実施を予定している患者

③ 重篤な合併症を有する患者

④ 体重11kg未満の患者

⑤ 排尿困難の症状がある患者

⑥ 緑内障の診断を受けた患者

⑦ 塩酸メクリジンに対するアレルギーを有する患者

⑧ かぜ薬, 解熱鎮痛薬, 鎮静薬, 鎮咳去痰薬, 抗ヒスタミン剤を含有する内服薬を常用している患者

⑨ その他、治験責任医師又は治験分担医師が本治験を実施するにあたり、不適当と判断した患者

［設定根拠］

① 　薬剤の安全性、薬物動態を適切に評価するため、設定した。

②~⑨　被験者の安全性を確保するために設定した。

# 6 登録

## 6.1. 登録の手順

以下の手続きに従って、登録を行う。12.5mg投与群及び25mg投与群ともに、目標症例数に到達した場合は、それ以降、到達したコホートへの症例登録は行わない。

治験責任医師又は治験分担医師は、本治験の対象として適格となる可能性があると判断した被験者及びその代諾者から文書による同意を取得し、スクリーニング検査を実施した後、登録基準を満たした場合には、登録・適格確認に必要項目をすべて入力する。登録センターにおいて、治験責任医師又は治験分担医師の入力情報を基に適格性が判定される。入力内容に疑義がある場合、登録センターは直ちに、治験責任医師又は治験分担医師に確認を行う。

登録基準を満たした場合には、登録センターは、治験責任医師又は治験分担医師に連絡する。

登録基準を満たさなかった場合には、治験責任医師又は治験分担医師は、その理由を原資料に記録する。

## 6.2. 登録に際しての注意事項

本治験は第１相試験であり、塩酸メクリジンの効果は期待できず、副作用などの不利益のみが生じる可能性があることを被験者及びその代諾者が理解できない場合、被験者の登録は許容できない。

登録に必要な項目の入力が不十分なときは、すべて満たされるまで登録は受け付けられない。

一度登録された被験者の登録の取り消しは行わない。

誤登録・重複登録が判明した場合には、速やかに登録センターに連絡をする。

重複登録がなされた場合は、先の登録を優先する。

## 6.3. 本治験の被験者として不適格と判断された場合

治験責任医師又は治験分担医師は、被験者として不適格と判断した場合には、必要に応じて適切な治療を行う。同意取得後、登録に至らなかった被験者については、当該被験者の症例報告書を作成しない。

# 7. 治験の方法

## 7.1. 用法・用量

**7.1.1. 治験薬の投与**

治験薬の用法・用量は、以下の通りである。

・ 治験薬1日1回1錠または2錠を夕食摂取終了後1時間に服用する。初回投与及び14回投与は医療機関にて服用とし、朝食摂取終了後1時間に服用する。

服用の1時間前に食事を摂取終了する。治験薬は、一定量の水（150 mL程度）とともに噛まずに服用する。服用の許容範囲は±30分とする。

ただし1日を通して水分摂取量の制限はしない。

食事摂取終了時刻、治験薬投与時刻について原資料へ記録する。

【設定根拠】

軟骨無形成症患者（小児）に対する塩酸メクリジン単回投与の結果より、25mg投与では血漿中メクリジン濃度が成人で報告されているレベルよりも高くなること、摂食後の投与では絶食時の投与と比較し暴露が高くなること、バラツキが大きい事が判明した。よって今回の投与量は1回あたり基本投与量を12.5mgとし、12.5mg投与群の安全性情報を確認後25mg投与群に投与を開始する事とした。また、入院を治験全期間としない本治験では塩酸メクリジンの薬理作用の一つでもある眠気に対する配慮が必要であり、入眠前の夜投与が適していると考えた。

以上から、軟骨無形成症患者（小児）に対し、塩酸メクリジン12.5mgを夕食摂取後に1日1回1錠または2錠14日間反復投与する【用法・用量】とすることは適切と考えた。初回及び14回投与後には薬物濃度測定のため複数回採血を行うため、医療機関にて服用とし、朝の投与とする。14回投与時は前日夜の投与時刻より血中濃度半減期に相当する8.5時間以上経過していることを確認した後投与する。

## 7.2. 各被験者の治験期間

同意取得時より中止時又は最終観察時（最終投与7日後）までを個々の被験者の治験期間とする。

## 治験終了後の治験薬の使用

治験を終了した被験者又は治験を中止した被験者に対して、本治験薬を提供しない。

## 併用薬・併用療法に関する規定

**7.4.1. 併用禁止薬・併用禁止療法**

治験薬投与開始28日前から評価期間終了までの期間、次の薬剤の併用を禁止とする。

① FGFR3シグナルを抑制する成績が発表されている薬剤 （国内未承認の CNP アナログ製剤、スタチン製剤等）

② 塩酸メクリジンを含有する薬剤

治験薬投与開始24時間前から投与終了後24時間以内は、次の薬剤の併用を禁止とする。

③　 かぜ薬, 解熱鎮痛薬, 鎮静薬, 鎮咳去痰薬, 抗ヒスタミン剤を含有する内服薬

（鼻炎用内服薬, アレルギー用薬等）

同意取得後から評価期間終了までの期間において、以下の治療を禁止する。

④ 同意取得時に成長ホルモン製剤による治療を受けている被験者は、評価期間終了　までに治療の中止、変更を禁止する。

⑤　 骨延長術による治療は禁止する。

被験者の治療のため、これらの薬剤、療法を実施する場合は、治験を中止する。

【設定根拠】

①②FGFR3シグナルの過剰抑制を防ぐために設定した。

②薬物動態の評価を適切に行うため設定した。

③ソラシドンの添付文書の記載に従った。

④有効性の評価は実施しないので、成長ホルモン剤の使用は禁止しない。有害事象の観察・評価を正確に行うため、治験期間中の治療の中止、変更は禁止した。

⑤有害事象の観察・評価を正確に行うため治験期間中は禁止した。

**7.4.2. 併用薬・併用療法の記録**

治験責任医師又は治験分担医師又は治験協力者は、同意取得後から評価期間終了までの期間に併用した薬剤について、以下の内容を記録する。ただし、注射剤の溶解等を目的とした薬剤、補液、採血ルート確保のための薬剤及び消毒薬は記録不要とする。

併用薬：薬剤名（商品名）、投与期間、投与量、使用目的

なお、併用療法については、同意取得後から評価期間終了までのうち治療開始から最終評価の期間に併用した治療に関し、療法名、治療期間、治療目的について記録する。

# 8. 評価項目

## 8.1. 主要評価項目

・ 初回投与から21日間の安全性

問診、視診、触診や臨床検査等により、 初回投与から評価終了までのすべての有害事象の事象名、程度、発現割合

- 薬物動態：メクリジンの血漿中薬物濃度パラメータ （全ての症例を対象に、塩酸メクリジン投与前後の血漿中薬物濃度の測定を行う。）

① Cmax (初回及び14回投与後各々)

② Tmax (初回及び14回投与後各々)

③ t 1/2 (14回投与後)

④ AUC (初回及び14回投与後各々)

の算出を行う

［主要評価項目の設定根拠］

塩酸メクリジンを現在の科学水準にて小児に連続投与した記録は無く、その安全性及び薬物動態を評価することが主な目的であるため、安全性、薬物動態を主要評価項目とした。軟骨無形成症患者（小児）でのメクリジンのT1/2は8.5時間であり、17半減期に相当する最終投与7日後では99.999%以上が体内から消去されており評価期間は妥当と考えた。

# 9. 観察及び検査項目及び実施時期

**＜表１　評価スケジュール＞**

＜表２　薬物濃度測定用採血スケジュール＞

## 9.1. 観察及び検査項目

**9.1.1. スクリーニング（同意取得後～登録）**

スクリーニング期間の日数は同意取得後から28日以内とし、スクリーニング期間に以下の検査・観察を実施する。

1. 被験者背景：性別、生年月日
2. 身長
3. 座高
4. 体重
5. 眼科学的検査：細隙灯検査を実施する
6. 既往歴、合併症
7. バイタルサイン（血圧、脈拍数、体温）: 仰臥位で測定する。
8. 12誘導心電図
9. 胸部レントゲン撮影（正面）
10. 血液学的検査：赤血球数、白血球数、血小板数、ヘモグロビン量、ヘマトクリット値
11. 血液生化学検査： 総蛋白(TP)､アルブミン(Alb)、尿素窒素(BUN)､クレアチニン(Cre)､C反応性タンパク(CRP)、ナトリウム(Na)、カリウム(K)、クロール(Cl)、アラニンアミノ基転移酵素(ALT)、アスパラギン酸アミノ基転移酵素(AST)､トリグリセライド(TG)
12. 尿検査：pH、蛋白、グルコース、ウロビリノーゲン、潜血
13. 併用薬、併用療法の確認

「（10）血液学的検査」「（11）血液生化学検査」「（12）尿検査」については、投与開始前28日以内に日常診療として検査している場合は、既存資料としての使用について被験者の同意が得られた場合、当該データをスクリーニング時のデータとして使用する。

「（6）既往歴、合併症」について、既往歴は同意取得の1年前から投与開始までに治癒した疾患、合併症は投与開始日に合併している疾患とする。

**9.1.2.** **治験薬投与開始日-Day 1（投与前の項目）**

1. 体重
2. バイタルサイン（血圧、脈拍数、体温）: 仰臥位で測定する。
3. 12誘導心電図
4. 血液学的検査：赤血球数、白血球数、血小板数、ヘモグロビン量、ヘマトクリット値
5. 血液生化学検査： TP､Alb、BUN､Cre､CRP、Na、K、Cl、ALT、AST､TG
6. 尿検査：pH、蛋白、グルコース、ウロビリノーゲン、潜血
7. 薬物濃度測定
8. 併用薬、併用療法の確認

「(3) 12誘導心電図」はスクリーニング検査を治験薬投与日の前14日以内に実施している場合には、そのデータを投与前のデータとして採用する。

なお、薬物動態評価のための検体の採取、取扱い及び搬送手順については、別途手順書を参照することとする。また、「9.1.3.治験薬投与開始日」～「9.1.8 治験薬投与終了7日後-Day 21（評価期間終了時）」において、薬物動態測定の為の採血時刻について原資料へ記録する。採血時間、心電図測定時間の許容範囲は「9.1.10.時間の許容範囲」を参照する。

**9.1.3. 治験薬投与開始日（投与及び1、２、３、４、６時間後）**

服薬

1. バイタルサイン（血圧、脈拍数、体温）: 仰臥位で測定する。
2. 薬物濃度測定
3. 有害事象
4. 併用薬、併用療法の確認
5. 12誘導心電図：投与3時間後のみ

**9.1.4. 治験薬投与８日目-Day 8（10:00-15:00の間に来院）**

1. バイタルサイン（血圧、脈拍数、体温）: 仰臥位で測定する。
2. 12誘導心電図
3. 血液学的検査：赤血球数、白血球数、血小板数、ヘモグロビン量、ヘマトクリット値
4. 血液生化学検査： TP､Alb、BUN､Cre､CRP、Na、K、Cl、ALT、AST､TG
5. 尿検査：pH、蛋白、グルコース、ウロビリノーゲン、潜血
6. 薬物濃度測定
7. 有害事象
8. 併用薬、併用療法の確認

**9.1.5. 2週後-Day 14（投与前）**

1. 体重
2. バイタルサイン（血圧、脈拍数、体温）: 仰臥位で測定する。
3. 血液学的検査：赤血球数、白血球数、血小板数、ヘモグロビン量、ヘマトクリット値
4. 血液生化学検査： TP､Alb、BUN､Cre､CRP、Na、K、Cl、ALT、AST､TG
5. 尿検査：pH、蛋白、グルコース、ウロビリノーゲン、潜血
6. 薬物濃度測定
7. 有害事象
8. 併用薬、併用療法の確認

**9.1.6. Day14（投与及び1、２、３、４、６、１０時間後）**

服薬

1. バイタルサイン（血圧、脈拍数、体温）: 仰臥位で測定する。
2. 薬物濃度測定
3. 有害事象
4. 併用薬、併用療法の確認
5. 12誘導心電図：投与3時間後のみ

**9.1.7. Day 15（投与24時間後）**

1. バイタルサイン（血圧、脈拍数、体温）: 仰臥位で測定する。
2. 12誘導心電図
3. 薬物濃度測定
4. 有害事象
5. 併用薬、併用療法の確認

**9.1.8. 治験薬投与終了7日後-Day 21（評価期間終了時）**

1. 体重
2. 眼科学的検査：細隙灯検査を実施する
3. バイタルサイン（血圧、脈拍数、体温）: 仰臥位で測定する。
4. 12誘導心電図
5. 血液学的検査：赤血球数、白血球数、血小板数、ヘモグロビン量、ヘマトクリット値
6. 血液生化学検査： TP､Alb、BUN､Cre､CRP、Na、K、Cl、ALT、AST､TG
7. 尿検査：pH、蛋白、グルコース、ウロビリノーゲン、潜血
8. 薬物濃度測定
9. 有害事象
10. 併用薬、併用療法の確認

**9.1.9. 中止時**

中止時の扱い：「10.中止基準」で述べるような状況の場合、治験を中止する。

治験薬投与後に中止する際には、中止決定から7日以内に下記の中止時検査を行う。

1. 体重
2. 眼科学的検査：細隙灯検査を実施する
3. バイタルサイン（血圧、脈拍数、体温）: 仰臥位で測定する。
4. 12誘導心電図
5. 血液学的検査：赤血球数、白血球数、血小板数、ヘモグロビン量、ヘマトクリット値
6. 血液生化学検査： TP､Alb、BUN､Cre､CRP、Na、K、Cl、ALT、AST､TG
7. 尿検査：pH、蛋白、グルコース、ウロビリノーゲン、潜血
8. 薬物濃度測定
9. 有害事象
10. 併用薬、併用療法の確認

**9.1.10. 時間の許容範囲**

薬物濃度測定用採血

初回投与日（投与前） ：初回投与前 60 分のずれを許容する。

初回投与日（投与 1 時間後） ：前後 10 分のずれを許容する。

初回投与日（投与 2 時間後） ：前後 10 分のずれを許容する。

初回投与日（投与 3 時間後） ：前後 10 分のずれを許容する。

初回投与日（投与 4 時間後） ：前後 10 分のずれを許容する。

初回投与日（投与 6 時間後） ：前後 30 分のずれを許容する。

投与8日目来院日 ：ずれは規定せず、採血時刻の記録を行う。

14回投与日（投与前） ：14回投与前 60 分のずれを許容する。

14回投与日（投与 1 時間後） ：前後 10 分のずれを許容する。

14回投与日（投与 2 時間後） ：前後 10 分のずれを許容する。

14回投与日（投与 3 時間後） ：前後 10 分のずれを許容する。

14回投与日（投与 4 時間後） ：前後 10 分のずれを許容する。

14回投与日（投与 6 時間後） ：前後 30 分のずれを許容する。

14回投与日（投与 10 時間後） ：前後 30 分のずれを許容する。

14回投与 2 日目（投与 24 時間後） ：前後 30 分のずれを許容する。

14回投与終了 1 週間後 ：前後 1 日のずれを許容する。

12誘導心電図

投与 3 時間後の採血から4時間後の採血の間に測定をする。

**9.1.11. 検体採取量**

1） 臨床検査：各治験実施医療機関で適宜規定

2） 薬物濃度測定：全血約2 mL

## 9.2. 観察・検査方法

**9.2.1. 有害事象の確認**

問診、視診、触診や超音波検査、X線検査、その他の臨床検査等により、有害事象の発生の有無を確認する。

**9.2.2. バイタルサイン・臨床検査値の確認**

評価スケジュールに規定された時点（その他、必要に応じ適宜実施）のバイタルサイン・臨床検査値を確認する。

# 10. 中止基準

治験責任医師又は治験分担医師は、治験期間を通じて、被験者への治験薬の投与又は被験者の治験参加中止を決定することができる。同様に被験者は、いつでも、いかなる理由でも、自らの意思で治験薬の投与中止又は治験の中止を希望することができる。

以下の中止基準に該当する場合は、治験を中止する。

1. 被験者又は代諾者の自由意思による同意撤回の申し入れがあった場合
2. 治験責任医師又は治験分担医師により、原疾患あるいは合併症の悪化又は重篤な有害事象等の発現のため治験の継続が困難と判断された場合
3. GCP 違反、選択基準違反又は除外基準抵触等、治験実施計画書からの重大な逸脱が判明した場合
4. 治験実施計画書の遵守が不可能になった場合
5. 治験期間中に薬物動態・蓄積性又は安全性の評価の上で、対象として不適切であることが判明した場合
6. その他、治験責任医師又は治験分担医師により治験の継続が困難と判断された場合

【設定根拠】

(1)～(6)　治験を倫理的に実施するため設定した。

# 11. 有害事象

## 11.1. 有害事象発現時の被験者への対応、並びに有害事象の調査と報告

治験責任医師又は治験分担医師は、被験者に有害事象が認められたときは、直ちに適切な処置を行い、有害事象に対する医療行為が必要となった場合には被験者にその旨を伝える。また、治験薬投与開始日以降に認められた有害事象の症状又は疾患、他覚所見の内容、発現日、重症度、重篤な事象と判断した場合はその判断理由、処置の有無及びその内容、転帰及びその判定日、投与された治験薬との関連性及び因果関係がある場合はその理由を記録する。ただし、重篤な有害事象の場合は、因果関係がある場合も無い場合もその判断理由を記録する。

原則として被験者等の死亡、転院等により調査不能となった場合を除いて、正常化又は有害事象として捉えないレベルに回復するまで追跡調査を行う。また、器質的な障害（脳梗塞・心筋梗塞等）で不可逆的な有害事象が認められた場合は、症状が安定するまで追跡調査を行うこととする。ただし、重篤な有害事象については、治験責任医師又は治験分担医師が有害事象として捉えないレベルに回復、器質的な障害が症状安定と判断した場合は、その判断した根拠を記録するものとする。

治験薬投与開始以降に、投与された治験薬との因果関係の有無にかかわらず重篤な有害事象が発現した場合、治験責任医師又は治験分担医師は、被験者等に対して直ちに適切な処置・対処を行い、また、「安全性情報の取扱いに関する手順書」に従い、治験責任医師は、速やかに所属機関の長、治験調整医師及び治験薬提供者に報告し、治験調整医師は必要に応じて厚生労働大臣にも報告する。

なお、治験責任医師は、毎年一回、治験の進捗状況等を所属機関の長に報告する。

## 11.2. 有害事象等の定義

1） 有害事象

有害事象とは、治験薬を投与した被験者に生じたすべての疾病若しくは障害又はこれらの兆候であり（臨床検査値の異常変動を含む）、治験薬との因果関係の有無を問わない。本治験では、治験薬の投与開始から評価期間終了／中止時までに発現したあらゆる好ましくない医療上のできごとを有害事象として取り扱う。

2） 有害事象の程度の分類

被験者に発現した有害事象はCTCAE v5.0/ MedDRA/J v23.1で分類、集計する。

Gradeは有害事象の重症度を意味する。CTCAEではGrade 1～5を以下の表の原則に従って定義されており、各有害事象の重症度の説明が個別に記載されている。本治験では、合併症について、CTCAE ver. 5.0で1段階以上グレードが悪化した場合を有害事象と判定する。臨床検査値の異常を同様に判断する。

**＜表２　CTCAE ver. 5.0によるGrade＞**

| Grade | 説明文^a）、b）^ |
| --- | --- |
| Grade 1 | 軽症；症状がない、又は軽度の症状がある；臨床所見又は検査所見のみ；治療を要さない |
| Grade 2 | 中等症；最小限/局所的/非侵襲的治療を要する；年齢相応の身の回り以外の日常生活動作の制限^c）^ |
| Grade 3 | 重症又は医学的に重大であるが、ただちに生命を脅かすものではない；入院又は入院期間の延長を要する；身の回りの日常生活動作の制限^d）^ |
| Grade 4 | 生命を脅かす；緊急処置を要する |
| Grade 5^e）^ | 有害事象による死亡 |

a） 説明文中のセミコロン（；）は「又は」を意味する

b） 総ての有害事象が総てのGradeを含むわけではないので、一部の有害事象ではGradeの選択肢が5種類未満となっている

c） 身の回り以外の日常生活動作とは、食事の準備、日用品や衣服の買い物、電話の使用、金銭の管理等をさす

d） 身の回りの日常生活動作とは入浴、着衣・脱衣、食事の摂取、トイレの使用、薬の内服が可能で、寝たきりではない状態をさす

e） 一部の有害事象にはGrade 5（死亡）が該当しないため選択肢に含めていない

3） 有害事象の転帰の分類

有害事象の転帰は、以下の基準で分類する。

① 回復

② 軽快

③ 回復したが後遺症あり

④ 未回復

⑤ 死亡

⑥ 不明

4） 有害事象の因果関係の評価

治験責任医師又は治験分担医師は、治験薬に当該有害事象を引き起こす「合理的な可能性」が存在するか否かを評価する。評価は、原疾患、合併症等の基礎疾患の自然経過、併用療法、その他の危険因子等、治験薬以外の原因及び治験薬使用と事象発現との時間的関連を勘案し、次の様に分類する。

1. 関連あり
2. 関連なし

5） 有害事象の予測可能性

本治験では、治験薬に関連する有害事象として、当該事象等の発生、あるいは発生数、発生頻度、発生条件等の発生傾向が本治験薬概要書あるいは下記から予測できないものを「未知」、予測できるものを「既知」とする。ただし、厚生労働大臣へ報告した安全性情報を治験薬概要書の別冊として取扱い、当該報告文書の作成日をもって当該事象を「既知」と取扱う。

6） 重篤な有害事象

重篤な有害事象とは、有害事象のうち、以下のいずれかに該当するものとする。

① 死亡

② 死亡につながるおそれのあるもの

③ 治療のために入院又は入院期間の延長が必要となるもの（ただし、以下の理由による入院は重篤な有害事象とみなさない）

• 治験実施計画書に規定された計画的な入院

• 治験開始前から予定されていた入院、又は治験期間中に実施することが予定された入院

• 検査等のための入院（有害事象を伴わない）

④ 永続的もしくは重大な障害・機能不全に陥るもの

⑤ 障害につながるおそれのあるもの

⑥ 上記①～⑤に掲げる症例に準じて重篤であるもの

⑦ 後世代における先天性の疾病又は異常等

## 11.3. 重篤な有害事象が発現した場合の対応

治験薬投与開始以降に被験者に重篤な有害事象が発現した場合、治験調整医師、治験責任医師及び所属機関の長は、「安全性情報の取扱いに関する手順書」に従い、以下の事項を実施する。

① 治験責任医師及び治験分担医師は、因果関係の有無にかかわらず、直ちに被験者の安全確保に必要な措置を行うとともに、治験責任医師は、所属機関の長、治験調整医師及び治験薬提供者に報告を行う。当該事象の発現を知ってから、原則として24時間程度以内を目安に重篤な有害事象に関する報告書により、その発現経緯及び被験者の症状について、所属機関の長及び治験調整医師に報告しなければならない。また、治験分担医師にも、その発現経緯及び被験者等の症状について、情報を提供する。

② 治験責任医師は、当該有害事象に関する追加情報が得られた場合には、重篤な有害事象に関する報告書により、可能な限り速やかに所属機関の長及び治験調整医師に追加報告を行うとともに、治験分担医師に情報を提供する。

③ 治験調整医師は、医薬品、医療機器等の品質、有効性及び安全性の確保等に関する法律施行規則第273条の2第1項に規定される報告対象に該当し、厚生労働大臣への報告が必要と判断した場合は、厚生労働大臣に報告する。

## 11.4. 本治験において予測される有害事象等

予測される有害事象等

ソラシドンの添付文書に記載がある有害事象の症状は以下のとおりである。

皮膚：発疹・発赤, かゆみ

泌尿器： 排尿困難

その他：口のかわき, 眠気

また、単回投与第1相試験である“軟骨無形成症患者（小児）に対する塩酸メクリジン製剤の安全性及び薬物動態の検討”(CAMCR-009)の試験計画書に記載がある同一有効成分の一般用医薬品センパアＳ（大正製薬：現在は製造中止）の有害事象の症状は以下のとおりである。

皮膚：発疹・発赤，かゆみ

精神神経系：頭痛

泌尿器：排尿困難

その他：顔のほてり、異常なまぶしさ、口のかわき、便秘、眠気、目のかすみ

これらの症状の出現、並びにこれらの症状を伴う疾患については「既知」とする。

## 11.5. 有害事象記録手順

治験責任医師又は治験分担医師は、有害事象を記録する際は、適切な医学用語・概念を用いるよう努め、口語表現や略語の使用は避ける。記録の事象欄には、有害事象名を1つだけ入力する。

**11.5.1. 診断と徴候・症状**

有害事象については、個々の徴候及び症状ではなく診断（特定されている場合）を記録する（例：黄疸やトランスアミナーゼ上昇ではなく、肝不全又は肝炎を記録する）。しかし、報告時点で一連の徴候や症状が単一の診断又は症候群として医学的に特定できない場合、それぞれ個別の事象を記録する。後に診断が確定した場合、徴候及び症状に基づいてそれまでに報告された総ての有害事象を無効にし、最終的な診断の最初の症状の開始日を発現日とし、単一の診断に基づいた1つの有害事象報告と差し替える。

**11.5.2. 他の事象に続発した有害事象**

原則として他の事象に続発した有害事象（連続的に発現する有害事象や臨床経過で続発した症状等）については、続発した事象が高度又は重篤な有害事象である場合を除き、その主な原因となった有害事象のみを記載する。ただし、原因となった事象から時間的間隔をおいて続発した医学的に重大な有害事象は、独立した事象として記録する。以下に具体例を記す。なお、有害事象間の関連の有無が不明な場合は、総ての事象を別々に記録する。

・ 嘔吐を発現し、治療を要しない軽度の脱水をきたした場合は、嘔吐のみを記録する。

・ 嘔吐の結果、高度の脱水をきたした場合は、両方の事象を別々に記録する。

・ 高度の胃腸出血によって腎不全をきたした場合は、両方の事象を別々に記録する。

・ 好中球減少症に伴って軽度の非重篤な感染症が発現した場合は、好中球減少症のみを記録する。

・ 好中球減少症に伴って高度又は重篤な感染症が発現した場合は、両方の事象を別々に記録する。

**11.5.3. 持続性又は再発性の有害事象**

持続性の有害事象とは、複数の評価時点をまたいで回復せずに継続的に認められる有害事象を指す。これらの事象は、1回だけ記録し、事象の重症度については発症時点での重症度を記録する。事象が悪化した場合は常に最も高い重症度が反映されるように重症度を更新する。事象が重篤化した場合は、それが反映されるように記録を更新する。

再発性の有害事象とは、評価時点間に消失し、その後再発する有害事象を指す。再発した場合は、事象のそれぞれを記録する。

**11.5.4. 臨床検査値異常**

総ての臨床検査値異常が有害事象に該当するわけではなく、以下のいずれかの基準を満たす臨床検査結果を有害事象として記録する。

・ 臨床症状を伴うもの

・ 治験薬に関する変更（投与中止等）を要するもの

・ 医学的介入（低カリウム血症に対するカリウム補給等）を要するもの

・ 治験責任医師又は治験分担医師により臨床的に重要な所見であると判断されるもの

治験責任医師又は治験分担医師は総ての臨床検査所見を確認する責任を負う。単独の臨床検査値異常を有害事象として分類すべきかどうかの判断は、医学的かつ科学的な根拠に基づいて行う。

臨床的に重要な臨床検査値異常が疾患又は症候群の徴候である場合（例：胆嚢炎に伴って、ALPとビリルビン値が正常値上限の5倍を超えた場合等）は、診断名（例：胆嚢炎）のみを記録する。臨床的に重要な臨床検査値異常が疾患又は症候群の徴候でない場合は、異常値そのものを記録する。入力にあたっては、その検査結果が正常値を上回っているか、下回っているのかが明らかになるように入力する（例：「ALPの異常」ではなく、「ALP増加」等と入力する）。また、臨床検査値異常が一般的な定義に準じた的確な臨床用語によって表現できる場合は、その臨床用語を有害事象として記録する（例：血清カリウム値の上昇（7.0 mEq/L）は、「高カリウム血症」と入力）。

複数の来院日にわたって同一の臨床的に重要な臨床検査値異常が認められた場合は、その病因に変化がない限り、繰り返し記録する必要はない。事象の最初の重症度を入力し、その事象が悪化した際に重症度又は重篤度を更新する。

**11.5.5. バイタルサイン異常**

総てのバイタルサイン異常が有害事象に該当するわけではなく、以下のいずれかの基準を満たすバイタルサイン測定結果を有害事象として記録する。

・ 臨床症状を伴うもの

・ 治験薬に関する変更（投与中止等）を要するもの

・ 医学的介入を要するもの

・ 治験責任医師又は治験分担医師により臨床的に重要な所見であると判断されるもの

治験責任医師又は治験分担医師は総てのバイタルサイン所見を確認する責任を負う。単独のバイタルサイン異常を有害事象として分類すべきかどうかの判断は、医学的かつ科学的な根拠に基づいて行う。

臨床的に重要なバイタルサイン異常が疾患又は症候群の徴候である場合（例：血圧上昇）は、診断名（例：高血圧）のみを記録する。

前回の来院時と同一の臨床上重大なバイタルサイン異常が認められた場合、原因が異なる場合を除き、繰り返し記録しない。事象の最初の重症度を入力し、その事象が悪化した際に重症度又は重篤度を更新する。

## 11.6. 新たな情報の提供

治験責任医師は、治験調整医師等から本治験に関連した措置報告や研究報告等、安全性に関する新たな情報を得た場合には、速やかに所属機関の長に文書で報告し、治験実施関係者に周知させる。被験者の治験継続の意思に影響を与える重大な事項である場合は、速やかに被験者へ追加説明し、必要に応じて説明文書･同意文書の改訂を行った上で、再同意を取得する（「17.6.2. 再同意の取得」の項の手順参照）。

また、治験責任医師は、治験薬による治療に関し、被験者の生命、健康の安全に直接係わる危険情報の収集に努め、医薬品、医療機器等の品質、有効性及び安全性の確保等に関する法律施行規則第275条の3に規定する事項を知り得た場合は、その内容に応じて厚生労働大臣に報告する。

# 12. 効果安全性評価委員会

本治験では、有識者からなる効果安全性評価委員会を組織する。効果安全性評価委員には本治験の関係者は含まない。効果安全性評価委員会の評価手順については、別途作成する手順書に定める。

# 13. 治験の終了、中止･中断

## 13.1. 治験の終了

治験調整医師は、目標とする症例数が登録され、総ての被験者についての評価が終了し、総ての記録の内容を確認したことを以て治験終了とする。また、治験責任医師は、治験実施医療機関で実施した総ての症例の記録の内容を確認し、署名したことを以て治験終了とし治験終了報告書を所属機関の長に提出する。

## 13.2. 治験全体の中止･中断

治験調整医師は、以下の事項に該当する場合は治験実施継続の可否を検討する。本治験の中止を決定した時は、速やかに、治験責任医師及び規制当局にその理由とともにその旨を文書で報告する。また、本治験を中止した場合は、登録センターに中止を連絡する。治験責任医師は、速やかに中止する旨及びその理由を所属機関の長に文書により通知する。

1） 治験の品質に関する重大な情報が得られた場合

2） 論文や学会発表等、本治験以外から得られた関連情報を評価した結果、本治験の安全性に問題があると判断された場合、又は治験継続の意義がなくなったと判断された場合

3） 被験者のリクルートが困難で目標症例数の組入れが困難であると判断した場合

4） 本治験における有害事象の発現率又は重症度から、被験者安全性に問題があると判断された場合

5） 治験調整医師が中止と判断した場合

6） 効果安全性評価委員会が本治験を評価した結果、本治験の安全性に問題があると判断された場合

7） 実施医療機関が医薬品GCP省令又は治験実施計画書に違反することによる適正な治験に支障を及ぼしたと認められる場合

8) 治験実施計画書の変更が必要となり、実施医療機関がこれに対応できない場合

9) 治験審査委員会の意見に基づく実施医療機関の長からの治験実施計画書等に対する修正の指示があり、治験調整医師がこれを承諾できない場合

10) 治験審査委員会の判断に基づき、実施医療機関の長が治験の中止を指示した場合

# 14. 目標症例数と治験期間

## 14.1. 目標症例数

1群6例　2群構成　計12 例

［設定根拠］

本治験の主目的は、塩酸メクリジンの安全性及び薬物動態を確認することであるため、統計学的根拠に基づく症例数設計は行わない。

類似の治験にて多く用いられる症例数として、目標症例数は、1群6例とする。

## 14.2. 治験期間

治験全体の実施期間：2021年3月～2022年3月

症例登録期間：2021年4月～2022年1月

被験者の治験期間：7週間程度（スクリーニング期間4週程度+投与期間2週間+フォローアップ期間1週間）

［設定根拠］

軟骨無形成症患者（小児）でのメクリジンのT1/2は8.5時間であり、7日間のフォローアップを目安として安全性を確認する事とした。

# 15. 統計的事項

## 15.1. 解析対象集団

主要評価項目のうち安全性の解析は安全性解析対象集団、薬物動態の解析は最大の解析対象集団（Full Analysis Set ：FAS）を主たる解析対象集団とする。また、本治験実施計画書に適合した解析集団（Per Protocol Set ：PPS）を対象とした解析も実施し、解析結果の頑健性を確認する。

1） 安全性解析対象集団：本治験に登録され、治験薬が1回以上投与された被験者。

2） 最大の解析対象集団（FAS）：安全性解析対象集団から、重大な治験実施計画書違反（同意未取得、治験手続き上の重大な違反）の被験者及び投与後のデータが全くない被験者を除いた被験者。

3） 治験実施計画書に適合した対象集団（PPS）：FASのうち、治験実施計画書の規定に対して、以下の違反があった被験者を除いた被験者。

① 選択基準・除外基準違反（「5. 対象患者及び適格性の基準」の項参照）

② 併用禁止薬・併用禁止療法違反（「7.4.1. 併用禁止薬・併用禁止療法」の項参照）

## 15.2. 解析項目･方法

主要評価項目の解析の詳細は統計解析計画書で規定する。

**15.2.1. 主要評価項目**

1） 初回投与から21日間の有害事象

有害事象はCTCAE v5.0/ MedDRA/J v23.1で分類、集計する。

初回投与から評価終了までに認められた総ての有害事象の発現率及び95%信頼区間を算出する。95%信頼区間はClopper-Pearson 法を用いて算出する。また、有害事象を器官大分類別、基本語別、重症度別及び試験薬との関連性別に発現率を算出する。重篤な有害事象も同様に算出する。重篤な有害事象は被験者別に一覧表に示す。

2） 薬物動態

メクリジンの血漿中薬物濃度パラメータ （全ての症例を対象に、塩酸メクリジン投与前後の血漿中薬物濃度の測定を行う。）

① Cmax (初回及び14回投与後各々)

② Tmax (初回及び14回投与後各々)

③ t 1/2 (14回投与後)

④ AUC (初回及び14回投与後各々)

## 15.3. 統計解析計画書の変更

統計解析計画をデータ固定前に変更する場合は、その変更理由を統計解析計画書及び総括報告書に記述する。データ固定後に解析方法の変更又は追加解析を実施する場合は、その理由を解析報告書及び総括報告書に記述し、計画されていた解析結果と区別する。

## 15.4. 最終解析

観察期間終了後、データを確定した後にすべての評価項目に対する解析を統計解析計画書に従い行う。最終解析結果は「最終解析報告書」としてまとめ、 治験調整医師及び治験調整事務局は最終解析報告書の内容を総括し、治験全体の結論、問題点、結果の解釈と考察等をまとめた「総括報告書」を作成する。

# 16. 品質管理及び品質保証

治験調整医師は、本治験の品質及び信頼性維持のために、別途作成する手順書に基づく「治験の品質管理」及び「治験の品質保証」を行わなければならない。また、実施医療機関は、治験責任医師による治験の品質管理及び品質保証に協力しなければならない。

治験の品質管理においては、モニタリング担当者は、「モニタリングの実施に関する手順書」に従い、本治験が実施医療機関の治験に係わる業務に関する手順書、最新の治験実施計画書及びGCPを遵守して実施されていることを確認する。

また、治験責任医師又は治験分担医師から報告されたCRFの内容が正確かつ完全であることを、原資料等の治験関連記録に照らして検証できることを確認する。

また、治験が治験実施計画書及びGCPを遵守して行われていることを保証するため、監査担当者は手順書に従って監査を行い、品質管理が適切に行われていることを確認する。

# 17. 倫理的事項

## 17.1. 遵守すべき諸規則

本治験は、ヘルシンキ宣言、医薬品医療機器等法、同施行規則、GCP、治験実施計画書を遵守して実施する。

## 17.2. 治験実施計画書の遵守

本治験に参加する治験責任医師及び治験分担医師は、被験者の安全と人権を損なわない限り、本治験実施計画書を遵守する。

## 17.3. 治験実施計画書等の変更

治験責任医師は、改訂内容の重大性に関わらず、総ての改訂内容とその理由をIRBに報告し、改訂内容とその変更理由等について、再度審査を受け、承認を得る。また、その改訂内容に応じて説明文書・同意文書も改訂する。

## 17.4. 治験実施計画書からの逸脱について

治験責任医師又は治験分担医師は、IRBの事前の審査に基づく所属機関の長の文書による承認を得ることなく、治験実施計画書の内容から逸脱すること、又は治験計画の変更を行ってはならない。ただし、被験者の緊急の危険を回避する等、医療上やむを得ない場合、又は治験の事務的事項のみに関する変更である場合（誤植の訂正、治験実施組織体制の変更、治験実施関係者の所属・職名の変更等）を除く。

被験者の緊急の危険を回避する等、医療上やむを得ない場合、治験責任医師又は治験分担医師は、逸脱又は変更の内容及び理由、並びに治験実施計画書等の改訂が必要であればその案を速やかに、所属機関の長に文書で提出し、IRB及び所属機関の長の承認を得なければならない。

治験責任医師又は治験分担医師は、治験実施計画書からの逸脱があった場合は、逸脱事項をその理由とともに総て記録する。なお、治験責任医師は、これらの記録を保存しなければならない。

## 17.5. 説明文書及び同意文書の作成と改訂

被験者から治験への参加の同意を得るために用いる説明文書、アセント文書及び同意文書（以下、同意説明文書という）は、治験責任医師が作成し、IRB及び所属機関の長の承認を受けたものを使用する。また、治験責任医師は被験者の同意に影響を及ぼすような新たな情報を入手した場合等、同意説明文書を改訂する必要があると判断した場合は、同意説明文書を速やかに改訂し、IRB及び所属機関の長の承認を得る。

被験者の同意に影響を及ぼすと考えられる有効性や安全性等の情報が得られたときや、被験者の同意に影響を及ぼすような実施計画等の変更が行われるときは、速やかに被験者に情報提供し、研究等に参加するか否かについて被験者の意思を予め確認するとともに、事前にIRB及び所属機関の長の承認を得て同意説明文書の改訂を行い、被験者の再同意を得る。

なお、説明文書には、以下の内容を含む事項を記載する。

1. 治験が試験を目的とするものであること
2. 治験の目的
3. 治験責任医師の氏名、職名及び連絡先
4. 治験の方法（治験の試験的側面、被験者の選択基準、参加予定人数）
5. 予測される治験薬による被験者の心身の健康に対する利益（当該利益が見込まれない場合はその旨）及び予測される被験者に対する不利益
6. 他の治療方法に関する事項（他の治療方法の有無及びその治療方法に関して予測される重要な利益及び危険性）
7. 治験に参加する期間
8. 治験の参加をいつでも取りやめることができること
9. 治験に参加しないこと又は参加を取りやめることにより被験者等が不利な取扱いを受けないこと
10. 治験の参加を取りやめる場合の治験薬の取扱いに関する事項
11. 被験者の秘密が保全されることを条件に、モニター、監査担当者、IRB及び規制当局等が原資料を閲覧できること
12. 被験者の秘密は保全されること（治験の結果が公表される場合であっても、被験者の秘密は保全されること）
13. 健康被害が発生した場合における実施医療機関の連絡先
14. 健康被害が発生した場合に必要な治療が行われること
15. 健康被害の補償に関する事項
16. 治験の適否等について調査審議を行うIRBの種類、IRBにおいて審査を行う事項その他当該治験に係るIRBに関する事項
17. 治験に継続して参加するかどうかについて被験者の意思に影響を与えるものと認める情報を入手した場合には直ちに被験者に伝えられること
18. 治験への参加を中止させる場合の条件又は理由
19. 被験者が費用負担をする必要がある場合にはその内容
20. 被験者に金銭等が支払われる場合にはその内容（支払額算定の取決め等）
21. 治験への参加後、治験の参加を取りやめる場合の治験薬の取扱いに関する事項

## 17.6. インフォームド・コンセント

**17.6.1. 説明と同意取得**

1. 治験責任医師又は治験分担医師は治験実施に先立ち、治験審査委員会にて承認を得た最新の同意説明文書を対象となる被験者及びその代諾者に手渡して、十分に説明する。また、治験協力者が補足的に説明することもできる。なお、説明に際し、本治験に関する説明文書に基づき、被験者及びその代諾者が理解できるように可能な限り平易な言葉を用いて説明し、被験者及びその代諾者の質問に対して十分に答えなければならない。被験者及びその代諾者が内容をよく理解したことを確認した上で、本治験への参加について被験者の自由意思による同意をその代諾者から文書にて得る。併せて、被験者が7歳以上においてはアセントを本人から文書にて得る。
2. 同意文書には説明を行った治験責任医師又は治験分担医師及び被験者の代諾者が署名し、各自日時を記載する。併せて、被験者が7歳以上においてはアセント文書には説明を行った治験責任医師又は治験分担医師及び被験者が署名し、各自日時を記載する。いずれの場合も、治験協力者が補足的に説明を行った場合は、当該治験協力者も署名し、日時を記載する。
3. 治験責任医師又は治験分担医師は、被験者の代諾者が説明文書を読むことができない場合、公正な立会人を立ち会わせた上で十分に説明し、被験者の自由意思による同意を得る。また、立会人も同意文書に署名し、日時を記載する。
4. 治験責任医師又は治験分担医師は、被験者が治験に参加する前に、署名と日時が記載された同意文書の写しと説明文書を被験者の代諾者に交付する。併せて、被験者が7歳以上においては署名と日時が記載されたアセント文書の写しと説明文書を被験者の代諾者に交付する。また、同意文書の原本は当該実施医療機関の規定に従って適切に保管する。

**17.6.2. 再同意の取得**

治験参加の継続について、被験者の意思に影響を与える可能性がある情報が得られた場合、治験責任医師又は治験分担医師は、当該情報を速やかに伝え、被験者の治験の継続についての意思を確認し、その結果を診療録に記録する。また、説明文書・同意文書を改訂した場合は、IRBの承認を得た上で、文書にて再同意を得る。

**17.6.3. 被験者が他の医師による治療を受けている場合の通知**

治験責任医師又は治験分担医師は、同意を取得した被験者が他の医師（治験実施医療機関の他科、他の医療機関）から治療を受けているか否かを確認する。被験者が他の医師から治療を受けている場合には、治験責任医師又は治験分担医師は被験者の同意を得た上で、本治験の中止時又は最終観察時まで本治験に参加することをその医師に通知する。また、この通知を行ったことを診療録等に記録する。

## 17.7. 個人情報の保護

被験者の登録及びCRFにおける被験者の特定は被験者識別コード等で行い、被験者の個人情報を保護する。治験成績の公表においては、被験者の氏名、疾患等のプライバシー保護に十分配慮する。本治験に関与する総ての者は、個人情報漏洩のリスクを踏まえた上で、個人情報保護のため最大限の手段を講じる。

本治験の結果は、将来新たな治療法として認可されるために使用されることや医学雑誌等に発表されることがあるが、その際は被験者の名前や身元を特定できる情報を含まないようにする。

1） 匿名化

本治験では、被験者識別コードを用いて被験者のプライバシーと秘密の保全に配慮する。連結可能匿名化とし、その個人情報管理は、治験実施医療機関の規定に従い適切に管理を行う。

2） 開示

本治験成果や安全性評価の結果は、共同実施機関や各学会、学術雑誌及びデータベース上等に発表される可能性はあるが、開示するデータは個人を特定できないものにするよう適切な配慮を十分に行う。本治験への参加により、モニタリング担当者、監査担当者、IRB、厚生労働省や医薬品医療機器総合機構の担当者が、被験者のカルテ開示を求めることがあるが、被験者及びその家族のプライバシーが外部に漏れる心配はないことを、同意説明文書により説明する。また、マスメディア（新聞・テレビ等）の関心を引く可能性はあるが、被験者の許可がない限り、治験関係者は被験者のプライバシーと秘密の保全に配慮する。

# 18. 治験の費用負担

## 18.1. 治験に関する費用、資金源及び財政上の関係

本治験実施に関し、治験調整医師は、治験薬の製造会社、薬物濃度測定機関からの資金提供、労務提供を受けていない。本治験の各検査項目に係る費用、並びに入院に係る費用（入院基本料を除く）は原則として被験者負担（保険診療）で行う。

その他、入院基本料、本治験の運営にかかる費用は総て研究費で負担する。

本治験で起こりうる利益相反については、治験実施医療機関の規定に従い、対応することとする。なお、治験の実施が被験者の権利･利益を損ねることはない。

## 18.2. 健康被害に対する措置

治験に関連して被験者に生じた健康被害に対する補償のため、以下に掲げる事項その他必要な措置を講じておく。補償の内容は、治験薬の適正使用において本治験に起因して生じたとみられる健康被害に対する医療の提供及び治験保険による障害補償金･遺族補償金の支払いとする。なお、別途定める「被験者の健康被害補償に関する手順書」に従い、適切に対応するものとする。

1） 医療の提供

当該健康被害に対し最善の治療を行う。

2） 治験保険による障害補償金･遺族補償金の支払い

障害補償金･遺族補償金の支払いの対象となる事象が発現した場合、治験保険によりこれを支払う。この際、速やかに加入している保険会社に連絡し、必要な対応をとる。本保険では医療費･医療手当も含まれるが、詳細は特約を確認すること。なお、治験薬の製造の過失に起因して被験者に健康被害が生じた場合は、加入する治験薬の生産物賠償責任保険によりこれを支払う。

## 18.3. 被験者に対する金銭の支払い

本治験において被験者に支払われる負担軽減費に関しては、各治験実施医療機関で規定している負担軽減費額を参考に金額及び支払い対象日を決定し、IRB の承認を得る。なお、予定される負担軽減費は名古屋大学の研究費より支払われる。

# 19. 試料等の保存及び使用方法並びに保存期間

## 19.1. 試料等の保存等について

原則として、血液・尿の検査検体は、目的とする検査、投与の終了後、適切に廃棄される。

ただし、 薬物濃度測定用の検体のうち残余検体についてはバイオマーカー探索の臨床研究に使用する。該当臨床研究は倫理審査委員会に申請し、承認を受けた上で実施する。

## 19.2. 記録の保存

本治験から得られた総てのデータ（臨床検査値や画像等）は匿名化され、また、治験等の実施に係わる文書及び記録類（申請書類の控え、所属機関の長からの通知文書、各種申請書･報告書の控え、対応表等、その他データの信頼性を保証するのに必要な書類又は記録等）を鍵のかかるロッカー等に下記の要領で保存する等、治験実施医療機関の規定に従い適切に管理し、その後は個人情報等に注意して廃棄する。また、同意文書は、治験実施医療機関の規定に従い保管し、その後は個人情報等に注意して廃棄する。

1） 治験実施医療機関

実施医療機関の長が定めた記録保管責任者は、実施医療機関において保存すべき治験に係る文書又は記録を次の1) 又は2) のうち遅い日までの期間保存する。ただし、治験責任医師がこれよりも長期間の保存を必要とする場合には、実施医療機関は、保存期間及び保存方法について治験責任医師と協議する。

1. 当該被験薬に係る医薬品についての製造販売の承認を受ける日（当該治験により収集された臨床試験成績に関する資料が承認申請に添付されないことを知り得た日から3年が経過した日）
2. 治験の中止又は終了後3年が経過した日

2） 治験責任医師

治験責任医師は、治験責任医師において保存すべき治験に係る文書又は記録を次の1) 又は2) のうち遅い日までの期間保存する。

1) 当該被験薬に係る医薬品についての製造販売の承認を受ける日（当該治験により収集された臨床試験成績に関する資料が承認申請に添付されないことを知り得た日から3年が経過した日）

2) 治験の中止又は終了後3年が経過した日

3） 治験調整医師

治験調整医師は、治験調整医師において保存すべき治験に係る文書又は記録を次の1) 又は2) のうち遅い日までの期間保存する。

1) 当該被験薬に係る医薬品についての製造販売の承認を受ける日（当該治験により収集された臨床試験成績に関する資料が承認申請に添付されないことを知り得た日から3年が経過した日）

2) 治験の中止又は終了後3年が経過した日

なお、治験調整医師、治験責任医師並びに治験実施医療機関が、塩酸メクリジン製剤の製造販売承認の取得を希望する企業に本治験の成績を、製造販売承認のために承認申請書に添付することを許諾した場合、上記保管期限終了後の保管については当該企業と話し合う。また、当該企業は本治験の成績を使用して製造販売承認が得られた場合、あるいは承認に至らず開発中止を決定した場合には、その旨を治験調整医師に文書で報告する。

# 20. 治験の登録と進捗状況の報告

本治験は実施に先立ち事前に臨床研究実施計画・研究概要公開システム（jRCT）に登録を行い、本治験実施計画の主要なデザインを公開する。

・ jRCT臨床研究実施計画番号：jRCT2041200114

治験責任医師は治験の進捗状況等を1年に1回、IRBに報告する。また、治験を終了したときは、所属機関の長にその旨及び結果の概要を文書により報告する。

# 21. 治験成果の帰属と結果の公表

本治験の成果は、名古屋大学に帰属するものとする。本治験から得られたデータを公表する場合、事前に名古屋大学に提出し、確認を受けなければならない。

治験結果の論文発表に際しては、最終解析終了後に（場合によっては、中間解析終了後にも）、原則として英文誌に投稿する。また、学会等での発表、論文発表に関しては、原則として、治験結果の主たる公表論文の著者は治験調整医師、名古屋大学医学部附属病院先端医療開発部で本治験への貢献度を考慮して、相談の上、決定する。発表の際には被験者の秘密を保全する。

# 22. 治験実施組織・体制

本治験の実施に係る組織と各組織の責任者の所属・氏名・連絡先等は、別紙として示し、変更があった場合でも治験実施計画書の変更とはせず、改訂された別紙を治験実施医療機関へ報告する。

# 23. 参考資料･文献リスト

1. Krejci P, Murakami S, Prochazkova J, et al. NF449 is a novel inhibitor of fibroblast growth factor receptor 3 (FGFR3) signaling active in chondrocytes and multiple myeloma cells. J Biol Chem. 2010;285:20644–20653.
2. Jonquoy A, Mugniery E, Benoist-Lasselin C, et al. A novel tyrosine kinase inhibitor restores chondrocyte differentiation and promotes bone growth in a gain-of-function Fgfr3 mouse model. Hum Mol Genet. 2012;21:841–851.
3. Yasoda A, Komatsu Y, Chusho H, et al. Overexpression of CNP in chondrocytes rescues achondroplasia through a MAPK-dependent pathway. Nat Med. 2004;10:80–86.
4. Lorget F, Kaci N, Peng J, et al. Evaluation of the therapeutic potential of a CNP analog in a Fgfr3 mouse model recapitulating achondroplasia. Am J Hum Genet. 2012;91:1108–1114.
5. Yamashita A, Morioka M, Kishi H, et al. Statin treatment rescues FGFR3 skeletal dysplasia phenotypes. Nature. 2014;513:507–511.
6. Fagan JE, Kaplan BJ, Raymond JE, Edgington ES,, The failure of antimotion sickness medication to improve reading in developmental dyslexia: results of a randomized trial.. J Dev Behav Pediatr. 1988 Dec;9(6):359-66.
7. Kitoh H, Matsushita M et al. Pharmacokinetics and safety after once and twice a day doses of meclizine hydrochloride administered to children with achondroplasia. PLoS One.2020;15(4):e0229639
