# Supplementary material for: Phase 1b study on the repurposing of meclizine hydrochloride for children with achondroplasia
Source: PLoS One. 2023 Jul 10;18(7):e0283425. doi: 10.1371/journal.pone.0283425 (PMC10332602; doi:10.1371/journal.pone.0283425)
Supplement: S1 Table — (DOCX) [file pone.0283425.s004.docx]

| **S1 Table.** Summary of vital signs at each time point | | | |  |  |
| --- | --- | --- | --- | --- | --- |
| Characteristics | Time point | |  | Cohort 1 | Cohort 2 |
|  |  |  |  | (n = 6) | (n = 6) |
| Systolic blood pressure (mmHg) | Day 1 | -0.5 h | Mean ± SD | 118.3 ± 6.0 | 119.3 ± 13.1 |
|  |  |  | Median | 121.0 | 123.0 |
|  |  |  | Range | 109-123 | 95-130 |
|  |  | 1 h | Mean ± SD | 119.8 ± 14.0 | 123.5 ± 14.0 |
|  |  |  | Median | 119.0 | 119.0 |
|  |  |  | Range | 103-142 | 109-147 |
|  |  | 2 h | Mean ± SD | 124.5 ± 16.9 | 120.7 ± 15.9 |
|  |  |  | Median | 130.5 | 116.0 |
|  |  |  | Range | 101-145 | 106-145 |
|  |  | 3 h | Mean ± SD | 118.0 ± 24.3 | 120.8 ± 21.7 |
|  |  |  | Median | 121.5 | 117.0 |
|  |  |  | Range | 90-146 | 98-159 |
|  |  | 4 h | Mean ± SD | 117.0 ± 12.1 | 109.7 ± 9.4 |
|  |  |  | Median | 115.5 | 106.0 |
|  |  |  | Range | 104-137 | 100-122 |
|  |  | 6 h | Mean ± SD | 111.3 ± 16.7 | 113.3 ± 24.9 |
|  |  |  | Median | 112.5 | 111.5 |
|  |  |  | Range | 88-136 | 88-146 |
|  | Day 8 |  | Mean ± SD | 111.3 ± 10.5 | 122.8 ± 8.4 |
|  |  |  | Median | 115.0 | 124.5 |
|  |  |  | Range | 102-126 | 108-132 |
|  | Day 14 | -0.5 h | Mean ± SD | 114.0 ± 11.1 | 116.2 ± 12.7 |
|  |  |  | Median | 117.5 | 119.0 |
|  |  |  | Range | 97-128 | 96-131 |
|  |  | 1 h | Mean ± SD | 119.0 ± 15.8 | 106.8 ± 12.1 |
|  |  |  | Median | 120.5 | 107.5 |
|  |  |  | Range | 97-138 | 93-120 |
|  |  | 2 h | Mean ± SD | 113.3 ± 11.2 | 115.0 ± 11.1 |
|  |  |  | Median | 117.0 | 111.5 |
|  |  |  | Range | 92-123 | 106-136 |
|  |  | 3 h | Mean ± SD | 123.8 ± 18.3 | 120.0 ± 11.9 |
|  |  |  | Median | 121.0 | 120.0 |
|  |  |  | Range | 104-153 | 103-138 |
|  |  | 4 h | Mean ± SD | 105.7 ± 10.0 | 119.0 ± 12.4 |
|  |  |  | Median | 106.5 | 119.5 |
|  |  |  | Range | 93-117 | 103-137 |
|  |  | 6 h | Mean ± SD | 108.8 ± 10.6 | 117.0 ± 22.7 |
|  |  |  | Median | 109.5 | 115.5 |
|  |  |  | Range | 94-125 | 86-156 |
|  |  | 10 h | Mean ± SD | 124.8 ± 10.3 | 111.7 ± 18.3 |
|  |  |  | Median | 127.0 | 116.0 |
|  |  |  | Range | 108-135 | 85-131 |
|  | Day 15 | 24 h | Mean ± SD | 120.8 ± 15.1 | 116.2 ± 18.1 |
|  |  |  | Median | 122.5 | 118.5 |
|  |  |  | Range | 97-138 | 92-141 |
|  | Day 21 |  | Mean ± SD | 115.2 ± 11.7 | 120.8 ± 14.2 |
|  |  |  | Median | 118.0 | 119.5 |
|  |  |  | Range | 95-127 | 106-136 |
| Diastolic blood pressure (mmHg) | Day 1 | -0.5 h | Mean ± SD | 65.0 ± 17.5 | 66.3 ± 10.7 |
|  |  |  | Median | 70.0 | 68.0 |
|  |  |  | Range | 34-84 | 52-82 |
|  |  | 1 h | Mean ± SD | 68.7 ± 12.7 | 68.5 ± 12.2 |
|  |  |  | Median | 72.5 | 67.5 |
|  |  |  | Range | 47-83 | 54-85 |
|  |  | 2 h | Mean ± SD | 72.5 ± 10.2 | 69.3 ± 9.6 |
|  |  |  | Median | 76.0 | 66.0 |
|  |  |  | Range | 57-83 | 61-87 |
|  |  | 3 h | Mean ± SD | 63.2 ± 16.5 | 59.0 ± 13.8 |
|  |  |  | Median | 65.0 | 62.5 |
|  |  |  | Range | 42-88 | 37-78 |
|  |  | 4 h | Mean ± SD | 64.2 ± 12.6 | 61.7 ± 13.5 |
|  |  |  | Median | 65.5 | 55.0 |
|  |  |  | Range | 50-84 | 51-85 |
|  |  | 6 h | Mean ± SD | 65.7 ± 16.8 | 65.5 ± 18.5 |
|  |  |  | Median | 74.0 | 69.0 |
|  |  |  | Range | 35-78 | 44-92 |
|  | Day 8 |  | Mean ± SD | 68.7 ± 5.7 | 76.5 ± 9.7 |
|  |  |  | Median | 69.0 | 77.0 |
|  |  |  | Range | 59-76 | 62-89 |
|  | Day 14 | -0.5 h | Mean ± SD | 69.7 ± 5.6 | 67.2 ± 12.6 |
|  |  |  | Median | 70.5 | 67.0 |
|  |  |  | Range | 61-77 | 47-82 |
|  |  | 1 h | Mean ± SD | 71.3 ± 10.2 | 56.0 ± 13.3 |
|  |  |  | Median | 76.0 | 54.5 |
|  |  |  | Range | 58-81 | 35-76 |
|  |  | 2 h | Mean ± SD | 61.3 ± 16.2 | 61.5 ± 11.4 |
|  |  |  | Median | 61.0 | 60.0 |
|  |  |  | Range | 33-81 | 49-82 |
|  |  | 3 h | Mean ± SD | 71.3 ± 12.3 | 69.7 ± 14.9 |
|  |  |  | Median | 73.0 | 66.0 |
|  |  |  | Range | 48-84 | 57-97 |
|  |  | 4 h | Mean ± SD | 56.0 ± 10.6 | 66.0 ± 5.2 |
|  |  |  | Median | 57.0 | 66.5 |
|  |  |  | Range | 43-67 | 59-72 |
|  |  | 6 h | Mean ± SD | 65.2 ± 14.9 | 65.3 ± 13.0 |
|  |  |  | Median | 63.5 | 69.5 |
|  |  |  | Range | 43-88 | 41-75 |
|  |  | 10 h | Mean ± SD | 77.8 ± 6.8 | 53.3 ± 12.9 |
|  |  |  | Median | 76.5 | 51.0 |
|  |  |  | Range | 70-89 | 37-74 |
|  | Day 15 | 24 h | Mean ± SD | 67.8 ± 11.9 | 63.3 ± 18.3 |
|  |  |  | Median | 70.0 | 70.0 |
|  |  |  | Range | 46-82 | 37-81 |
|  | Day 21 |  | Mean ± SD | 71.7 ± 8.5 | 76.5 ± 10.9 |
|  |  |  | Median | 70.5 | 76.5 |
|  |  |  | Range | 60-82 | 62-94 |
| Pulse rate (/min) | Day 1 | -0.5 h | Mean ± SD | 88.2 ± 12.0 | 96.8 ± 9.5 |
|  |  |  | Median | 90.0 | 96.0 |
|  |  |  | Range | 69-101 | 86-113 |
|  |  | 1 h | Mean ± SD | 88.0 ± 12.0 | 90.2 ± 8.9 |
|  |  |  | Median | 87.0 | 88.0 |
|  |  |  | Range | 70-107 | 81-102 |
|  |  | 2 h | Mean ± SD | 77.3 ± 13.5 | 89.7 ± 9.0 |
|  |  |  | Median | 80.0 | 92.0 |
|  |  |  | Range | 60-95 | 78-103 |
|  |  | 3 h | Mean ± SD | 81.2 ± 10.2 | 92.3 ± 13.7 |
|  |  |  | Median | 84.0 | 94.5 |
|  |  |  | Range | 66-93 | 72-111 |
|  |  | 4 h | Mean ± SD | 84.7 ± 10.4 | 93.7 ± 18.6 |
|  |  |  | Median | 87.0 | 98.0 |
|  |  |  | Range | 67-98 | 61-111 |
|  |  | 6 h | Mean ± SD | 87.7 ± 10.6 | 91.3 ± 11.1 |
|  |  |  | Median | 88.0 | 92.5 |
|  |  |  | Range | 69-100 | 75-103 |
|  | Day 8 |  | Mean ± SD | 85.0 ± 13.0 | 99.5 ± 14.5 |
|  |  |  | Median | 87.0 | 97.5 |
|  |  |  | Range | 63-101 | 84-122 |
|  | Day 14 | -0.5 h | Mean ± SD | 90.2 ± 15.9 | 100.7 ± 17.5 |
|  |  |  | Median | 91.0 | 92.5 |
|  |  |  | Range | 65-113 | 86-128 |
|  |  | 1 h | Mean ± SD | 85.8 ± 9.3 | 95.0 ± 10.9 |
|  |  |  | Median | 87.5 | 92.0 |
|  |  |  | Range | 69-95 | 82-109 |
|  |  | 2 h | Mean ± SD | 83.3 ± 10.1 | 93.5 ± 7.1 |
|  |  |  | Median | 84.0 | 94.0 |
|  |  |  | Range | 65-93 | 84-101 |
|  |  | 3 h | Mean ± SD | 80.5 ± 9.3 | 94.2 ± 11.7 |
|  |  |  | Median | 78.5 | 94.0 |
|  |  |  | Range | 67-93 | 81-107 |
|  |  | 4 h | Mean ± SD | 85.7 ± 9.3 | 98.8 ± 7.9 |
|  |  |  | Median | 86.5 | 96.5 |
|  |  |  | Range | 70-96 | 93-113 |
|  |  | 6 h | Mean ± SD | 86.8 ± 6.7 | 91.0 ± 5.3 |
|  |  |  | Median | 87.0 | 91.0 |
|  |  |  | Range | 77-95 | 84-98 |
|  |  | 10 h | Mean ± SD | 85.2 ± 9.3 | 91.3 ± 11.8 |
|  |  |  | Median | 85.5 | 86.0 |
|  |  |  | Range | 69-98 | 78-107 |
|  | Day 15 | 24 h | Mean ± SD | 93.8 ± 14.4 | 100.5 ± 10.8 |
|  |  |  | Median | 93.0 | 102.0 |
|  |  |  | Range | 69-111 | 84-117 |
|  | Day 21 |  | Mean ± SD | 86.8 ± 4.9 | 88.5 ± 9.8 |
|  |  |  | Median | 87.0 | 89.5 |
|  |  |  | Range | 81-94 | 71-98 |
| Body temperature (°C) | Day 1 | -0.5 h | Mean ± SD | 36.45 ± 0.52 | 36.60 ± 0.37 |
|  |  |  | Median | 36.70 | 36.50 |
|  |  |  | Range | 35.5-36.9 | 36.2-37.1 |
|  |  | 1 h | Mean ± SD | 36.52 ± 0.24 | 36.72 ± 0.25 |
|  |  |  | Median | 36.50 | 36.65 |
|  |  |  | Range | 36.1-36.8 | 36.4-37.1 |
|  |  | 2 h | Mean ± SD | 36.55 ± 0.33 | 36.40 ± 0.64 |
|  |  |  | Median | 36.55 | 36.70 |
|  |  |  | Range | 36.1-37.0 | 35.6-36.9 |
|  |  | 3 h | Mean ± SD | 36.42 ± 0.31 | 36.50 ± 0.50 |
|  |  |  | Median | 36.55 | 36.65 |
|  |  |  | Range | 35.9-36.7 | 35.7-37.0 |
|  |  | 4 h | Mean ± SD | 36.38 ± 0.72 | 36.57 ± 0.63 |
|  |  |  | Median | 36.50 | 36.55 |
|  |  |  | Range | 35.1-37.3 | 35.5-37.3 |
|  |  | 6 h | Mean ± SD | 36.80 ± 0.27 | 36.80 ± 0.34 |
|  |  |  | Median | 36.70 | 36.80 |
|  |  |  | Range | 36.6-37.3 | 36.3-37.3 |
|  | Day 8 |  | Mean ± SD | 36.30 ± 0.57 | 36.90 ± 0.28 |
|  |  |  | Median | 36.40 | 36.90 |
|  |  |  | Range | 35.4-36.9 | 36.4-37.2 |
|  | Day 14 | -0.5 h | Mean ± SD | 36.55 ± 0.39 | 36.55 ± 0.54 |
|  |  |  | Median | 36.50 | 36.40 |
|  |  |  | Range | 36.2-37.0 | 35.9-37.3 |
|  |  | 1 h | Mean ± SD | 36.27 ± 0.29 | 36.40 ± 0.26 |
|  |  |  | Median | 36.30 | 36.45 |
|  |  |  | Range | 35.9-36.7 | 36.0-36.7 |
|  |  | 2 h | Mean ± SD | 36.57 ± 0.31 | 36.28 ± 0.38 |
|  |  |  | Median | 36.55 | 36.25 |
|  |  |  | Range | 36.2-36.9 | 35.8-36.9 |
|  |  | 3 h | Mean ± SD | 36.45 ± 0.42 | 36.50 ± 0.36 |
|  |  |  | Median | 36.40 | 36.65 |
|  |  |  | Range | 35.9-37.0 | 36.0-36.8 |
|  |  | 4 h | Mean ± SD | 36.58 ± 0.22 | 36.30 ± 0.43 |
|  |  |  | Median | 36.60 | 36.15 |
|  |  |  | Range | 36.3-36.9 | 35.9-37.0 |
|  |  | 6 h | Mean ± SD | 36.45 ± 0.76 | 36.60 ± 0.50 |
|  |  |  | Median | 36.65 | 36.50 |
|  |  |  | Range | 35.0-37.2 | 36.1-37.3 |
|  |  | 10 h | Mean ± SD | 36.78 ± 0.43 | 36.45 ± 0.50 |
|  |  |  | Median | 36.85 | 36.50 |
|  |  |  | Range | 36.1-37.2 | 35.7-37.1 |
|  | Day 15 | 24 h | Mean ± SD | 36.55 ± 0.58 | 36.28 ± 0.54 |
|  |  |  | Median | 36.65 | 36.40 |
|  |  |  | Range | 35.6-37.1 | 35.3-36.8 |
|  | Day 21 |  | Mean ± SD | 36.68 ± 0.33 | 36.53 ± 0.44 |
|  |  |  | Median | 36.80 | 36.70 |
|  |  |  | Range | 36.1-37.0 | 36.0-37.1 |
